# Aminoacyl-tRNA Specificity of a Ligase Catalyzing Non-ribosomal Peptide Extension

**DOI:** 10.1021/jacs.5c12610

**Published:** 2025-10-09

**Authors:** Dinh T. Nguyen, Josseline S. Ramos-Figueroa, Alexander A. Vinogradov, Yuki Goto, Mayuresh G. Gadgil, Rebecca A. Splain, Hiroaki Suga, Wilfred A. van der Donk, Douglas A. Mitchell

**Affiliations:** † Carl R. Woese Institute for Genomic Biology, 14589 University of Illinois at Urbana−Champaign, 1206 West Gregory Drive, Urbana, Illinois 61801, United States; ‡ Department of Chemistry and Howard Hughes Medical Institute, University of Illinois at Urbana−Champaign, 600 South Mathews Avenue, Urbana, Illinois 61801, United States; § Department of Chemistry, Graduate School of Science, The University of Tokyo, 7-3-1, Hongo, Bunkyo, Tokyo 113-0033, Japan; ∥ Department of Biochemistry, Vanderbilt University School of Medicine − Basic Sciences, Nashville, Tennessee 37232, United States; ⊥ Department of Chemistry, 5718 Vanderbilt University, Nashville, Tennessee 37232, United States

## Abstract

Peptide aminoacyl-transfer ribonucleic acid ligases (PEARLs) are amide-bond-forming enzymes that extend the main chain of peptides by using aminoacyl-tRNA (aa-tRNA) as a substrate. In this study, we investigated the substrate specificity of the PEARL BhaB_C_^Ala^ from *Bacillus halodurans*, which utilizes Ala-tRNA^Ala^. By leveraging flexizyme, a ribozyme capable of charging diverse acids onto a desired tRNA, we generated an array of aa-tRNAs in which we varied both the amino acid and the tRNA to dissect the substrate scope of BhaB_C_^Ala^. We demonstrate that BhaB_C_^Ala^ catalyzes peptide extension with noncognate proteinogenic and noncanonical amino acids, hydroxy acids, and mercaptocarboxylic acids when attached to tRNA^Ala^. For most of these, the efficiency was considerably reduced compared to Ala, indicating that the enzyme recognizes the amino acid. By variation of the different parts of the tRNA, enzyme specificity was shown to also depend on the acceptor stem and the anticodon arm of the tRNA. These findings establish the molecular determinants of PEARL specificity and provide a foundation for engineering these enzymes for broader applications in peptide synthesis.

## Graphical abstract

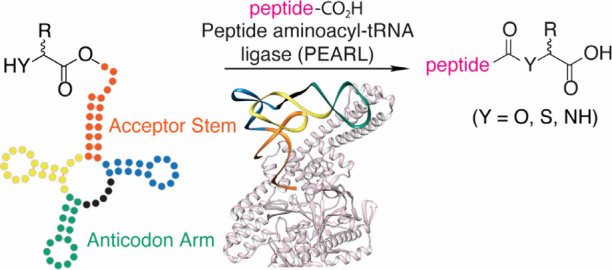

Amide bond formation is a critical process for the preparation of peptide- and protein-based therapeutics.^1–3^ Accessing these structures usually relies on solid-phase/liquid phase peptide synthesis or ribosomal translation.^4–7^ The recent increased interest in using biocatalysis presents opportunities to incorporate enzymes that form amide bonds into synthetic processes.^8–16^ Enzyme-based synthesis potentially offers scalability with high efficiency, selectivity, and waste minimization.^17–20^ One class of amide-bond-forming enzymes are the peptide aminoacyl-transfer ribonucleic acid ligases (PEARLs).^21–25^ PEARLs utilize aminoacyl-transfer ribonucleic acid (aa-tRNA) to form a new peptide bond at the C-terminus of a peptide (Figure 1A).^21,26^ Whereas the specificity for the peptide substrate has been investigated,^27^ the factors that determine the specificity for the aminoacyl-tRNA substrate are currently unresolved.

**Figure 1:**
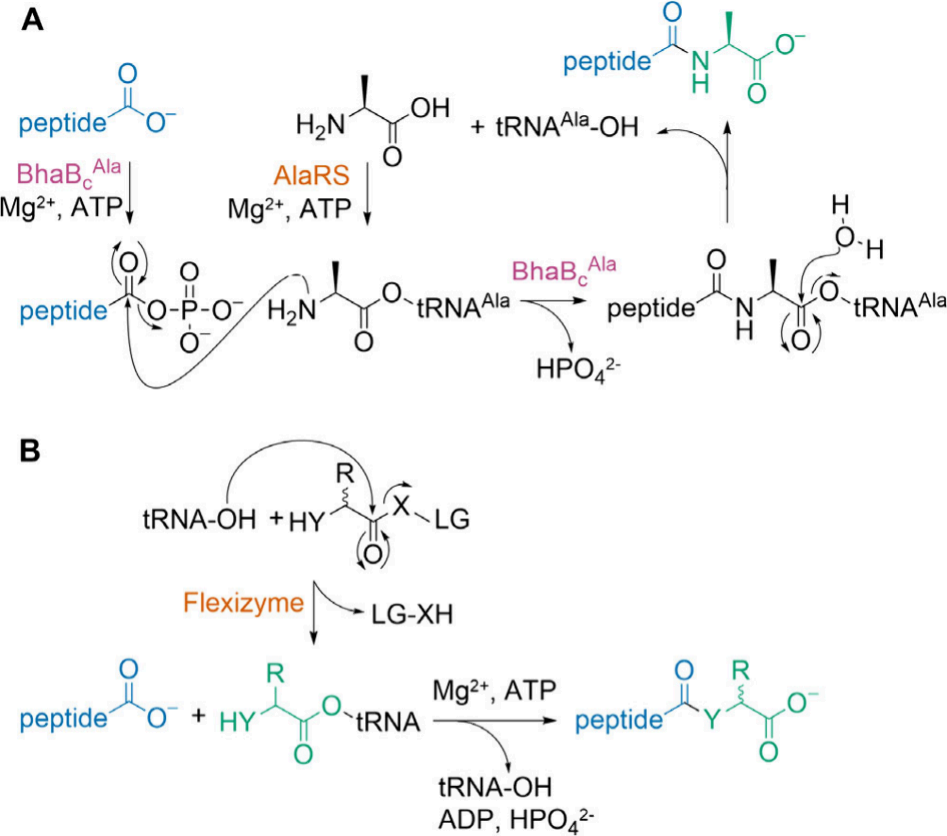
(A) The proposed mechanism of amide-bond formation catalyzed by PEARLs,^26^ in this case BhaB_C_^Ala^.^22^ Here, the cosubstrate Ala-tRNA^Ala^ is formed by AlaRS. (B) Examination of the substrate specificity of PEARLs using diverse aa-tRNA chimeras generated by flexizyme, the focus of this work. X-LG here represents 4-chlorobenzyl thioester, 3,5-dinitrobenzylester, or cyanomethyl ester, which are leaving groups recognized by flexizyme (details in Supporting Information and Table S1).

Here we investigate this specificity for a representative PEARL from *Bacillus halodurans*, BhaB_C_^Ala^ (NCBI accession identifier: BAB05753.1),^22^ which naturally utilizes Ala-tRNA^Ala^. Our data show that the combination of the correct amino acid and cognate tRNA ensures fidelity in the cellular context, with the anticodon arm and the acceptor stem predominantly determining the tRNA specificity of BhaB_C_^Ala^. The enzyme also recognizes the charged amino acid (i.e., attached to the tRNA), but it exhibits some tolerance that may be explored for engineering purposes.

In previous studies on PEARLs, where product formation was assessed through both *in vitro* assays and *in vivo* coexpression in *Escherichia coli*, the aa-tRNA was generated using aminoacyl-tRNA synthetases (aaRSs). Because aaRSs are highly specific toward their cognate aa-tRNAs, often containing editing domains that hydrolyze mischarged tRNAs,^28^ further exploration of the aa-tRNA specificity of BhaB_C_^Ala^ using aaRSs is limited to alanine and natural tRNA isoforms. Therefore, we first investigated whether both isoforms of tRNA^Ala^ in *E. coli* (GGC and UGC anticodons) were competent substrates for the BhaB_C_^Ala^-catalyzed addition of Ala to its substrate peptide BhaA (NCBI: WP_010898193.1) *in vitro*. Analysis by matrix-assisted laser desorption/ionization time-of-flight mass spectrometry indicated that both isoacceptors were substrates (Figure S1). The limitation of aaRSs to investigate substrate scope of PEARLs can be overcome using flexizymes, 45- or 46-nucleotide ribozymes that catalyze the formation of aminoacyl-tRNAs from diverse activated amino acids and tRNA sequences (Figure 1B).^29–36^ Using flexizymes, we investigated whether BhaB_C_^Ala^ can extend the substrate peptide with various acids attached to tRNA^Ala^. We also investigated the tolerance of BhaB_C_^Ala^ toward alanine attached to various noncognate tRNA sequences.

We first conducted BhaB_C_^Ala^ reactions using Ala-tRNA^Ala^(UGC) generated by flexizyme instead of AlaRS. The flexizyme reaction products were partially purified by ethanol precipitation, and the concentrations of aa-tRNA were estimated using a previously reported NaIO_4_-RNA extension assay (Figure S2).^37–39^ The reaction efficiency of BhaB_C_^Ala^ at different aa-tRNA concentrations was analyzed using liquid chromatography–electrospray ionization mass spectrometry (LC-ESI-MS). For all experiments, we used a truncated version of BhaA lacking the first 19 N-terminal residues (Δ19BhaA), which underwent quantitative modification by BhaB_C_^Ala^ when using Ala-tRNA^Ala^(UGC) generated by AlaRS (Figure S3). The BhaB_C_^Ala^ reaction efficiency increased with higher concentrations of Ala-tRNA^Ala^ prepared by flexizyme and reached complete modification. These observations demonstrate that the BhaB_C_^Ala^ enzymatic assay was compatible with flexizyme-prepared aa-tRNA (Figure S4), even though it contains significant amounts (∼65%, Figure S2) of non-aminoacylated tRNA that could serve as a competitive inhibitor (for a general discussion, see the Supporting Information – Materials and Methods).

Having established the use of flexizyme-prepared aa-tRNA in the BhaB_C_^Ala^ assay, we next examined whether cognate tRNA^Ala^(UGC) charged with different acids would be accepted by BhaB_C_^Ala^ (Figure 2). The selected acids, including proteinogenic and noncanonical amino acids, a hydroxy acid, and a mercaptocarboxylic acid, were first chemically activated (Table S1) and used as flexizyme substrates as previously reported.^40–46^ As described above for Ala-tRNA^Ala^, we first estimated the tRNA acylation efficiency with the various amino acids using NaIO_4_-RNA extension assays (Figure S5) and then conducted the BhaB_C_^Ala^ assays at various estimated aa-tRNA concentrations (Figures S6–S16). The highest observed conversion levels in end-point assays are shown in Figure 2.

**Figure 2:**
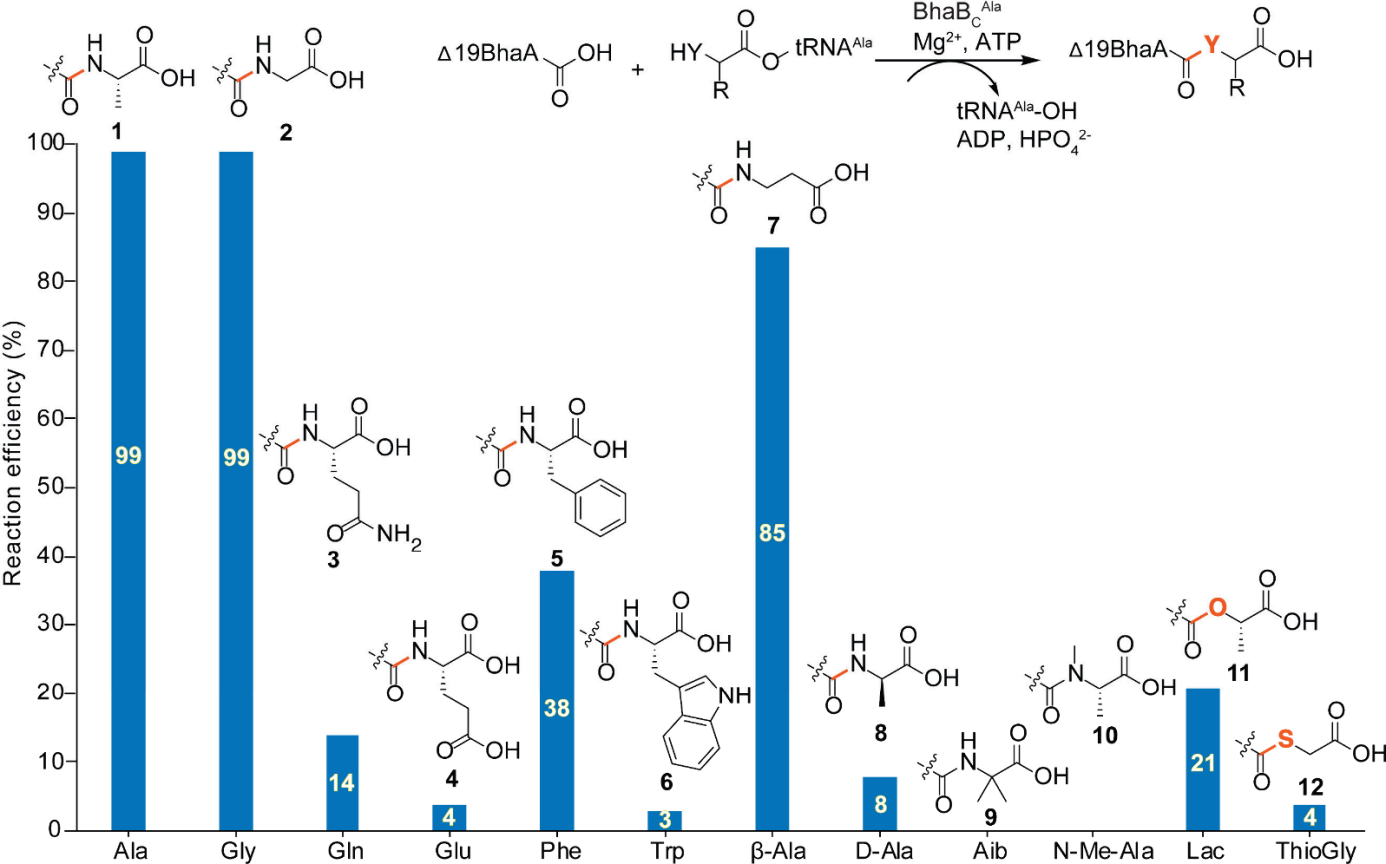
Amino acid specificity of BhaB_C_^Ala^. *E. coli* tRNA^Ala^ was charged with various acids using flexizyme. The acyl-tRNA^Ala^ chimeras were then tested for BhaB_C_^Ala^-catalyzed ligation with Δ19BhaA (1.8 μM, sequence in Figure S3). Reaction efficiencies were evaluated at multiple acyl-tRNA concentrations using LC-ESI-MS; the values shown represent the highest conversions observed. Products were not detected for Aib and *N*-Me-Ala. All extracted ion chromatograms (EICs) (with the estimated acyl-tRNA concentrations used to achieve the conversions in this figure in parentheses) are provided in Figures S4 (Ala, 4.7 μM), S6 (Gly, 14 μM**)**, S7 (Gln, 15 μM), S8 (Glu, 22 μM), S9 (Phe, 19 μM), S10 (Trp, 31 μM), S11 (β-Ala, 12 μM), S12 (d-Ala, 41 μM), S13 (Aib, 17 μM), S14 (N-Me-Ala, 40 μM), S15 (Lac), and S16 (ThioGly); Lac and ThioGly were not amenable to the NaIO_4_-RNA extension assay to estimate concentration.

BhaB_C_^Ala^ catalyzed amide formation with all evaluated proteogenic aminoacyl-tRNA^Ala^ analogs with a wide range of efficiencies. Gly-tRNA^Ala^ (**2**) was incorporated with efficiency akin to Ala-tRNA^Ala^ (**1**) whereas Phe-tRNA^Ala^ (**5**) was utilized with moderate activity. Although other proteinogenic amino acids (**3**, **4**, and **6**) attached to tRNA^Ala^ were accepted with significantly lower activity, the observed substrate tolerance is promising for future engineering applications. We next evaluated noncanonical amino acids attached to tRNA^Ala^ (Figures S11–S14). While BhaB_C_^Ala^ catalyzed efficient addition of β-Ala (**7**),^40^ much lower efficiency was observed with d-Ala (**8**).^41^ We did not observe peptide extension with 2-aminoisobutyric acid (Aib, **9**)^42^ or N-Me-Ala (**10**).^43^ BhaB_C_^Ala^ also catalyzed C–O and C–S bond formation when lactate (**11**) and thioglycolic acid (**12**), respectively, were attached to tRNA^Ala^(UGC) (Figures S15 and S16),^44,45^ although product formation was reduced compared to that observed with l-Ala and Gly. The resulting thioester product formed with (**12**) successfully underwent native chemical ligation^47^ with cysteamine (Figure S17). Collectively, these results show that BhaB_C_^Ala^ appears to have evolved to recognize l-Ala, but other small amino acids and hydroxy/mercaptocarboxylic acids attached to tRNA^Ala^(UGC) are also accepted, showing considerable tolerance with respect to the nucleophile. Directed evolution experiments on the enzyme may be able to increase the observed reaction efficiencies for desired substrates.^48^

The observation that various acyl groups attached to tRNA^Ala^(UGC) were substrates for BhaB_c_^Ala^ in vitro, whereas only extension with Ala is observed in *E. coli*,^22^ suggests that the enzyme also recognizes features of the tRNA. Since Gly-tRNA^Ala^ was an efficient substrate, we first tested whether Gly-tRNA^Gly^(GCC) generated by *E. coli* GlyRS was accepted by BhaB_C_^Ala^. This reaction resulted in only moderate conversion (Figure S18) while the reaction with Gly-tRNA^Ala^(UGC) at similar concentration of substrates gave quantitative modification (Figure S6), indicating that tRNA identity contributes to substrate recognition. The results described thus far suggest that BhaB_C_^Ala^ evolved to recognize Ala-tRNA^Ala^ *in vivo* ^22^ based on the combination of the amino acid and the tRNA component. To further examine this hypothesis, we used flexizyme to charge Ala onto various noncognate tRNAs having different sequences (Figures 3, S19 and S20, Table S2). The tRNAs for Trp, Glu, and Cys were chosen because they are substrates of previously reported PEARLs or PEARL-like enzymes.^21,22,49,50^ As described above, we first estimated the flexizyme-catalyzed aminoacylation efficiency of each tRNA with Ala using NaIO_4_-RNA extension assays (Figure S21) and then conducted the BhaB_C_^Ala^ assays at various estimated aa-tRNA concentrations (Figures S22–S24). The highest observed conversion levels in the end-point assays are shown in Figure 3. All Ala-tRNA chimera lead to lower BhaB_C_^Ala^ reaction efficiency compared to Ala-tRNA^Ala^, with *Pseudomonas syringae* Ala-tRNA^Cys^(GCA) (2.6%, Figure S22) and *E. coli* Ala-tRNA^Trp^(CCA) (5.9%, Figure S23) being particularly poor substrates.

**Figure 3:**
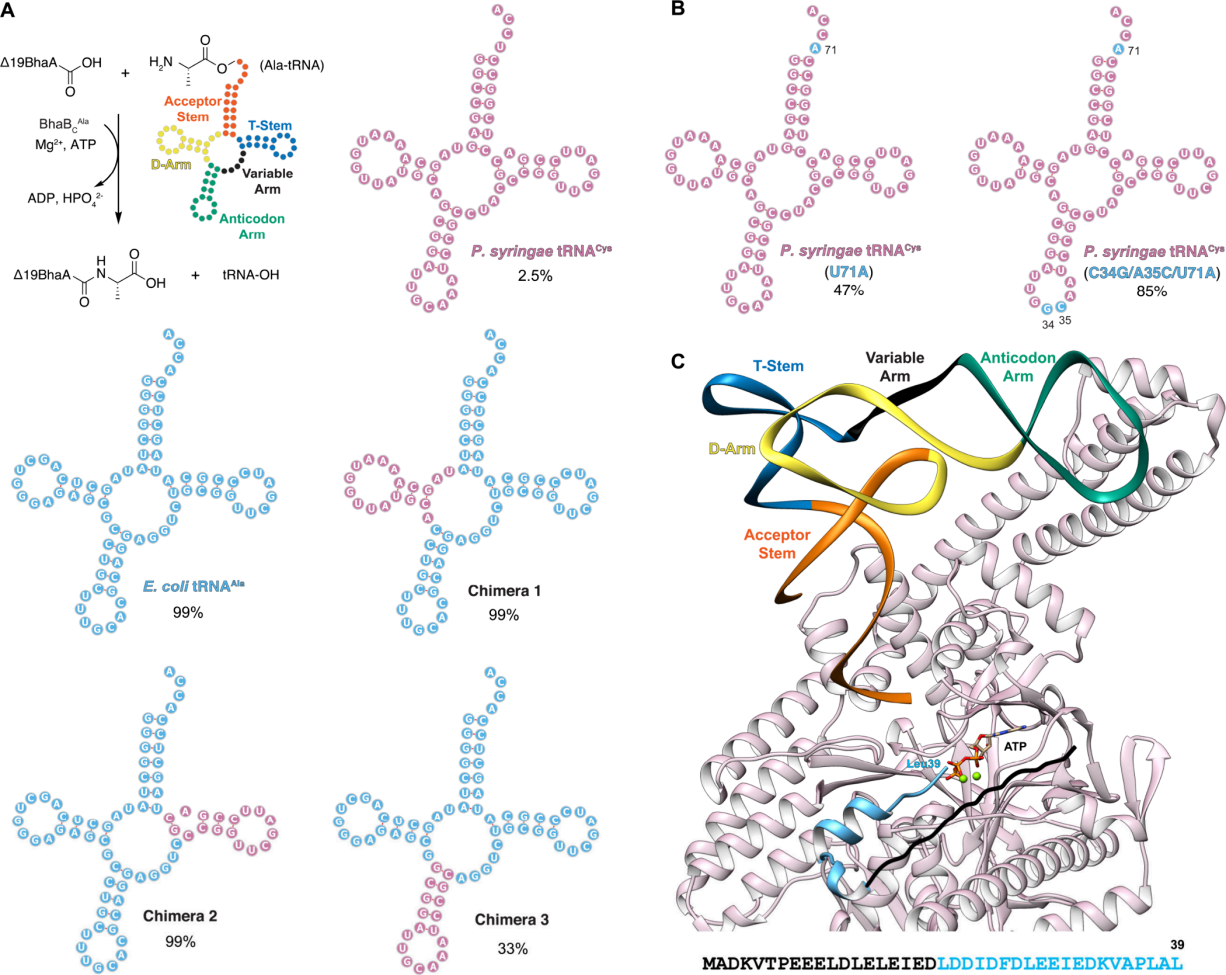
tRNA specificity of BhaB_C_^Ala^. (A) Various chimeric tRNAs with the D-arm, T-stem, or Anticodon Arm of *E. coli* tRNA^Ala^(UGC) and *P. syringae* tRNA^Cys^ (GCA) interchanged were aminoacylated with Ala using flexizyme. These Ala-tRNAs were used in the BhaB_C_^Ala^-catalyzed ligation with Δ19BhaA (sequence in Figure S3). (B) Mutations in *P. syringae* tRNA^Cys^(GCA) that considerably improved BhaB_C_^Ala^ activity. In panels A and B, regions derived from *E. coli* tRNA^Ala^ are in sky blue and regions from *P. syringae* tRNA^Cys^ are in purple. The cloverleaf structure of each tRNA was predicted using tRNAScanSE^54^ and Forna.^55^ Reactions were performed using a range of Ala-tRNA concentrations and analyzed using LC-ESI-MS; results are summarized in Table S2; values shown represent the highest estimated efficiencies observed. EICs are provided in Figure S4 (*E. coli* tRNA^Ala^), Figure S22 (*P. syringae* tRNA^Cys^), Figure S25 (Chimera 1), Figure S26 (Chimera 2), Figure S27 (Chimera 3), Figure S29 [*P. syringae* tRNA^Cys^ (U71A)], and Figure S30 [*P. syringae* tRNA^Cys^ (C34G/A35C/U71A)]. (C) AlphaFold3 model of the complex of BhaB_C_^Ala^, BhaA, *E. coli* tRNA^Ala^, Mg^2+^, and ATP. The tRNA is colored by structural region: acceptor stem (orange), D-arm (yellow), anticodon arm (green), variable arm (black), and T-stem (blue). The sequence of Δ19BhaA is colored light blue, with the first 19 residues present in full-length BhaA in black. Predicted local distance difference test (pLDDT) scores and predicted aligned error (pAE) plots for the model are provided in Figure S31 and Supplementary Data 1. The protein image was made using Chimera.^56^

To determine which regions of the tRNA were critical for BhaB_C_^Ala^ recognition, we used flexizyme to charge Ala onto chimeric tRNAs having individual regions of *E. coli* tRNA^Ala^(UGC) replaced with the corresponding sequences from the poor substrate *P. syringae* tRNA^Cys^(GCA) (Figures 3A, S20, S21, S25–S27, Table S2). We observed quantitative product formation with the chimera containing the D-arm (**Chimera 1**, Figure 3A) and T-stem (**Chimera 2**, Figure 3A) from tRNA^Cys^, albeit a reduction in efficiency was noted with the D-arm chimera at lower aa-tRNA concentrations (Figures 3A, S25, S26). In contrast, grafting the anticodon arm from tRNA^Cys^(GCA) into tRNA^Ala^(UGC) (**Chimera 3**, Figure 3A) significantly reduced activity, suggesting that the anticodon arm plays an important role in recognition (Figures 3A, S27). This conclusion was supported by introducing the anticodon arm from the poor substrate *E. coli* Ala-tRNA^Trp^(CCA) (Figure S23) into tRNA^Ala^(UGC), which also markedly decreased BhaB_C_^Ala^ activity (**Chimera 4**, Figure S28). These results provide an explanation for the poor acceptance of Ala-tRNA^Trp^(CCA) and Ala-tRNA^Cys^(GCA), but they do not explain why *Thermobispora bispora* Ala-tRNA^Glu^(CUC) was observed to be a more competent substrate (93%, Figure S24).

We next evaluated the acceptor stem for BhaB_C_^Ala^ activity, a region of importance for related aa-tRNA utilizing enzymes.^51^ A variant of the poor substrate *P. syringae* Ala-tRNA^Cys^ carrying a substitution in the acceptor stem [*P. syringae* tRNA^Cys^ (U71A), Figure 3B], introduced to match the discriminator base of *E. coli* tRNA^Ala^, showed significantly improved activity compared to the wild-type Ala-tRNA^Cys^(GCA) (Figures 3B, S29, Table S2). Activity was further enhanced when the second and third positions of the anticodon were mutated [*P. syringae* tRNA^Cys^ (C34G/A35C/U71A), Figure 3B] to match that of *E. coli* tRNA^Ala^ (Figures 3B, S30, Table S2), underscoring the importance of the anticodon. These findings also provide an explanation for Ala-tRNA^Glu^(CUC) being a moderately competent substrate. Unlike the poor substrates tRNA^Cys^ and tRNA^Trp^, this tRNA^Glu^ naturally has the same discriminator base and third base in the anticodon as tRNA^Ala^ (Figure S20). The collective results also agree well with an AlphaFold3 ^52^ model of BhaB_C_^Ala^, BhaA, and *E. coli* tRNA^Ala^(UGC), in which the enzyme primarily interacts with the acceptor stem and the anticodon arm of the tRNA (Figures 3C, S31). Similar interactions were previously observed in an Alphafold3 model of the PEARL BhaB_C_^Trp^ with *E. coli* tRNA^Trp^,^27^ and in a model of AmmB_4_^Arg^ with tRNA^Arg^.^53^ In support of these models, mutation of residues of BhaB_C_^Trp^ predicted to interact with the acceptor stem of the tRNA abolished enzyme activity.^27^ The model of AmmB_4_^Arg^ with tRNA^Arg^ (the first PEARL to use an amino acid with a charged side chain) also provided the first insights into the potential recognition of the aminoacyl group by the enzyme.^53^ However, high resolution visualization of the molecular interactions between the anticodon arm, acceptor stem, and aminoacyl group of aa-tRNA with residues on PEARLs will require structural biology studies. The AlphaFold3 model of BhaB_C_^Ala^ also shows that the first 19 residues of the substrate peptide do not interact with the protein, consistent with the findings above that these residues are not essential for BhaB_C_^Ala^ activity (Figures S3, S31, S32).

In summary, we used flexizyme to generate various noncognate aa-tRNA pairs and examined the substrate specificity of BhaB_C_^Ala^, an amide-forming enzyme that naturally uses Ala-tRNA^Ala^. Our data show that the observed specificity of BhaB_C_^Ala^ toward its cognate aa-tRNA *in vivo* ^22^ is determined by both the amino acid and the tRNA component. Since all of our tRNA molecules were obtained by *in vitro* transcription, we currently cannot rule out that post-transcriptional modifications may contribute further to this specificity. Despite this specificity, *in vitro* BhaB_C_^Ala^ can extend peptide chains with a range of amino acids and can catalyze bond formation beyond amides, such as the formation of esters and thioesters. Experimental and structural models indicate that BhaB_C_^Ala^ recognizes the tRNA through both the acceptor stem and the anticodon region with contributions from the discriminator base and the anticodon sequence. These findings lay the groundwork for future potential directed evolution of BhaB_C_^Ala^ variants capable of efficiently extending peptide chains with diverse amino acids beyond the native substrate.

## Supplementary Material





## Notes

Experimental procedures, Figures S1–S33 showing assay data and AlphaFold models, and Tables S1–S5 with nucleotide sequences, primers, and MS data (PDF)
Structure of BhaB_C_^Ala^–BhaA–tRNA–ATP complex predicted by AlphaFold3 (PDB)

## #

Department of Pharmacy and Pharmaceutical Sciences, Faculty of Science, National University of Singapore, Singapore 117544

## ¶

Department of Chemistry, Graduate School of Science, Kyoto University, Kitashirakawa-Oiwakecho, Sakyo, Kyoto 606-8502, Japan.
