# Supplementary material for: Aminoacyl-tRNA Specificity of a Ligase Catalyzing Non-ribosomal Peptide Extension
Source: J Am Chem Soc. 2025 Oct 9;147(42):37893–8. doi: 10.1021/jacs.5c12610 (PMC12550832; doi:10.1021/jacs.5c12610)
Supplement: Supplementary file 1 [file ja5c12610_si_001.pdf]

SUPPORTING INFORMATION

Aminoacyl-tRNA specificity of a ligase catalyzing non-ribosomal peptide extension

Dinh T. Nguyen,[1,2] Josseline S. Ramos-Figueroa,[2] Alexander A. Vinogradov,[3,4] Yuki Goto,[3,5] Mayuresh G. Gadgil,[2,6] Rebecca A. Splain,[2] Hiroaki Suga,[3] Wilfred A. van der Donk[1,2,]*, and Douglas A. Mitchell,[6,7]*

[1] Carl R. Woese Institute for Genomic Biology, University of Illinois at Urbana-Champaign, 1206 West Gregory Drive, Urbana, Illinois, 61801, USA.
[2] Department of Chemistry and Howard Hughes Medical Institute, 600 South Mathews Avenue, University of Illinois at Urbana−Champaign, Urbana, Illinois 61801, USA.
[3] Department of Chemistry, Graduate School of Science, The University of Tokyo, 7-3-1, Hongo, Bunkyo, Tokyo 113-0033, Japan
[4] Present address: Department of Pharmacy and Pharmaceutical Sciences, Faculty of Science, National University of Singapore, Singapore 117544
[5] Present address: Department of Chemistry, Graduate School of Science, Kyoto University, Kitashirakawa-Oiwakecho, Sakyo, Kyoto 606-8502, Japan
[6] Department of Biochemistry, Vanderbilt University School of Medicine – Basic Sciences, Nashville, TN 37232, USA
[7] Department of Chemistry, Vanderbilt University, Nashville, TN 37232, USA
* vddonk@illinois.edu, douglas.mitchell@vanderbilt.edu

MATERIALS AND METHODS.. 4

Figure S1. Mass spectrometry results of BhaA modified by BhaB$_C$^{Ala} using different isoforms of *E. coli* tRNA^{Ala}.. 12

Figure S2. The NaIO$_4$-RNA extension assay.. 13

Figure S3. Examination of Δ19BhaA as a substrate of BhaB$_C$^{Ala}... 14

Figure S4. EICs of Δ19BhaA modified by BhaB$_C$^{Ala} when reacting *E. coli* tRNA^{Ala} (UGC) charged with Ala using flexizyme.. 15

Figure S5. PAGE analysis of the NaIO$_4$-RNA extension assays (Figure S2) on flexizyme-catalyzed acylation products of *E. coli* tRNA^{Ala}(UGC) with various amino acids.................................... 16

Figure S6. EICs of Δ19BhaA modified by BhaB$_C$^{Ala} in the presence of *E. coli* tRNA^{Ala} (UGC) charged with Gly.. 17

Figure S7. EICs of Δ19BhaA modified by BhaB$_C$^{Ala} in the presence of *E. coli* tRNA^{Ala} (UGC) charged with Gln.. 18

Figure S8. EICs of Δ19BhaA modified by BhaB$_C$^{Ala} in the presence of *E. coli* tRNA^{Ala} (UGC) charged with Glu.. 19

Figure S9. EICs of Δ19BhaA modified by BhaB$_C$^{Ala} in the presence of *E. coli* tRNA^{Ala} (UGC) charged with Phe.. 20

Figure S10. EICs of Δ19BhaA modified by $BhaB_C^{Ala}$ in the presence of *E. coli* $tRNA^{Ala}$ (UGC) charged with Trp. 21

Figure S11. EICs of Δ19BhaA modified by $BhaB_C^{Ala}$ in the presence of *E. coli* $tRNA^{Ala}$ (UGC) charged with β-Ala. 22

Figure S12. EICs of Δ19BhaA modified by $BhaB_C^{Ala}$ in the presence of *E. coli* $tRNA^{Ala}$ (UGC) charged with D-Ala. 23

Figure S13. EICs of Δ19BhaA modified by $BhaB_C^{Ala}$ in the presence of *E. coli* $tRNA^{Ala}$ (UGC) charged with 2-aminoisobutyric acid (Aib). 24

Figure S14. EICs of Δ19BhaA modified by $BhaB_C^{Ala}$ in the presence of *E. coli* $tRNA^{Ala}$ (UGC) charged with N-Me-Ala. 25

Figure S15. EICs of Δ19BhaA modified by $BhaB_C^{Ala}$ in the presence of *E. coli* $tRNA^{Ala}$ (UGC) charged with Lac. 26

Figure S16. EICs of Δ19BhaA modified by $BhaB_C^{Ala}$ in the presence of *E. coli* $tRNA^{Ala}$ (UGC) charged with thioglycolic acid (ThioGly). 27

Figure S17. Native chemical ligation (NCL) of the peptide-thioester generated by $BhaB_C^{Ala}$ and ThioGly (Figure S16). 28

Figure S18. Δ19BhaA modified by $BhaB_C^{Ala}$ in the presence of *E. coli* $tRNA^{Gly}$(GCC) charged with Gly by *E. coli* GlyRS. 29

Figure S19. Predicted structures of examined tRNAs charged to Ala. 30

Figure S20. Sequence alignments of the tRNAs used to examine the Ala-tRNA specificity of $BhaB_C^{Ala}$. 31

Figure S21. (A) PAGE analysis of the $NaIO_4$-RNA extension assay (Figure S2) with different tRNAs charged with Ala by flexizyme. 32

Figure S22. EICs of Δ19BhaA modified by $BhaB_C^{Ala}$ in the presence of *P. syringae* $tRNA^{Cys}$ (GCA) charged with Ala. 33

Figure S23. EICs of Δ19BhaA modified by $BhaB_C^{Ala}$ in the presence of *E. coli* $tRNA^{Trp}$ charged with Ala 34

Figure S24. EICs of Δ19BhaA modified by $BhaB_C^{Ala}$ when reacting with Ala charged with *T. bispora* $tRNA^{Glu}$ (CUC). 35

Figure S25. EICs of Δ19BhaA modified by $BhaB_C^{Ala}$ when reacting with Ala charged with *E. coli* $tRNA^{Ala}$ (UGC) grafted with the D-arm of *P. syringae* $tRNA^{Cys}$ ($tRNA^{Ala\text{-}Cys(D)}$). 36

Figure S26. EICs of Δ19BhaA modified by $BhaB_C^{Ala}$ when reacting with Ala charged with *E. coli* $tRNA^{Ala}$ (UGC) grafted with the T-stem of *P. syringae* $tRNA^{Cys}$ ($tRNA^{Ala\text{-}Cys(T)}$). 37

Figure S27. EICs of Δ19BhaA modified by $BhaB_C^{Ala}$ when reacting with Ala charged to *E. coli* $tRNA^{Ala}$ (UGC) grafted with the anticodon Arm of *P. syringae* $tRNA^{Cys}$ ($tRNA^{Ala\text{-}Cys(Anti)}$). 38

Figure S28. EICs of Δ19BhaA modified by $BhaB_C^{Ala}$ when reacting with Ala charged to *E. coli* $tRNA^{Ala}$ (UGC) grafted with the anticodon arm of *E. coli* $tRNA^{Trp}$ ($tRNA^{Ala-Trp(Anti)}$). 39

Figure S29. EICs of Δ19BhaA modified by $BhaB_C^{Ala}$ when reacting with Ala charged onto *P. syringae* $tRNA^{Ala}$ (GCA) (U71A). 40

Figure S30. EICs of Δ19BhaA modified by $BhaB_C^{Ala}$ when reacting with Ala charged with *P. syringae* $tRNA^{Ala}$ (GCA) (C34G/A35C/U71A). 41

Figure S31. Alphafold3 model of the $BhaB_C^{Ala}$-BhaA-tRNA complex. 42

Figure S32. An active site view of the Alphafold3 model of BhaA, $BhaB_C^{Ala}$, ATP, Mg^{2+}, and *E. coli* $tRNA^{Ala}$(UGC) depicting that the first 19 residues of BhaA are not predicted to interact with $BhaB_C^{Ala}$. 43

Figure S33. (A) PAGE analysis of RNAs used in this study. 44

Table S1. Activated amino acids used in this study as substrates for flexizymes along with summary of tested flexizyme conditions 46

Table S2. Summary of $BhaB_C^{Ala}$ activity using non-cognate tRNAs charged with L-Ala. 47

Table S3. Sequence of the plasmid used to express $BhaB_C^{Ala}$ 48

Table S4. Sequences of tRNA molecules and the corresponding primers used for assembling templates for *in vitro* transcription 50

Table S5. Sequences of the Cy3-labeled primers used for the $NaIO_4$-RNA extension assays..... 52

Table S6. Calculated and observed *m/z* values for products and starting materials in this study. 53

REFERENCES 54

MATERIALS AND METHODS

Purification of $BhaB_C^{Ala}$

A previously constructed pET28-$BhaB_C^{Ala}$ plasmid (sequence in Table S3) was used to transform *E. coli* BL21(DE3) and the cells were selected on Luria Broth [10 g/L tryptone, 5 g/L yeast extract, 10 g/L NaCl] agar plates containing 50 µg/mL kanamycin (Kan), incubated at 37 °C for 12-14 h. Single colonies were picked and grown overnight in Terrific Broth [12 (g/L) tryptone, 24 (g/L) yeast extract, 0.072 M K_2HPO_4 and 0.017 M KH_2PO_4 and 0.4% glycerol (*v/v*)] containing 50 µg/mL kanamycin. The overnight culture was then diluted at a 1:100 (*v/v*) ratio in Terrific Broth containing the same amount of antibiotics in 1 L Fernbach flasks and grown at 37 °C with 220 rpm shaking until OD_{600} reached 1.5. Then, 2 mM $MgCl_2$ and 0.5 mM IPTG were added, and the culture was allowed to continue growth at 18 °C with shaking at 220 rpm for 18 h. The cells were then harvested by centrifugation at 7,000 × g for 10 min, with the supernatant being discarded, and the pellets were frozen at -80 °C prior to purification.

The cells were then resuspended in lysis buffer (50 mM 4-(2-hydroxyethyl)piperazine-1-ethanesulfonic acid [HEPES], 500 mM NaCl, 5% glycerol, pH 7.5) containing 4 µg/mL DNAse I, 4 mg/mL lysozyme, 2 µM E64, 2 µM benzamidine, 2 µM leupeptin, using 5 mL of buffer per grams of wet cells. The cells were then lysed using a high-pressure homogenizer (Avenstin, Inc) at 10 kpsi with three homogenizer cycles. The homogenate was subjected to centrifugation at 36,000 × g for 1 h, and every 70 mL of the supernatant was applied to 1 mL of Ni-NTA resin (HisPur, Thermo). The mixture was rocked for 30 min at 4 °C, after which the Ni-NTA resin (HisPur, Thermo) was loaded onto a column and washed with 15 column volumes (CV) of lysis buffer [containing 1 mM tris(2-carboxyethyl)phosphine (TCEP), but without protease inhibitors and DNAse I] followed by 15 CV of wash buffer (50 mM HEPES, 1 M NaCl, 5% glycerol, 30 mM imidazole pH 7.5, 1 mM TCEP). The protein was then eluted using 6 CV of elution buffer (50 mM HEPES, 300 mM NaCl, 250 mM imidazole, pH 7.5, 1 mM TCEP). The protein was then buffer-exchanged to storage buffer 1 (50 mM HEPES, 100 mM NaCl, 2.5% glycerol, 1 mM TCEP pH 7.0) using a Slide-A-Lyzer G3 dialysis cassette (20 kDa molecular weight cutoff, MWCO) with at least a 100x volume exchange.

After dialysis, the protein mixture was clarified by centrifugation at 16,000 × g for 10 min. The supernatant was then injected into a 5 mL HiTrap SP HP (Cytiva) cation exchange column connected to an ÄKTA Start chromatography system. Solvent A consisted of 50 mM HEPES, 100 mM NaCl, 2.5% glycerol, and 1 mM TCEP, pH 7.0, while solvent B consisted of 50 mM HEPES, 1 M NaCl, 2.5% glycerol, and 1 mM TCEP, pH 7.0. The system was run at a flow rate of 5 mL/min, and the column was pre-equilibrated with solvent A before injection. After sample injection, the column was washed with 6 CV of solvent A. Following washing, a gradient elution was performed from 100% solvent A and 0% solvent B to 0% solvent A and 100% solvent B over 20 CV, with 4 mL fractions collected. Each fraction was then subjected to Sodium Dodecyl Sulfate Polyacrylamide gel electrophoresis (SDS-PAGE) using a 4–20% precast polyacrylamide gel (Biorad) and ran at 250 V for 30 min. Fractions corresponding to peaks containing $BhaB_C^{Ala}$ were then subjected to a $BhaB_C^{Ala}$ assay with and without AlaRS (see $BhaB_C^{Ala}$ assays with AlaRS) to

identify those with minimal *E. coli* AlaRS carryover. Fractions with minimal carryover were pooled and concentrated to less than 2 mL using a 15 mL Amicon 30 kDa MWCO Ultra centrifugal filter (EMD Millipore).

Then 1 mL of the concentrated $BhaB_C^{Ala}$ was injected into a HiLoad 16/600 Superdex 200 pg size exclusion column pre-equilibrated with storage buffer 2 (50 mM HEPES, 300 mM NaCl, 2.5% glycerol, 1 mM TCEP, pH 7.0) on an ÄKTA Go system. The column was then run with an isocratic flow of 1 column volume (120 mL) using storage buffer 2 at a flow rate of 1.6 mL/min. Fractions were collected in 2 mL aliquots. Fractions corresponding to peaks containing $BhaB_C^{Ala}$ were subjected to a $BhaB_C^{Ala}$ assay $BhaB_C^{Ala}$ assay with and without AlaRS (see $BhaB_C^{Ala}$ assays with AlaRS) to identify those with minimal *E. coli* AlaRS carryover. Fractions with no AlaRS carryover were pooled (Figure S3), concentrated using a 4 mL Amicon 30 kDa MWCO Ultra centrifugal filter (EMD Millipore), flash frozen, and stored at -80 °C.

In vitro transcription and purification of RNAs (flexizyme and tRNAs)

The double-stranded DNA (dsDNA) templates for *in vitro* transcription were generated from overlapping primers[1] (Integrated DNA technologies) with sequences provided in Table S4. Specifically, 5' overhangs were assembled using the following extension reaction: 1 × NEB buffer 2, 4 µM of forward and reverse primers, 100 µM of each dNTPs, and 1 U of DNA polymerase I large (Klenow) fragment per 1 µg of DNA. These 50 µL reactions were incubated at 25°C for 15 min and then quenched with 10 mM EDTA at 75°C for 25 min. The DNA templates were cleaned by adding 5 µL (1/10 reaction volume) of 3 M NaOAc (pH 5.2) and 125 µL of cold ethanol, followed by incubation at 4 °C. The DNA pellet was collected by centrifugation at 16,000 × g for 15 min, and the supernatant was discarded. The pellet was washed twice with 500 µL of 70% ethanol and then air-dried for 15 min. Once dried, the pellet was dissolved in 12.5 µL of H_2O and prepared for *in vitro* transcription.

The *in vitro* transcription reaction was performed using RNase-free reagents with the following components: 100 mM tris (pH 7.5), 7.5 mM each rNTP, 36 mM $MgCl_2$, 50 mM DTT, 0.1 mg/mL bovine serum albumin, 0.8 U/µL RNase inhibitor (RiboLock), 0.5 mU/µL *E. coli* inorganic phosphatase (Thermo Scientific), and 0.9 mg/mL T7 RNA polymerase. The DNA pellet (12.5 µL per 125 µL in vitro transcription reaction) was added to the transcription mix. The reaction was incubated at 37 °C overnight. To remove the DNA, 6.25 U of DNase I (Thermo Scientific) per 125 µL reaction and 1× DNase I reaction buffer with $MgCl_2$ (Thermo Scientific) were added.

The tRNAs were then purified using an RNA Monarch Kit (500 µg) (NEB) per instructions of the manufacturer. One spin column was used for each 125 µL of *in vitro* transcription reaction. The tRNAs were eluted from the spin column using 75 µL of RNAse-free H_2O. Then, 2 mM NaOAc pH 5.2 was added to the eluted tRNAs. The concentration of the tRNAs was assayed using the Qubit RNA Broad Range Assay Kit (ThermoFisher Scientific) on a Qubit 2.0 fluorometer (ThermoFisher Scientific).

Flexizymes (from 1 mL *in vitro* transcription reactions) were purified by adding 1/10 volume of 5 M NH_4OAc, 1/20 volume of 0.5 M EDTA, and an equal volume of isopropanol. After centrifugation at 16,000 × g for 10 min, the supernatant was removed, and the RNA pellet was washed with 800 µL of 70% ethanol. The RNA was then air-dried (covered with a Kimwipe) before being dissolved in 100 µL of H_2O and added to an Amicon 10 kDa MWCO Ultra centrifugal filter (EMD Millipore). Following a 1,000× volume exchange with RNase-free H_2O, the RNA was purified from proteins using acidic phenol extraction. Specifically, an equal volume of acid-phenol: chloroform: isoamyl alcohol (25:24:1, pH 4.5) (Invitrogen) was added, and the mixture was shaken vigorously and centrifuged at 16,000 × g for 5 min. The aqueous layer was collected, and the organic layer was extracted with an equal volume of 0.3 M NaOAc (pH 5.2). The aqueous layers were combined and extracted again using the same protocol. Chloroform: isoamyl alcohol (24:1) (Sigma) was added to the collected aqueous layers, and the mixture was shaken vigorously and subjected to centrifugation at 16,000 × g for 5 min. The aqueous layer was collected and precipitated by adding 1/10 volume of 3 M NaOAc (pH 5.2) and 2.2 volumes of ethanol. The RNA pellet was collected by centrifugation at 16,000 × g for 15 min and washed twice with 75% ethanol. After removing the supernatant, the pellet was air-dried for 15 min (covered with a Kimwipe) before being redissolved in 2 mM NaOAc (pH 5.2). The concentration of the tRNAs was assayed using the Qubit RNA Broad Range Assay Kit (ThermoFisher Scientific) on a Qubit 2.0 fluorometer (ThermoFisher Scientific).

The quality of all RNAs was assessed using denaturing Urea-PAGE. Specifically, the tRNAs after eluting from the column were diluted 50 times using H_2O. 2 µL of diluted RNAs [concentration 70-270 (ng/µL)] was then mixed with 18 µL of 2x RNA loading dye (NEB) and heated at 95 °C for 3 min. Then, 3 µL was loaded onto 10% Mini-PROTEAN TBE-Urea Gel (Biorad) preheated at 250V for 30 min. The gel was run at 180 V for 36 min and stained with SYBR Gold (Thermo) before being visualized using the UV channel on ImageQuant800 (Cytiva) (Figure S33).

Flexizyme assays to prepare aa-tRNAs

The reactions were prepared by mixing 50 mM HEPES-KOH pH 7.5 (or 150 mM HEPES-NaOH pH 7.5), 41-61 µM eFx or 24 µM dFx,[2,3] 27-31 µM tRNA, 100 mM $MgCl_2$ for dFx (600 mM in case of Aib-DBE and Thiogly-DBE) or 400-600 mM $MgCl_2$ for eFx, and 5 mM activated amino acids. The amino acids (see Table S1) were activated by chemical synthesis with 4-chlorobenzyl thioester (CBT, used with eFx), 3,5-dinitrobenzyl ester (DBE, used with dFx), or cyanomethyl ester (CME, used with eFx) according to published protocols, purified, and stored in DMSO.[2,4-8] For a 110 µL reaction, the detailed protocol for charging of tRNA was as follows: First, HEPES, flexizyme, tRNA, and H_2O were mixed well together up to 66 µL in a 1.7 mL Eppendorf tube. The mixture was heated to 95 °C in an aluminum heating block for 2 min and then cooled down at room temperature on air for 7 min. Next, 22 µL of 500 mM – 3 M $MgCl_2$ stock was added, and the mixture was incubated at room temperature for 5 min. The reaction was

placed on ice for 3 min, after which 22 µL of 25 mM activated amino acid (stored in DMSO) was added. The reactions were incubated on ice with time specified in Table S1.

When significant precipitation was observed due to amino acid insolubility, DMSO was added to improve solubility. For a 110 µL Lac-CBT reaction, 22 µL of DMSO along with 5 µL of 500 mM HEPES (pH 7.5) were added to the reaction mixture after adding Lac-CBT. In the case of thioglycolate-CBT (Thiogly-CBT), the reaction was run in 150 mM Bicine pH 9 containing 5 mM DTT and 40% (*v/v*) DMSO instead of 20% (*v/v*) DMSO.

The RNA clean-up protocol was performed at room temperature as follows, with examples of volumes of solutions applied to each step described in the table below. First, the flexizyme reactions were quenched by adding 0.3 M NaOAc (pH 5.2). The resulting mixture was divided into two tubes, one for downstream $NaIO_4$-RNA extension assays and one for downstream $BhaB_C^{Ala}$ assays, with volumes specified in the accompanying table. EtOH was added to the quenched reactions, which were then centrifuged at 16,000 × g for 15 min. The supernatant was discarded, and the pellet was washed by adding 70% EtOH 0.1 M NaOAc (pH 5.2) (made by mixing aqueous 3M NaOAc pH 5.2 with ethanol and water) and vortexed. The mixture was centrifuged again at 16,000 × g for 5 min, and the wash was repeated. The pellet was then washed with 70% EtOH 1 mM NaOAc (pH 5.2) (diluted from 70% EtOH 0.1 M NaOAc (pH 5.2) with 70% EtOH) and centrifuged at 16,000 × g for 3 min. The supernatant was discarded, and the pellet was air-dried for 15 min (covered with a Kimwipe). The pellets were stored at -80 °C for a maximum of 5 days until use. A table describing the specific volumes is shown below.

		0.3 M NaOAc pH 5.2	EtOH	70% EtOH 0.1 M NaOAc (pH 5.2)	70% EtOH 1 mM NaOAc (pH 5.2)
Flexizyme reaction (total)	110 µL	Add 440 µL. Total volume = 550 µL			
For $BhaB_C^{Ala}$ assays		500 µL	1000 µL	600 µL	400 µL
For $NaIO_4$ - RNA extension assays		50 µL	100 µL	60 µL	40 µL

$NaIO_4$-RNA extension assays to measure tRNA aminoacylation[9-11]

The schematic of this assay is described in Figure S2. The RNA pellets cleaned up from 10 µL of Fx reactions were dissolved in 15 µL of 1 mM NaOAc pH 5.2. The solution was then split into three Eppendorf tubes, each containing 5 µL of RNA, to run three reactions corresponding to Schemes 1, 2, and 3 in Figure S2.

In the tube for Scheme 1: 42.5 µL of RNA buffer (50 mM NaOAc, pH 5.2, 150 mM NaCl, 10 mM $MgCl_2$, and 0.1 mM EDTA) was added to the RNA solution. Then, 2.5 µL of 100 mM $NaIO_4$ was added, and the reaction was incubated for 1 h at 10 °C. The reaction was quenched by adding 10 µL of 100 mM DTT and left on ice.

In the tube for Scheme 2: 45 µL of the RNA buffer was added to the RNA solution.

In the tube for Scheme 3: 40 µL of 1× deacylation buffer (50 mM Bicine-NaOH pH 9.7, and 1 mM EDTA) was added to the RNA solution, and the mixture was incubated at 42 °C in an aluminum heating block for 36 min. The reaction was quenched by adding 6 µL of 3 M NaOAc. Then, 2.7 µL of 100 mM $NaIO_4$ was added, and the reaction was incubated for 1 h at 10 °C. The reaction was quenched by adding 10 µL of 100 mM DTT and left on ice for 20 min.

All three mixtures were then purified using the Monarch RNA cleanup kit (10 µg) following the protocol of the manufacturer. The processed RNAs were then eluted from the spin columns using 12 µL of RNAse-free H_2O. The RNAs were then quantified using A_{260} using a spectrophotometer.

The purified RNAs were subjected to the RNA extension reaction as follows: 1× NEB Buffer 2, 500–750 nM RNA, 1 µM Cy3-labeled primer (see Table S5), 400 µM of each dNTP (Goldbio), and H_2O to bring the total volume to 23.5-24.5 µL in a 0.2 mL PCR tube. The mixture was subjected to the following cycle on a thermocycler: 95°C for 2 min, 70°C for 2 min, 50°C for 2 min, and then held at 4 °C. Next, 0.5-1.5 µL of Klenow Fragment (3'→5' exo-) (M0212S) was added, and the reaction was incubated at 37 °C for 45 min. An equal volume of 2× loading dye (8 M urea, 0.06% Orange G) was added to the reaction mixture.

The resulting mixture was subjected to PAGE using a 15% Mini-Protean TBE-Urea gel, 10-well, which was pre-run at 250 V for 25-30 min in 1× TBE buffer. Each well was rinsed thoroughly with the running buffer, and immediately afterwards, a 10 µL aliquot of the reaction mixture was loaded into the wells. The gel was run at 200 V for 150-180 min. The gel was then imaged using the Cy3 fluorescence channel on the ImageQuant800 (Cytiva) fluorescence application. The bands in each lane, which represented assayed free and aminoacylated tRNAs, were estimated relative to each other using ImageJ based on peak height. This estimation and the concentration of free tRNAs applied to the flexizyme assay were used to estimate the concentration of aminoacyl tRNAs being applied to the $BhaB_C^{Ala}$ reaction.

$BhaB_C^{Ala}$ assays with AlaRS

The concentration of a synthetic Δ19BhaA (1.3 mM; Genscript) solution in a buffered D_2O solution (600 µL of 100 mM potassium phosphate buffer at pH 7.5 resuspended in D_2O) was quantified using quantitative ^1H NMR (qNMR) on a Bruker Avance NEO 600 MHz spectrometer equipped with a 5-mm BBO prodigy probe, following standard protocols.[12] The solution was measured inside a 5-mm Wilmad 535-pp NMR tube. A solution of 18 µM of Δ19BhaA was mixed well with 100 mM HEPES-NaOH (pH 7.5), 100 mM NaCl, 5 mM $MgCl_2$, 5 mM ATP (from 50 mM ATP stock with pH adjusted to 7.5 prior to usage), 1 mM TCEP (from 100 mM TCEP stock with pH adjusted to 7.0 prior to usage), 5 mM L-Ala, and 13 µM *E. coli* $tRNA^{Ala}$ (UGC). If AlaRS was used, 10 µM *E. coli* AlaRS, purified according to a published protocol,[13] was added to the reaction mixture, and mixed thoroughly. Then, 10 µM of $BhaB_C^{Ala}$ was added, and the reaction proceeded at room temperature overnight. Two volumes of cold acetonitrile (put at -20 °C prior to usage) were then added to the reaction mixture, and precipitates were removed by centrifugation at 16,000 × g for 10 min. The solvents from the collected supernatant were removed by rotary

evaporation using a SpeedVac concentrator (Thermo). The resulting dried powder was then dissolved in an equal volume of H_2O to the original reaction volume. Then, 1 µL was subjected to mass spectral analysis (see Mass spectral analysis of the $BhaB_C^{Ala}$ assays).

Sequences:

BhaA: `MADKVTPEEELDLELEIEDLDDIDFDLEEIEDKVAPLAL`

Δ19BhaA: `LDDIDFDLEEIEDKVAPLAL`

$BhaB_C^{Ala}$ assays with Gly-$tRNA^{Gly}$ generated from GlyRS

In the case GlyRS was used to generate Gly-$tRNA^{Gly}$ (GCC), GlyRS was purified by Ni-affinity chromatography as reported in previous protocols.[13] 5 µM *E. coli* GlyRS was used under similar concentrations as mentioned above for AlaRS and the reaction was incubated for 4 h at 37 °C. The reaction mixture was purified by Monarch RNA spin column according to the manufacturer protocol and the aminoacylated tRNA was used in $BhaB_C^{Ala}$ reactions as indicated below. An aliquot of the aminoacylated $tRNA^{Gly}$ was utilized in $NaIO_4$-RNA extension reactions to estimate the degree of aminoacylation. The 25 µL reaction mixture with $BhaB_C^{Ala}$ contained: 100 mM HEPES (pH 7.5), 100 mM NaCl, 5 mM ATP (from 50 mM ATP stock with pH adjusted to 7.5 prior to usage), 1 mM TCEP (from 100 mM TCEP stock with pH adjusted to 7.0 prior to usage), 5 mM $MgCl_2$, 1.6 µM Δ19BhaA (concentration determined by qNMR as described above), 8.8 µM Gly-$tRNA^{Gly}$ (as determined by the estimated aminoacylation efficiency), and 4 µM $BhaB_C^{Ala}$. This mixture was incubated overnight at RT, quenched with an equal volume of cold acetonitrile (put at -20 °C prior to usage), subjected to centrifugation at 16,000 × g for 5 min, and an aliquot of 5 µL submitted for LC-MS analysis.

$BhaB_C^{Ala}$ assays with aa-tRNA prepared by flexizyme

The reaction mixtures contained: 100 mM HEPES (pH 7.5), 100 mM NaCl, 5 mM ATP (from 50 mM ATP stock with pH adjusted to 7.5 prior to usage), 1 mM TCEP (from 100 mM TCEP stock with pH adjusted to 7.0 prior to usage), 5 mM $MgCl_2$, 1.8 µM Δ19BhaA (concentration determined by qNMR as described above), $BhaB_C^{Ala}$ (4 µM for non-cognate amino acid experiments and 6 µM for non-cognate tRNA experiments), and serial dilutions of aa-tRNA concentrations. The aminoacylation efficiency of the tRNA, estimated from $NaIO_4$-RNA extension assays (Figures S2, S5, S21), was used to estimate the amount of aa-tRNA applied in the $BhaB_C^{Ala}$ assays. The concentrations of aa-tRNA are shown in the extracted ion chromatograms (EIC) shown in Figures S4, S6-S16, and S22-S30. The detailed protocol for 10 µL reactions was as follows. First, a mixture of buffer-cofactor-peptide was assembled by mixing HEPES, NaCl, ATP, TCEP, $MgCl_2$, Δ19BhaA, and H_2O to a volume of 7.7 µL. The aa-tRNA pellet (from ethanol precipitation of 50-100 µL flexizyme reactions as described above) was resuspended in 9.8 µL of 1 mM NaOAc (pH 5.2) and added to the mixture, bringing the volume to 17.5 µL. To perform serial dilutions of aa-tRNAs, four additional buffer-cofactor-peptide mixtures (7.7 µL each) were prepared as above, and 9.8 µL of 1 mM NaOAc (pH 5.2) was added instead of aa-tRNA. 8.75 µL of the aa-tRNA-containing mixture was then mixed in 8.75 µL with each of the four buffer-cofactor-peptide (no

aa-tRNA) mixtures in sequence to achieve serial 2-fold dilutions. This procedure resulted in five reaction tubes, each with a total volume of 8.75 µL and identical amounts of all components except aa-tRNA. Then, 1.25 µL $BhaB_C^{Ala}$ (32 µM for non-cognate amino acid experiments and 48 µM for non-cognate tRNA experiments), purified via ion-exchange chromatography followed by size-exclusion chromatography as mentioned above, was added to each reaction mixture, and the reactions were incubated overnight at room temperature. In the case of thiogly-$tRNA^{Ala}$, an additional reaction containing 1 mM cysteamine (pH 7.5) was performed to assess native chemical ligation with the resulting thioester. A 6 µL aliquot of each reaction was then injected directly into the column or subjected to acetonitrile precipitation prior to mass spectral analysis. Specifically, 4 µL aliquot of the reaction was mixed with 8 µL of cold acetonitrile (put at -20 °C prior to usage), and precipitates were removed by centrifugation at 16,000 × g for 10 min. 6 µL of the clarified supernatants were then subjected to mass spectral analysis.

All flexizyme reaction mixtures used in the $BhaB_C^{Ala}$ assay after ethanol precipitation contained not only acylated tRNA but also non-acylated tRNA, which could reduce the reaction rate through competitive inhibition. Since we observed complete peptide extension with *E. coli* Ala-$tRNA^{Ala}$(UGC) prepared by flexizyme, any potential inhibition did not prevent full conversion. Because acylation efficiency varied between the acyl-tRNAs investigated (Figures S5 and S21), the proportion of non-acylated tRNA differed across substrates and may have influenced their reaction efficiencies to varying degrees. However, as the reaction efficiency for each substrate increased or saturated at higher concentrations of acylated tRNA (conditions that also contain proportionally higher levels of non-acylated tRNA, because solutions of different acyl-tRNA concentrations were generated by serial dilution), potential competitive inhibition by non-acylated tRNA did not preclude assessment of substrate scope using flexizyme-prepared aa-tRNA partially purified by ethanol precipitation. We cannot rule out that methods to remove non-acylated tRNA or recharge non-acylated tRNA to the acylated form as occurs with AlaRS (which can only generate cognate Ala-$tRNA^{Ala}$ and not other acyl-tRNA pairs successfully examined using flexizyme) may enhance reaction efficiency for certain acylated tRNAs beyond the levels shown in this work, but such approaches would require evolved tRNA-tRNA synthetase pairs that were beyond the scope of the current work.

Mass spectral analysis of the $BhaB_C^{Ala}$ assays

Reactions to be analyzed were injected into a Kinetex C8 LC column (2.1 × 150 mm, 2.6 µm, 100 Å) on an Agilent 6545B Q-TOF interfaced with an Agilent 1290 Infinity II LC system. The mobile phase was: solvent A: H_2O with 0.1% formic acid (FA), solvent B: CH_3CN with 0.1% FA. The flow rate was 0.4 mL/min. The gradient was as follows: first 3 min 5% B, next 8 min 5% B - 80% B, last 2 min 80–95% B. Before the next samples were injected, the column was equilibrated with 3 min of 5% B. The samples were run to waste in the first 4 min before applying to the mass spectrometer. MS parameters were as follows: mass range 100 to 3000 *m/z*; 325 °C at 10 L/min; nebulizer, 35 psig; nozzle voltage, 0 V; sheath gas, 375 °C at 11 L/min; capillary, 3500 V; fragmentor, 120 V; skimmer, 65 V; MS scan rate (10 spectra/s). Positive ionization mode was

utilized for all MS experiments. The EICs of species corresponding to the substrate and products were generated based on the *m/z* (z= 2) of each peptide. The reaction efficiency (x %) was estimated using the peak area of each EIC (A) as follows: $x = [A_{product} /(A_{product} + A_{substrate})]$ (%), with the assumption that the ionization efficiency of the substrate and the product is very similar, as in other similar studies.[14,15] This assumption is supported by the highly similar chemical nature of the substrates and products because their relative masses are very close and the reactions do not introduce additional positively charged moieties. However, due to the complex nature of ESI-MS causing potential subtle differences in ionization efficiency of different peptides,[16,17] the method should be considered semi-quantitative.

Figure S1. Mass spectrometry results of BhaA modified by $BhaB_C^{Ala}$ using different isoforms of *E. coli* $tRNA^{Ala}$. The reactions were analyzed by matrix-assisted laser desorption/ionization time-of-flight mass spectrometry (MALDI-TOF-MS). Reactions contained the indicated $tRNA^{Ala}$ (6 µM), BhaA (20 µM), $BhaB_C^{Ala}$ (3 µM), ATP (5 mM), alanine (8 mM), and *E. coli* AlaRS (3 µM) in a solution of HEPES (100 mM, pH 7.5), Mg^{2+} (10 mM), and KCl (10 mM) in a 25-µL reaction volume. The asterisk (*) represents deamination of the indicated peptide species, a result specific to reflector positive mode in MALDI-TOF-MS.[18,19] Sequence of His_6-BhaA:

GSSHHHHHHSSGLVPRGSHMADKVTPEEELDLELEIEDLDDIDFDLEEIEDKVAPLAL

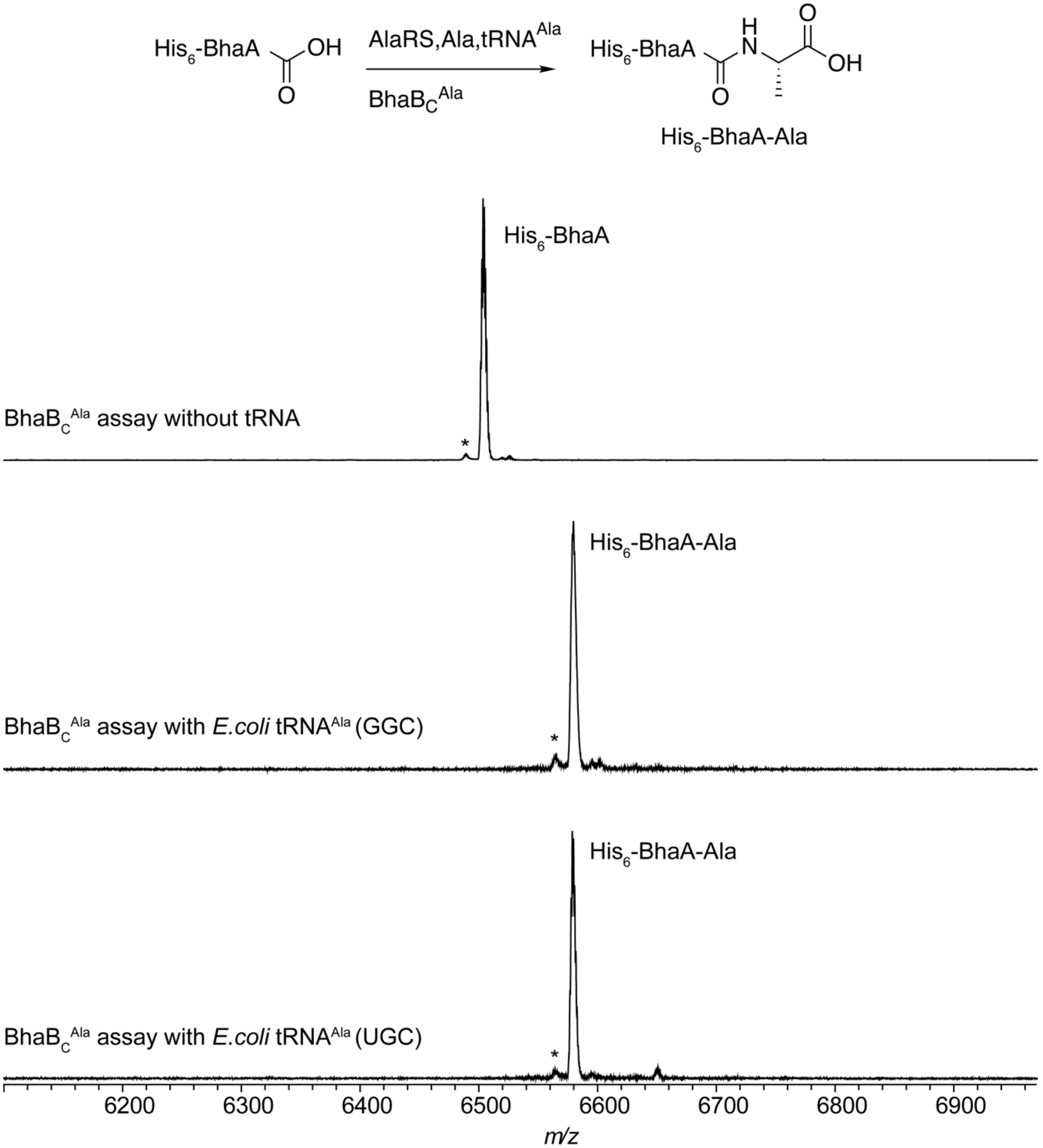

Figure S2. The NaIO₄-RNA extension assay. A) Schematic of the assay used to determine the efficiency of the flexizyme-catalyzed tRNA aminoacylation reactions.[9-11] tRNAs bearing free 3′-ends were selectively oxidized by $NaIO_4$, preventing templated extension by DNA Polymerase I Klenow fragment (3′→5′ exo-).[9-11] In contrast, aminoacylated tRNAs were protected from oxidation and permit extension, resulting in a distinguishable size difference upon PAGE analysis. Cy3 was used for visualization using a fluorescence imager. B) PAGE analysis with a fluorescence imager (Cy3 channel) of the method described in panel A using Ala-$tRNA^{Ala}$(UGC) of *E. coli*. Detailed experimental conditions are in Materials and Methods. In this experiment (R1), the aminoacylation efficiency is estimated at 35%. The sequence of the primer is provided in Table S5. C) Calibration lanes generated by mixing species a and b (panel A, Schemes 2 and 3) at the indicated [a/(a+b)] percentages.

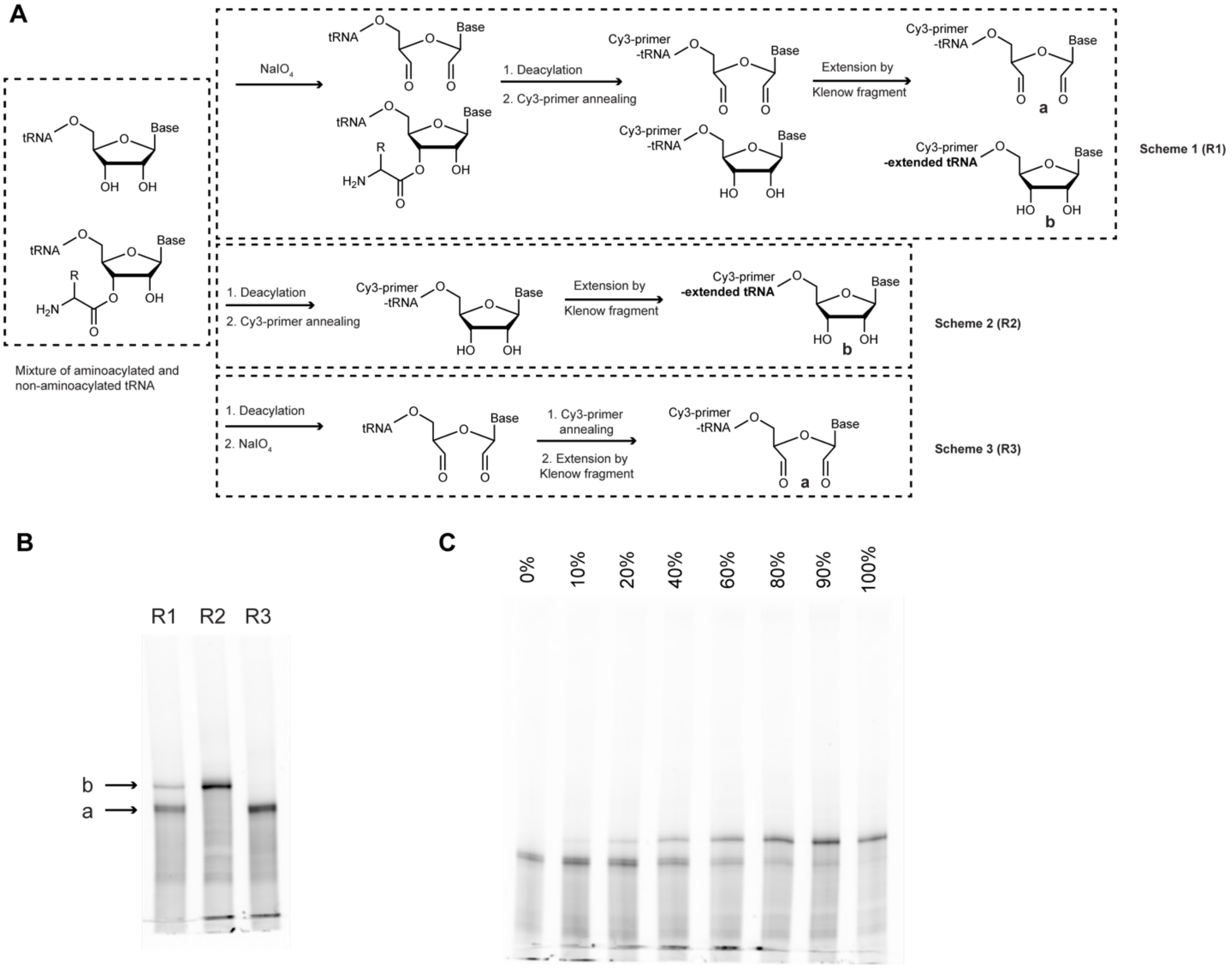

Figure S3. Examination of Δ19BhaA as a substrate of $BhaB_C^{Ala}$. Extracted ion chromatograms (EIC) of the liquid chromatography-mass spectrometry (LC-MS) trace of the $BhaB_C^{Ala}$ reaction using Δ19BhaA substrate peptide (sequence shown on top). The aminoacyl-tRNA complex of Ala and *E. coli* $tRNA^{Ala}$ (UGC) was generated by AlaRS. The black trace is the EIC of the substrate Δ19BhaA. The red trace is the EIC of the product Δ19BhaA-Ala.

Δ19BhaA: `LDDIDFDLEEIEDKVAPLAL`

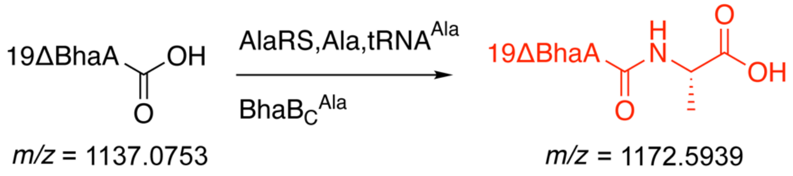

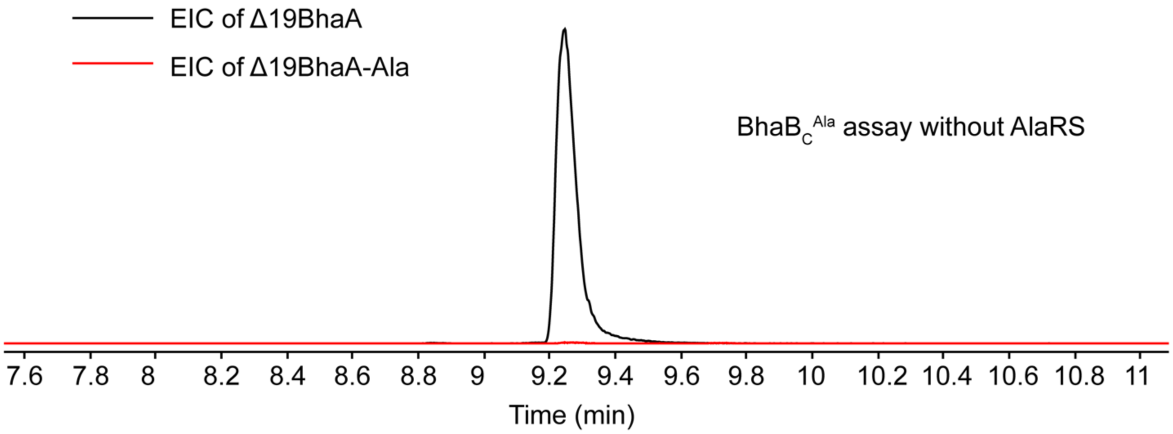

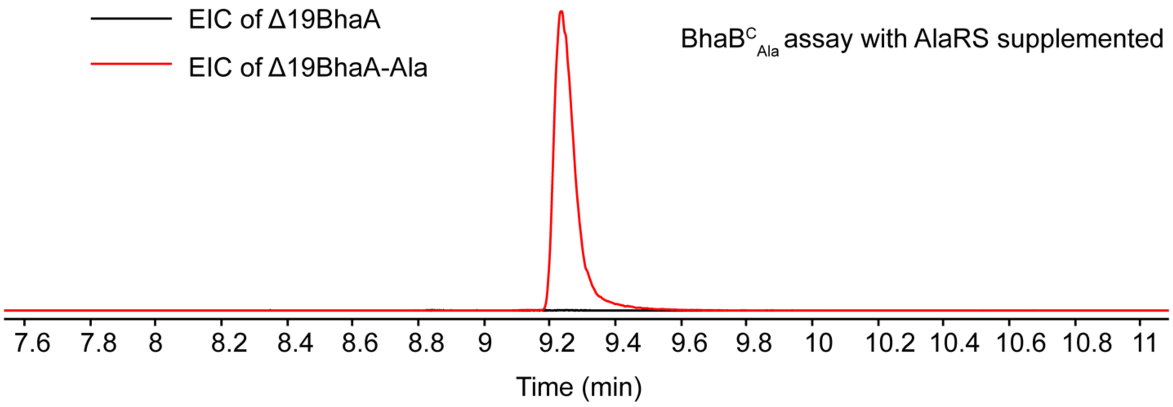

Figure S4. EICs of Δ19BhaA modified by $BhaB_C^{Ala}$ when reacting *E. coli* $tRNA^{Ala}$ (UGC) charged with Ala using flexizyme. Each panel represents $BhaB_C^{Ala}$ reaction when utilizing a different amount of flexizyme-generated Ala-$tRNA^{Ala}$. The concentration of the substrate peptide (Δ19BhaA) was 1.8 µM. The estimated concentration of Ala-$tRNA^{Ala}$ in each panel using the method in Fig. S1 was as follows: A) 37 µM, B) 19 µM, C) 9.3 µM, D) 4.7 µM, and E) 2.3 µM. The estimated reaction efficiency was A) 99%, B) 97%, C), D) 98%, and E) 72%. The black trace is the EIC of the substrate Δ19BhaA. The red trace is the EIC of the product Δ19BhaA-Ala. F) The mass spectrum of the substrate and the product. The calculated error can be found in Table S6.

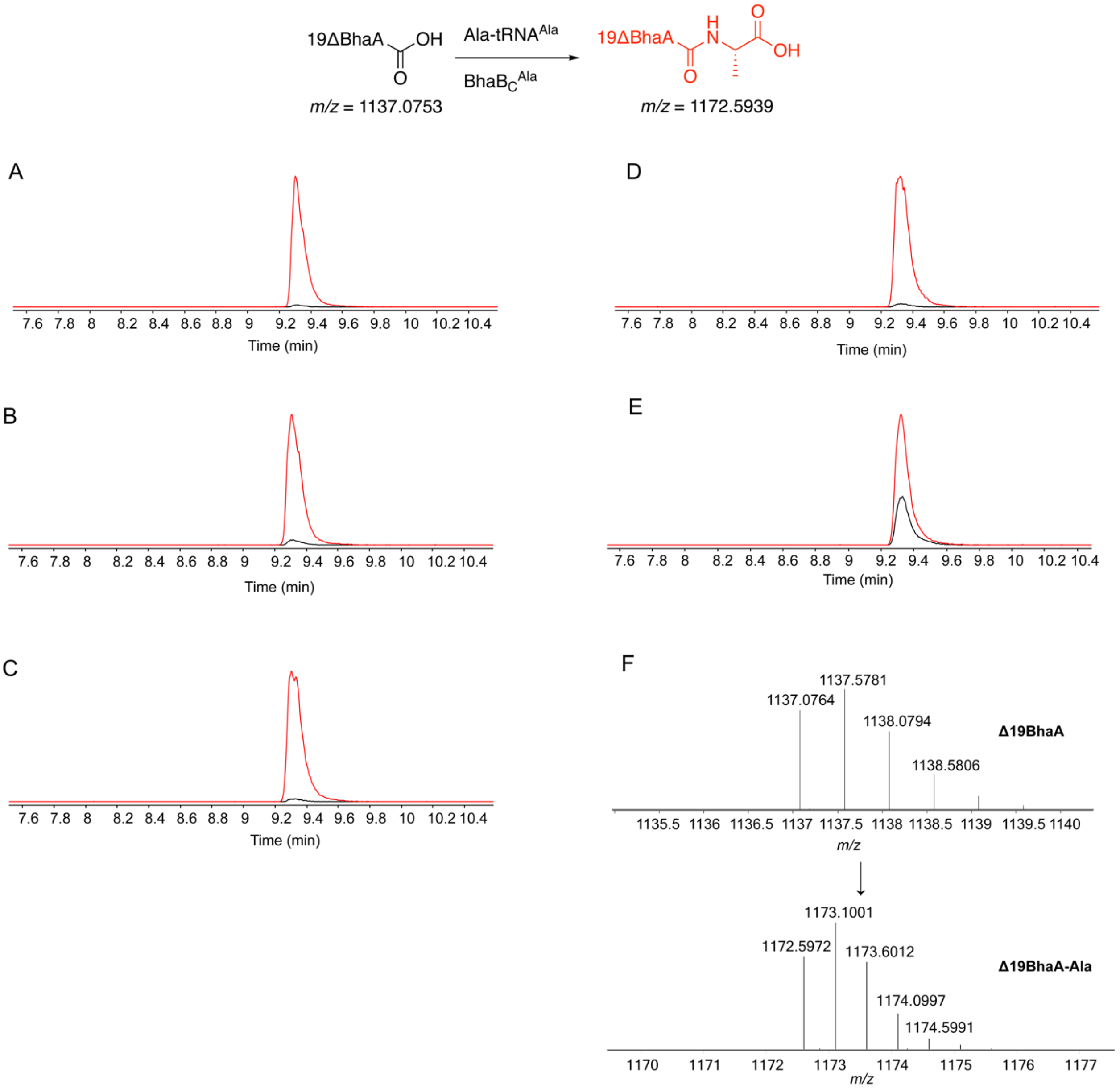

Figure S5. PAGE analysis of the $NaIO_4$-RNA extension assays (Figure S2) on flexizyme-catalyzed acylation products of *E. coli* $tRNA^{Ala}$(UGC) with various amino acids. The upper band represents the product formed from aminoacylated-tRNA (species b, Figure S2) and the lower band represents the free tRNA (species a, Figure S2). Lane 1: N-methyl-L-Ala (estimated aminoacylation efficiency: 29%); Lane 2: D-Ala (30%), Lane 3: L-Glu (16%), Lane 4: L-Gln (11%), Lane 5: L-Trp (23%), Lane 6: 2-aminoisobutyric acid (Aib) (26%), Lane 7: β-Ala (17%), Lane 8: Gly (40%), Lane 9: Phe (28%). The result with L-Ala is shown in Figure S2B.

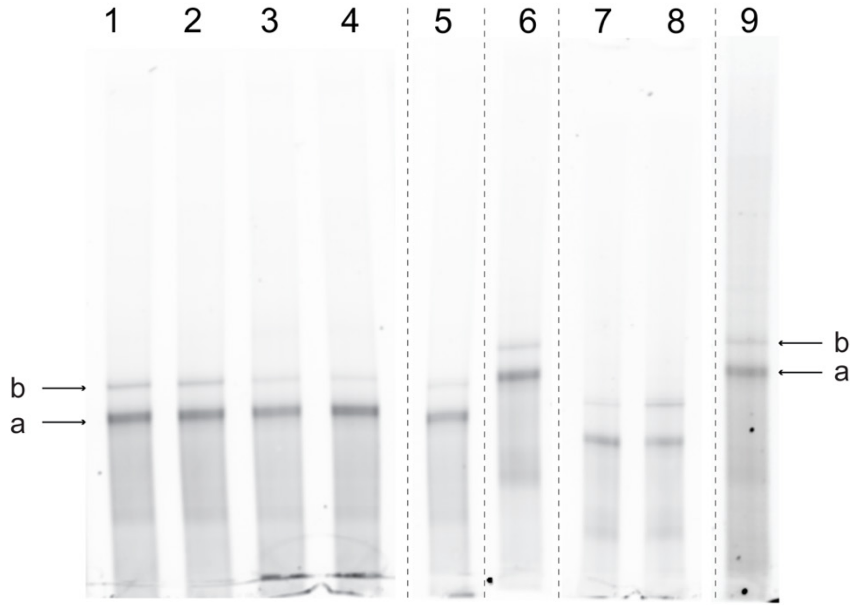

Acids	Lane	Estimated aminoacylation efficiency (%)
N-methyl-L-Ala	1	29%
D-Ala	2	30%
L-Glu	3	16%
L-Gln	4	11%
L-Trp	5	23%
2-Aminoisobutyric acid (Aib)	6	26%
β-Ala	7	17%
Gly	8	40%
Phe	9	28%
L-Ala	(Figure S2B)	35%

Figure S6. EICs of Δ19BhaA modified by $BhaB_C^{Ala}$ in the presence of *E. coli* $tRNA^{Ala}$ (UGC) charged with Gly. Each panel represents the $BhaB_C^{Ala}$ reaction when utilizing different amounts of Gly-$tRNA^{Ala}$. The concentration of the substrate peptide (Δ19BhaA) was 1.8 µM. The estimated concentration of Gly-$tRNA^{Ala}$ in each panel was as follows: A) 27 µM, B) 14 µM, C) 6.8 µM, D) 3.4 µM, and E) 1.7 µM. The estimated reaction efficiency is as follows: A) 99%, B) 99%, C) 95%, D) 63%%, and E) 32%. The black trace is the EIC of the substrate Δ19BhaA. The red trace is the EIC of the product Δ19BhaA-Gly. F) The ESI mass spectrum of the product. The calculated error comparing calculated and observed masses is shown in Table S6.

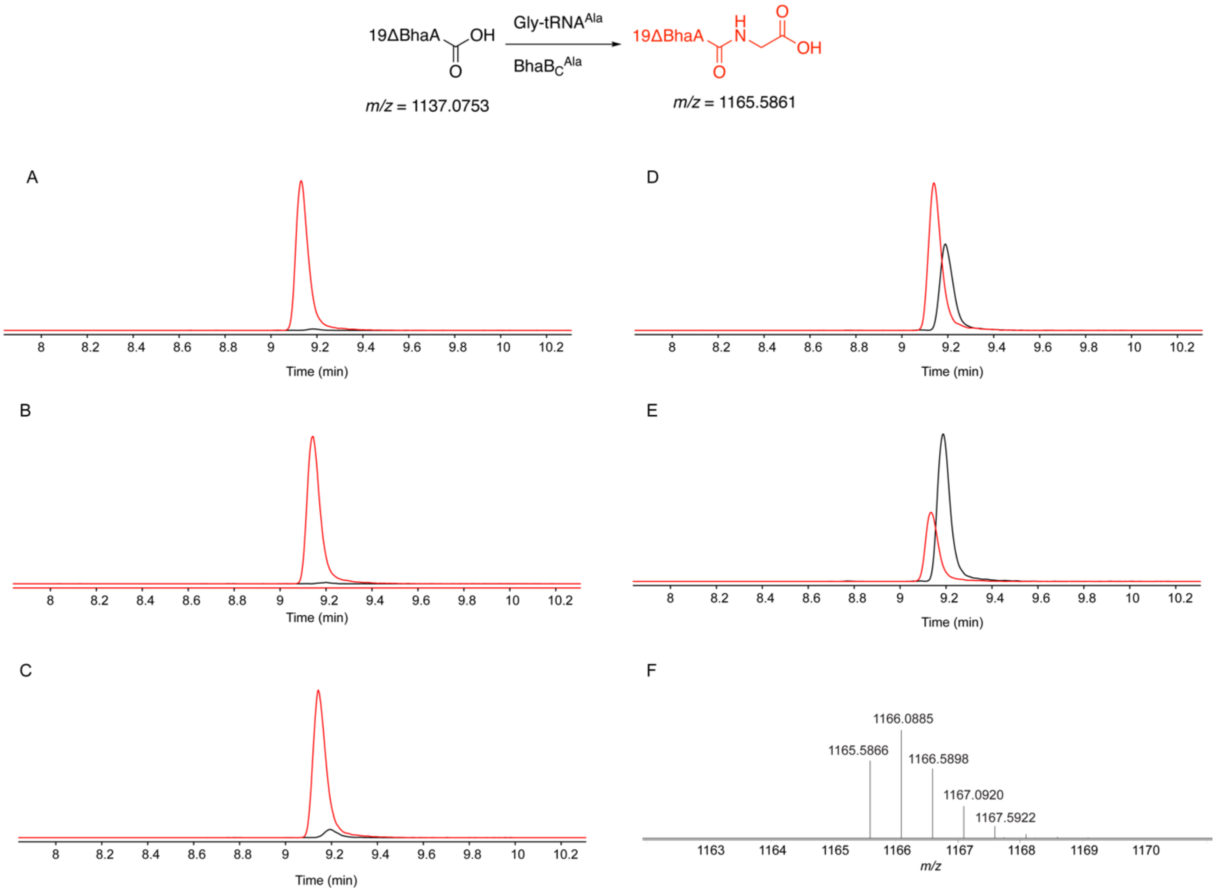

Figure S7. EICs of Δ19BhaA modified by $BhaB_C^{Ala}$ in the presence of *E. coli* $tRNA^{Ala}$ (UGC) charged with Gln. Each panel represents a $BhaB_C^{Ala}$ reaction utilizing different amounts of Gln-$tRNA^{Ala}$. The concentration of the substrate peptide (Δ19BhaA) was 1.8 µM. The estimated concentration of Gln-$tRNA^{Ala}$ in each panel was as follows: A) 15 µM, B) 7.7 µM, C) 3.8 µM, D) 1.9 µM, and E) 0.96 µM. The estimated reaction efficiency is as follows: A) 14%, B) 9.1%, C) 6.4%, D) 5.0%, and E) 2.9%. The black trace is the EIC of the substrate Δ19BhaA. The red trace is the EIC of the product Δ19BhaA-Gln. F) The mass spectrum of the product. The calculated error comparing calculated and observed masses is shown in Table S6.

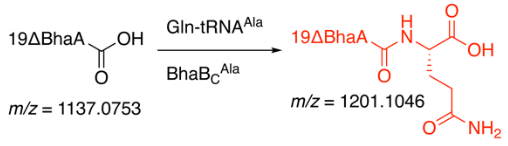

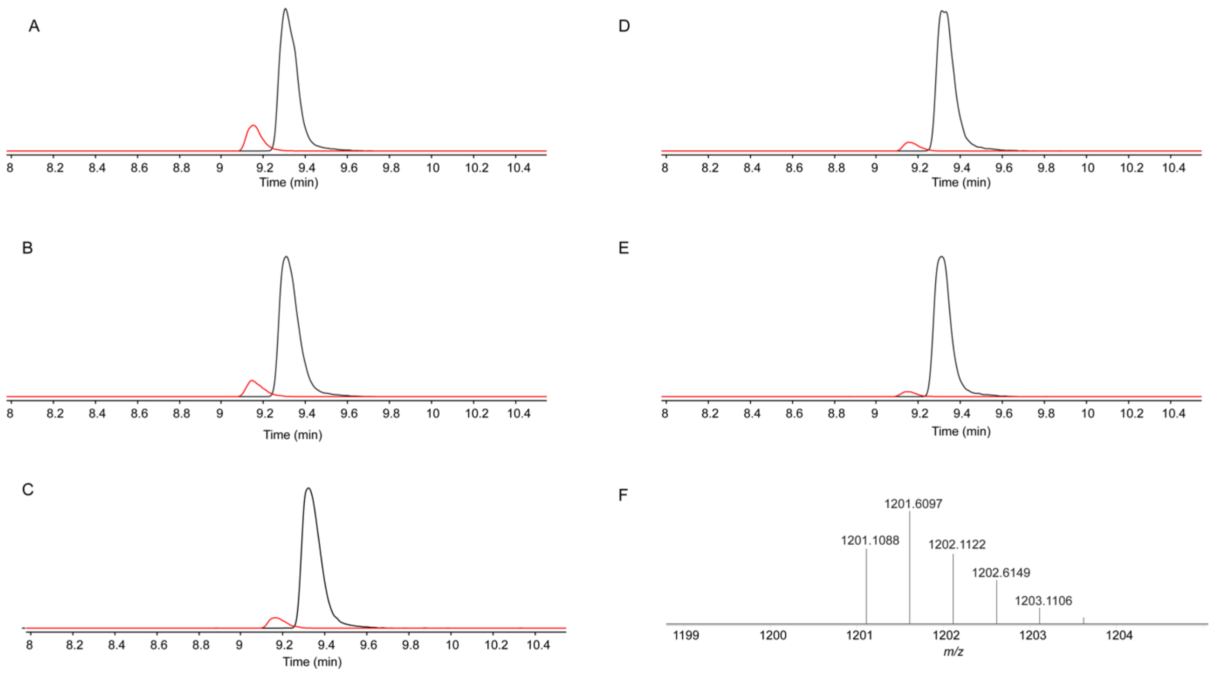

Figure S8. EICs of Δ19BhaA modified by $BhaB_C^{Ala}$ in the presence of *E. coli* $tRNA^{Ala}$ (UGC) charged with Glu. Each panel represents a $BhaB_C^{Ala}$ reaction when utilizing different amounts of Glu-$tRNA^{Ala}$. The concentration of the substrate peptide **(**Δ19BhaA**)** was 1.8 µM. The estimated concentration of Glu-$tRNA^{Ala}$ in each panel was as follows: A) 22 µM, B) 11 µM, C) 5.5 µM, D) 2.8 µM, and E) 1.4 µM. The estimated reaction efficiency is as follows: A) 3.6%, B) 2.8%, C) 2.4%, D) 1.9%, and E) 1.3%. The black trace is the EIC of the substrate Δ19BhaA. The red trace is the EIC of the product Δ19BhaA-Glu. F) The mass spectrum of the product. The calculated error comparing calculated and observed masses is shown in Table S6.

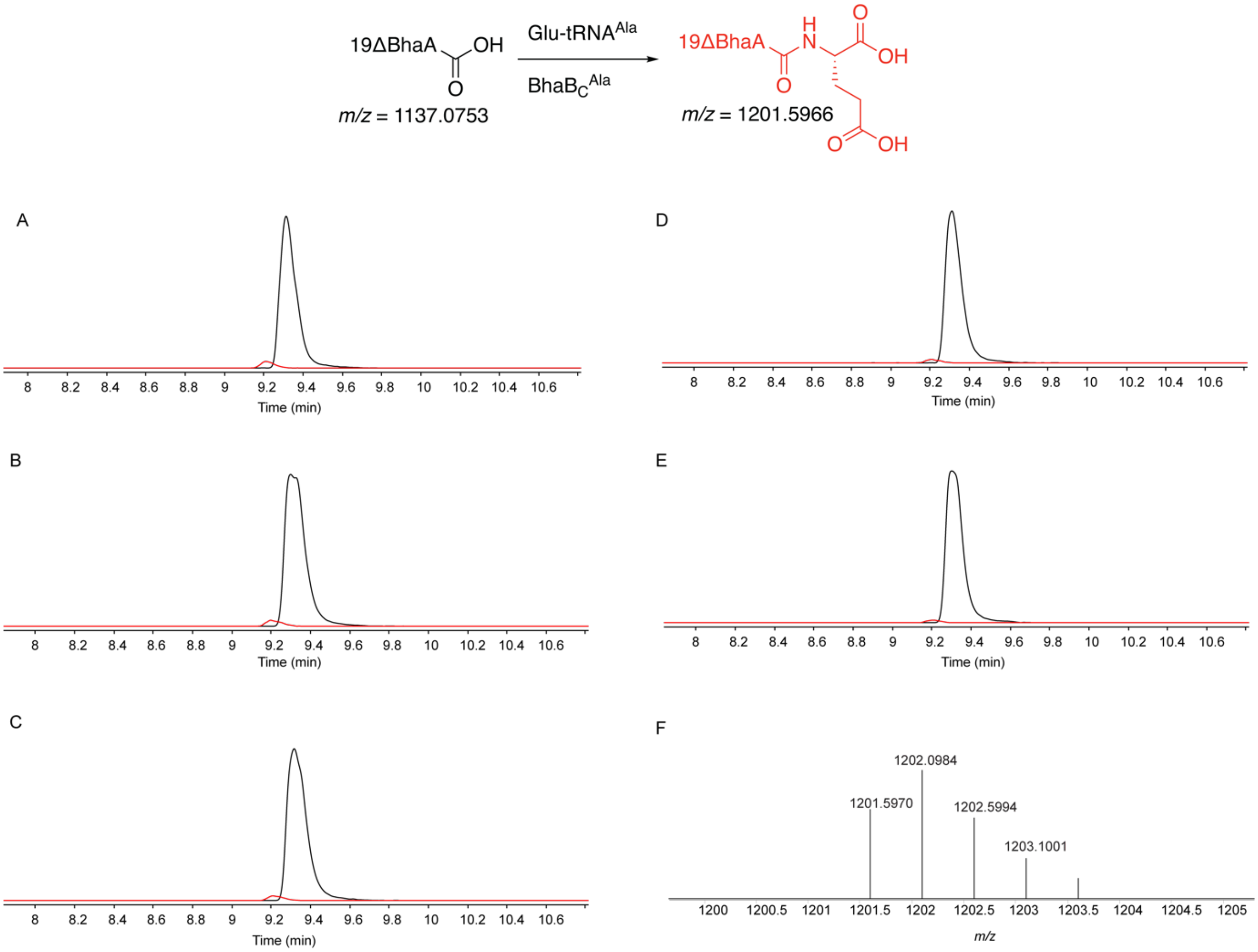

Figure S9. EICs of Δ19BhaA modified by $BhaB_C^{Ala}$ in the presence of *E. coli* $tRNA^{Ala}$ (UGC) charged with Phe. Each panel represents a $BhaB_C^{Ala}$ reaction when utilizing different amounts of Phe-$tRNA^{Ala}$. The concentration of the substrate peptide **(**Δ19BhaA) was 1.8 µM. The estimated concentration of Phe-$tRNA^{Ala}$ in each panel was as follows: A) 38 µM , B) 19 µM, C), 9.4 µM, D) 4.7 µM , and E) 2.4 µM. The estimated reaction efficiency is as follows: A) 33%, B) 38% , C) 33%, D) 24%, and E) 16%. The black trace is the EIC of the substrate Δ19BhaA. The red trace is the EIC of the product Δ19BhaA-Phe. F) The mass spectrum of the product. The calculated error comparing calculated and observed masses is shown in Table S6.

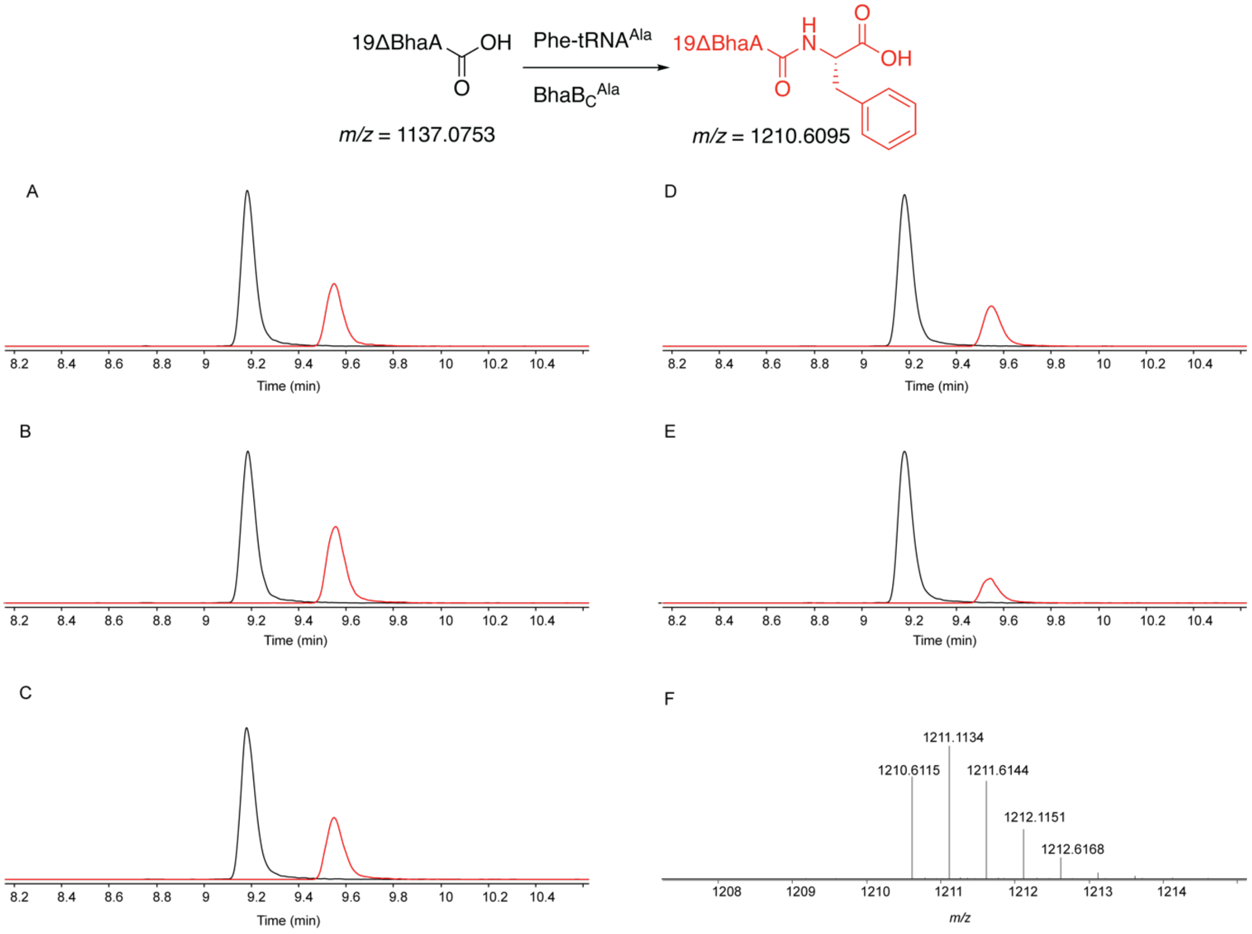

Figure S10. EICs of Δ19BhaA modified by $BhaB_C^{Ala}$ in the presence of *E. coli* $tRNA^{Ala}$ (UGC) charged with Trp. Each panel represents a $BhaB_C^{Ala}$ reaction when utilizing different amounts of Trp-$tRNA^{Ala}$. The concentration of the substrate peptide (Δ19BhaA) was 1.8 µM. The estimated concentration of Trp-$tRNA^{Ala}$ in each panel was as follows: A) 31 µM, B) 16 µM, C) 7.9 µM, D) 3.9 µM, and E) 2.0 µM. The estimated reaction efficiency is as follows: A) 3%, B) 2%, C) 2.3%, D) 1.8%, and E) 0.7%. The black trace is the EIC of the substrate Δ19BhaA. The red trace is the EIC of the product Δ19BhaA-Trp. F) The mass spectrum of the product. The calculated error comparing calculated and observed masses is shown in Table S6.

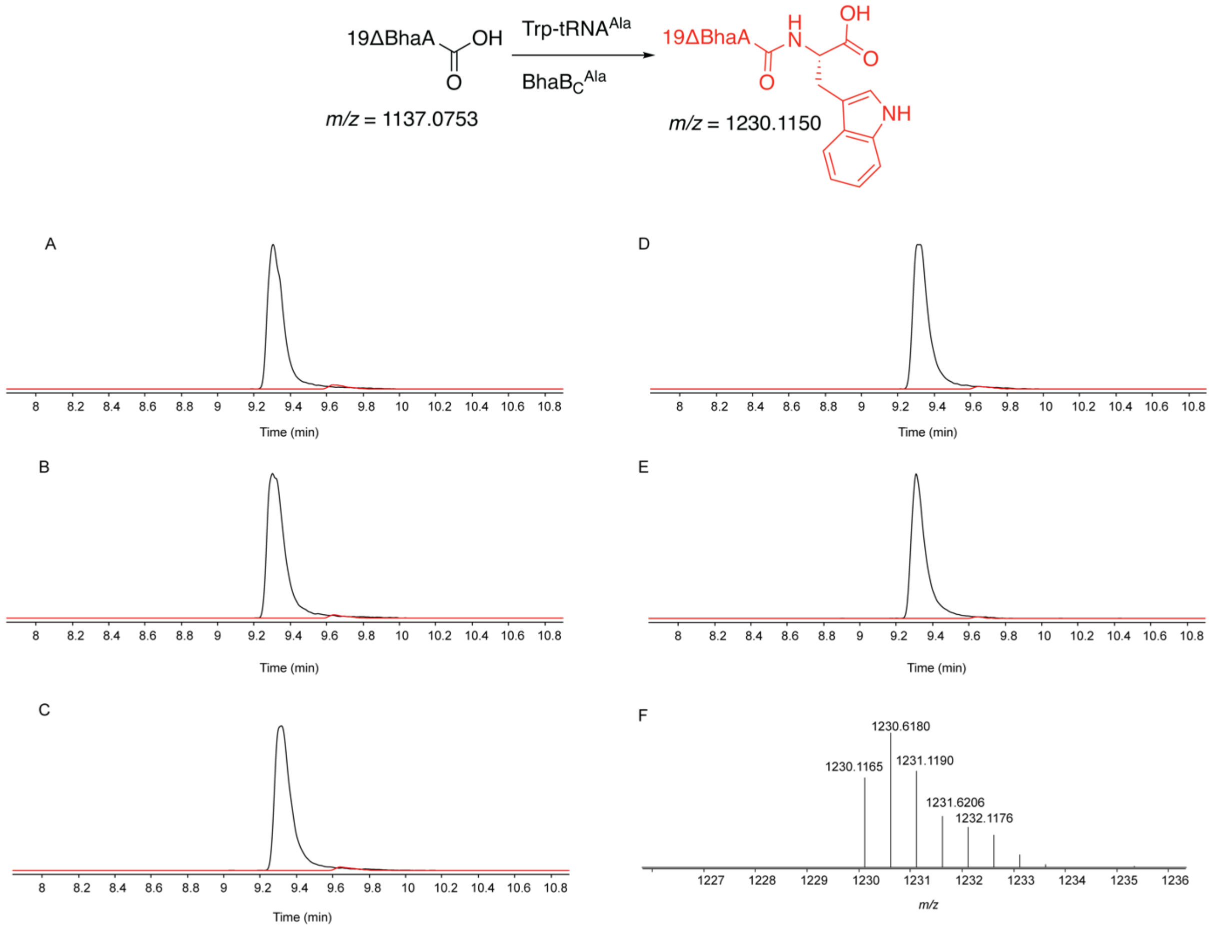

Figure S11. EICs of Δ19BhaA modified by $BhaB_C^{Ala}$ in the presence of *E. coli* $tRNA^{Ala}$ (UGC) charged with β-Ala. Each panel represents a $BhaB_C^{Ala}$ reaction when utilizing different amounts of (β-Ala)-$tRNA^{Ala}$. The concentration of the substrate peptide **(**Δ19BhaA) was 1.8 µM. The estimated concentration of (β-Ala)-$tRNA^{Ala}$ in each panel was as follows: A) 12 µM, B) 6 µM, C) 3 µM, D) 1.5 µM, and E) 0.74 µM. The estimated reaction efficiency is as follows: A) 86%, B) 61%, C) 39%, D) 20%, and E) 10%. The black trace is the EIC of the substrate Δ19BhaA. The red trace is the EIC of the product Δ19BhaA-(β-Ala). F) The mass spectrum of the product. The calculated error comparing calculated and observed masses is shown in Table S6.

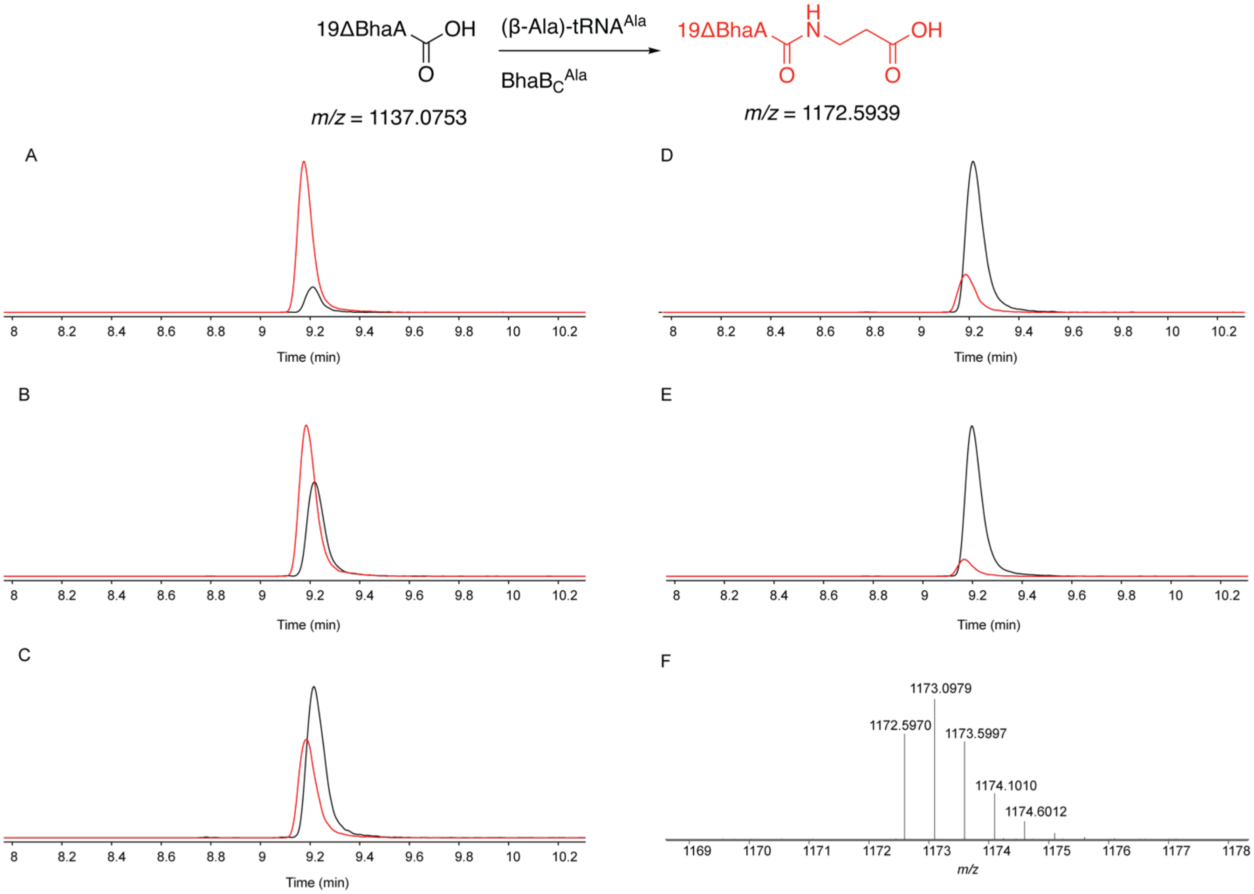

Figure S12. EICs of Δ19BhaA modified by $BhaB_C^{Ala}$ in the presence of *E. coli* $tRNA^{Ala}$ (UGC) charged with D-Ala. Each panel represents a $BhaB_C^{Ala}$ reaction when utilizing different amounts of (D-Ala)-$tRNA^{Ala}$. The concentration of the substrate peptide (Δ19BhaA) was 1.8 µM. The estimated concentration of (D-Ala)-$tRNA^{Ala}$ in each panel was as follows: A) 41 µM, B) 21 µM, C) 10 µM, D) 5.1 µM, and E) 2.6 µM. The estimated reaction efficiency is as follows: A) 8.0%, B) 5.7%, C) 3.9%, D) 2.8%, and E) 1.8%. The black trace is the EIC of the substrate Δ19BhaA. The red trace is the EIC of the product Δ19BhaA-(D-Ala). F) The mass spectrum of the product. The calculated error comparing calculated and observed masses is shown in Table S6.

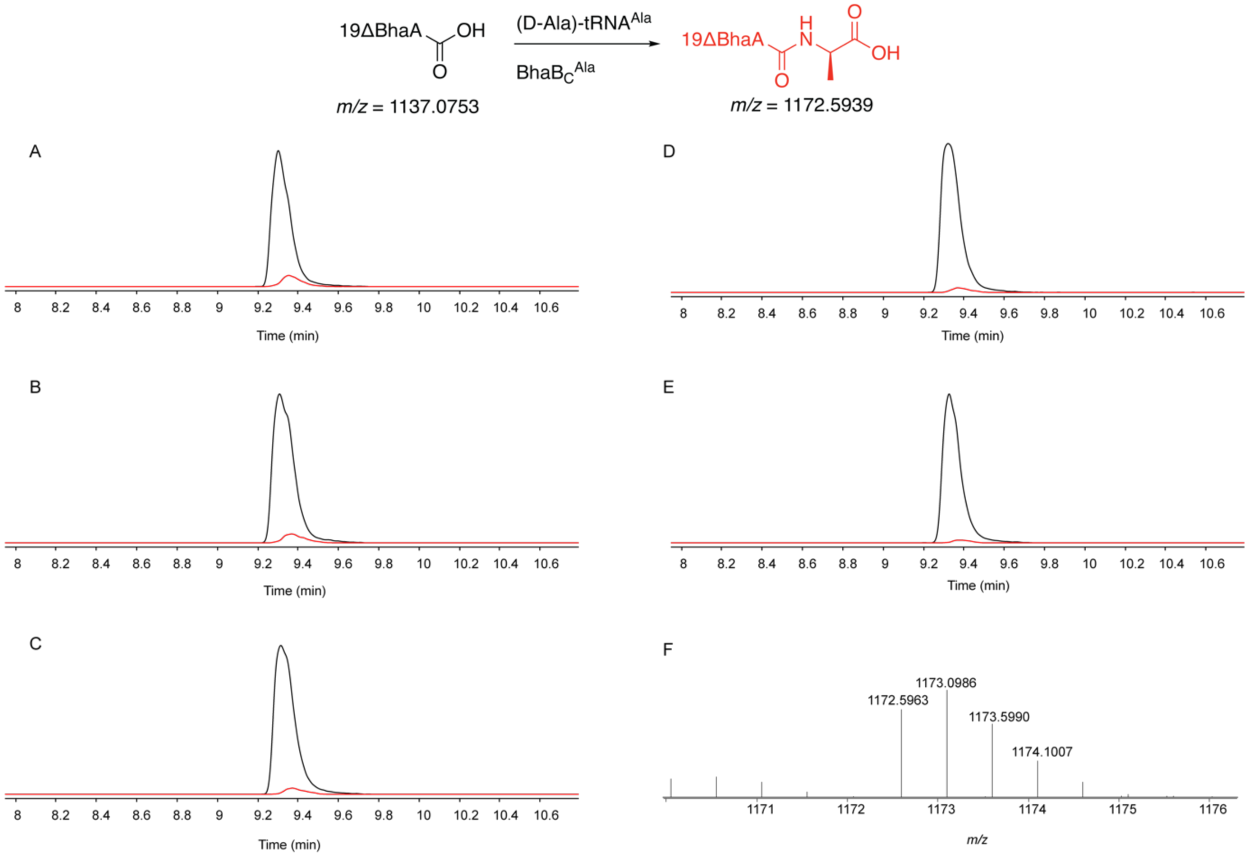

Figure S13. EICs of Δ19BhaA modified by $BhaB_C^{Ala}$ in the presence of *E. coli* $tRNA^{Ala}$ (UGC) charged with 2-aminoisobutyric acid (Aib). Each panel represents a $BhaB_C^{Ala}$ reaction when utilizing different amounts of Aib-$tRNA^{Ala}$. The concentration of the substrate peptide **(**Δ19BhaA) was 1.8 µM. The estimated concentration of Aib-$tRNA^{Ala}$ in each panel was as follows: A) 17 µM, B), 8.7 µM C) 4.3 µM, D) 2.2 µM, and E) 1.1 µM. The product was not observed in any conditions. The black trace is the EIC of the substrate Δ19BhaA. The red trace is the EIC of the product Δ19BhaA-Aib.

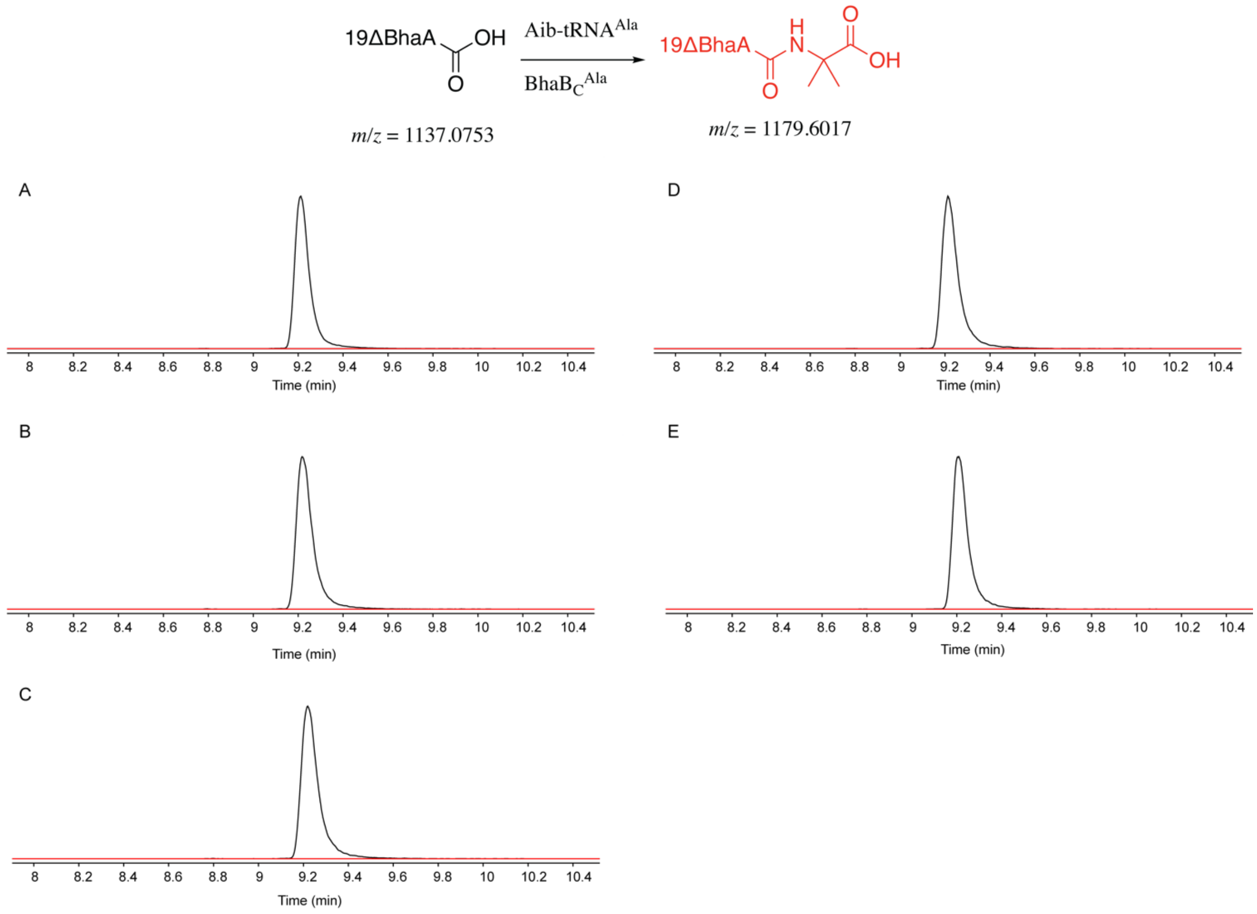

Figure S14. EICs of Δ19BhaA modified by $BhaB_C^{Ala}$ in the presence of *E. coli* $tRNA^{Ala}$ (UGC) charged with N-Me-Ala. Each panel represents a $BhaB_C^{Ala}$ reaction when utilizing different amounts of (N-Me-Ala)-$tRNA^{Ala}$. The concentration of the substrate peptide (Δ19BhaA) was 1.8 µM. The estimated concentration of (N-Me-Ala)-$tRNA^{Ala}$ in each panel was as follows: A) 40 µM, B) 20 µM, C) 10 µM, D) 5 µM, and E) 2.5 µM. The product was not observed in any conditions. The black trace is the EIC of the substrate Δ19BhaA. The red trace is the EIC of the product Δ19BhaA-(N-Me-Ala).

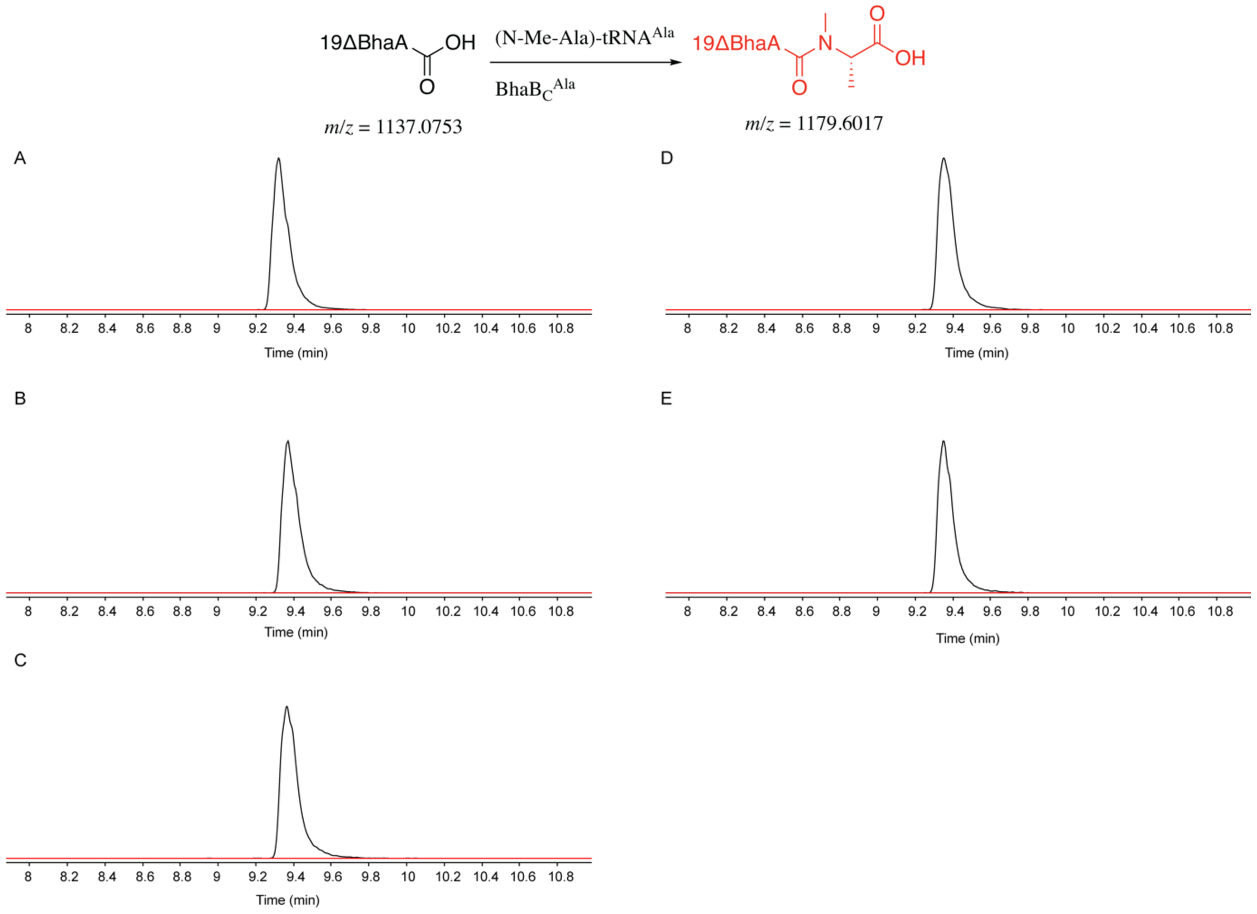

Figure S15. EICs of Δ19BhaA modified by $BhaB_C^{Ala}$ in the presence of *E. coli* $tRNA^{Ala}$ (UGC) charged with Lac. The concentration of the substrate peptide **(**Δ19BhaA) was 1.8 µM. Panels A–E show reactions with decreasing concentrations of Lac-$tRNA^{Ala}$, beginning with the highest concentration in Panel A and followed by two-fold serial dilutions through Panel E. The estimated reaction efficiency is as follows: A) 21%, B) 15%, C) 8.8%, D) 5.1%, and E) 2%. The black trace is the EIC of the substrate Δ19BhaA. The red trace is the EIC of the product Δ19BhaA-Lac. F) The mass spectrum of the product. The calculated error comparing calculated and observed masses is shown in Table S6.

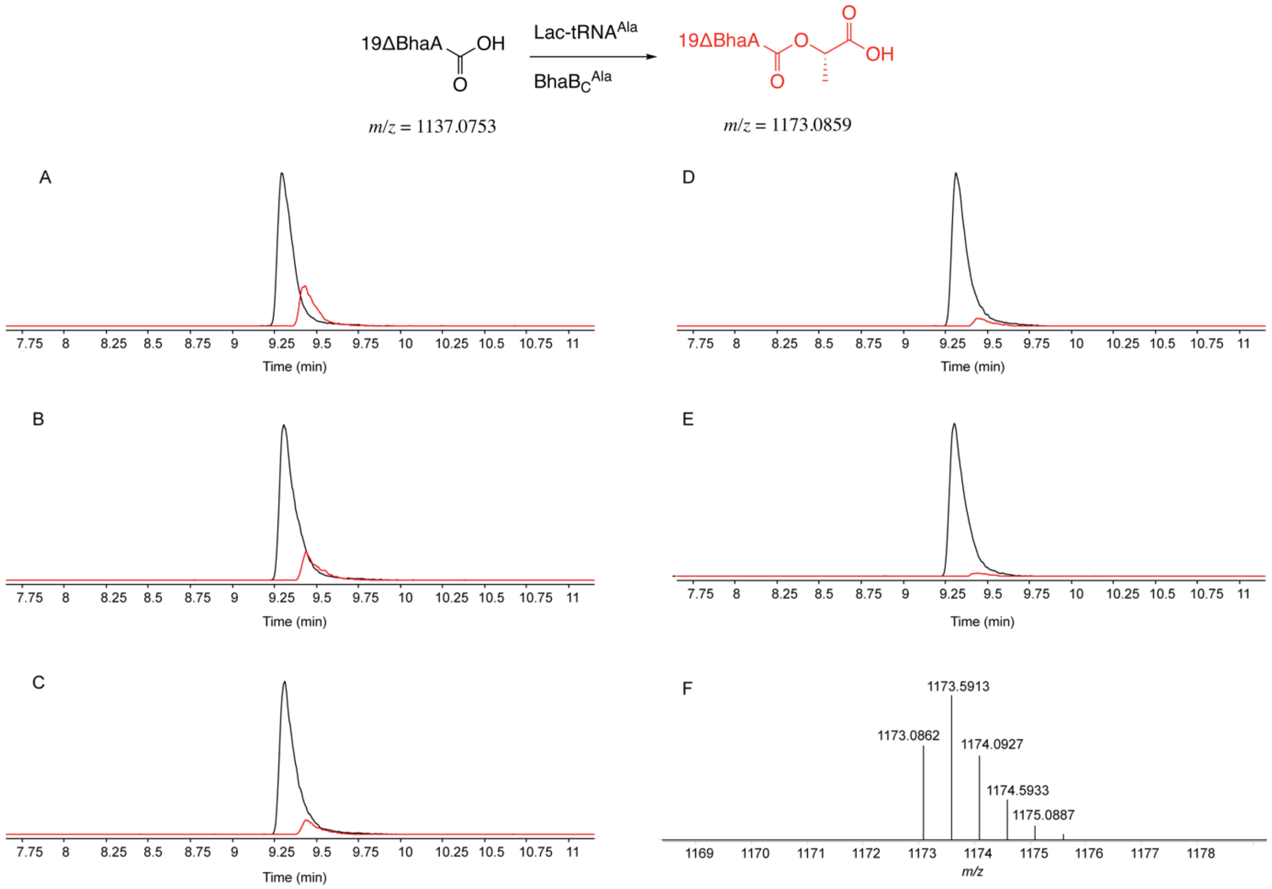

Figure S16. EICs of Δ19BhaA modified by $BhaB_C^{Ala}$ in the presence of *E. coli* $tRNA^{Ala}$ (UGC) charged with thioglycolic acid (ThioGly). The concentration of the substrate peptide **(**Δ19BhaA) was 1.8 μM. Panels A–E show reactions with decreasing concentrations of ThioGly-$tRNA^{Ala}$, beginning with the highest concentration in Panel A and followed by two-fold serial dilutions through Panel E. The estimated reaction efficiency is as follows: A) 3.9%, B) 1.9%, C) 0.80%, D) 0.42%, and E) 0.25%. The black trace is the EIC of the substrate Δ19BhaA. The blue trace is the EIC of the product Δ19BhaA-ThioGly. F) The mass spectrum of Δ19BhaA-ThioGly. The calculated error comparing calculated and observed masses is shown in Table S6.

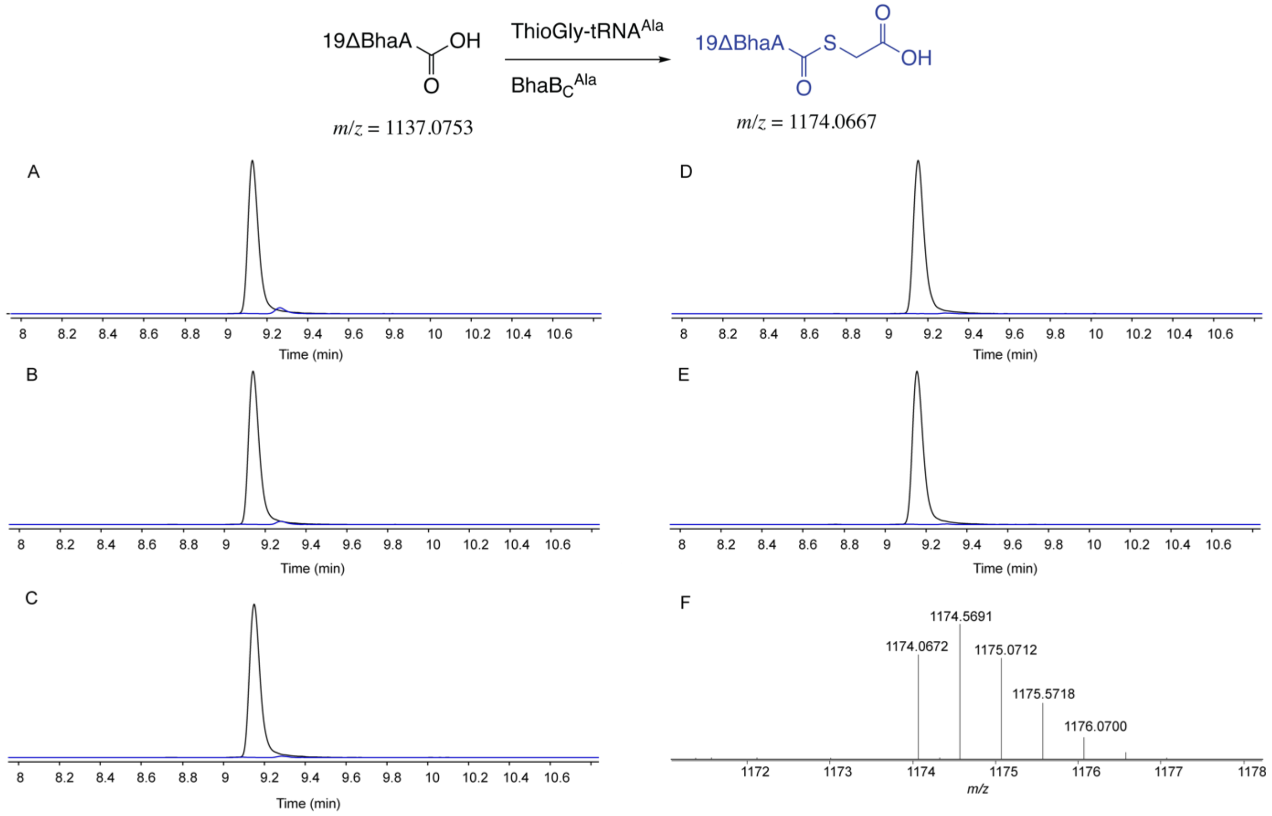

Figure S17. Native chemical ligation (NCL) of the peptide-thioester generated by $BhaB_C^{Ala}$ and ThioGly (Figure S16). EICs and mass spectra of products from $BhaB_C^{Ala}$-catalyzed reactions using ThioGly-$tRNA^{Ala}$ in the presence or absence of cysteamine. Cysteamine reacts with the thioester product of the $BhaB_C^{Ala}$ reaction via NCL.[20] The black trace corresponds to the EIC of the substrate (Δ19BhaA), the blue trace corresponds to the thioester product (Δ19BhaA–ThioGly), and the red trace corresponds to the NCL product (Δ19BhaA–cysteamine). The calculated error comparing calculated and observed masses is shown in Table S6.

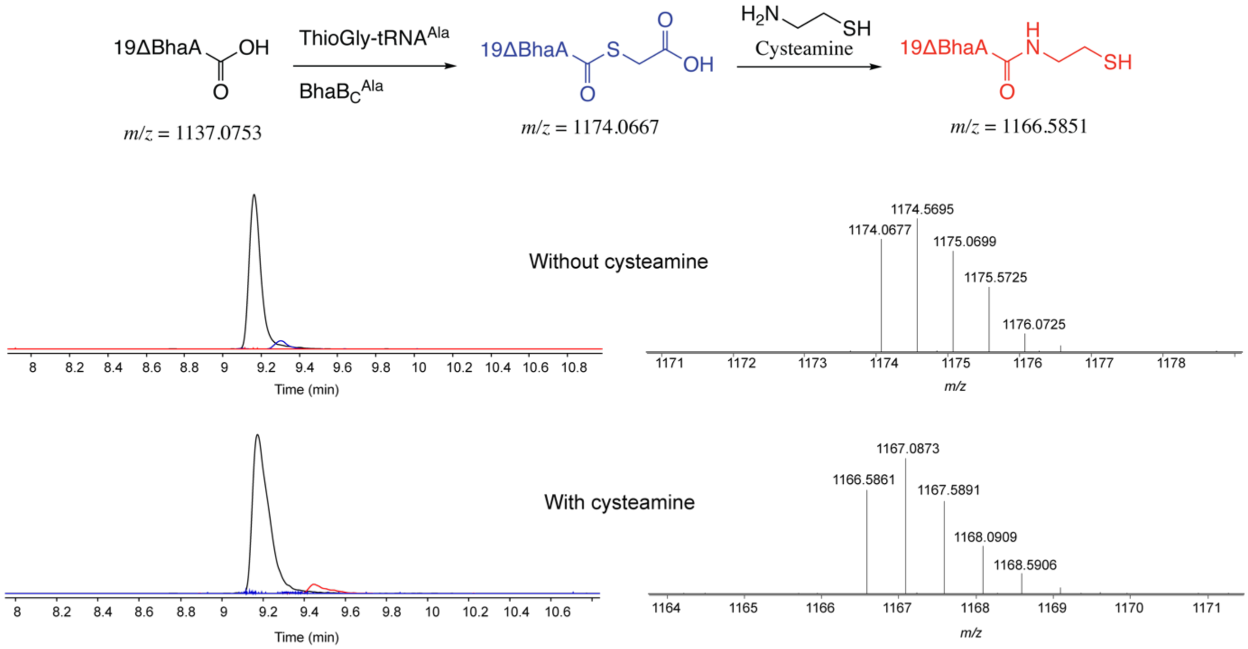

Figure S18. Δ19BhaA modified by $BhaB_C^{Ala}$ in the presence of *E. coli* $tRNA^{Gly}$(GCC) charged with Gly by *E. coli* GlyRS. Gly-$tRNA^{Gly}$ was partially purified after GlyRS assay using the Monarch Spin RNA Cleanup Kit before being subjected to the $NaIO_4$-RNA extension assay and the $BhaB_C^{Ala}$ assay. A) PAGE result of the $NaIO_4$-RNA extension assay (refer to Figure S2). The estimated amount of aminoacylated tRNA (species b) compared to the total amount of RNA (species a and b) is 36%. B) EIC collected after the $BhaB_C^{Ala}$ reaction using 1.6 µM Δ19BhaA and 8.8 µM Gly-$tRNA^{Gly}$. C) The predicted clover leaf structures of *E. coli* $tRNA^{Gly}$ (GCC) using tRNAScanSE[21] and Forna.[22] D) Sequence alignments of *E. coli* $tRNA^{Ala}$ (UGC) and $tRNA^{Gly}$ (GCC).[23-25] The tRNA is colored by structural region: orange (acceptor stem), yellow (D-arm), green (anticodon arm), black (variable arm), and blue (T-stem).

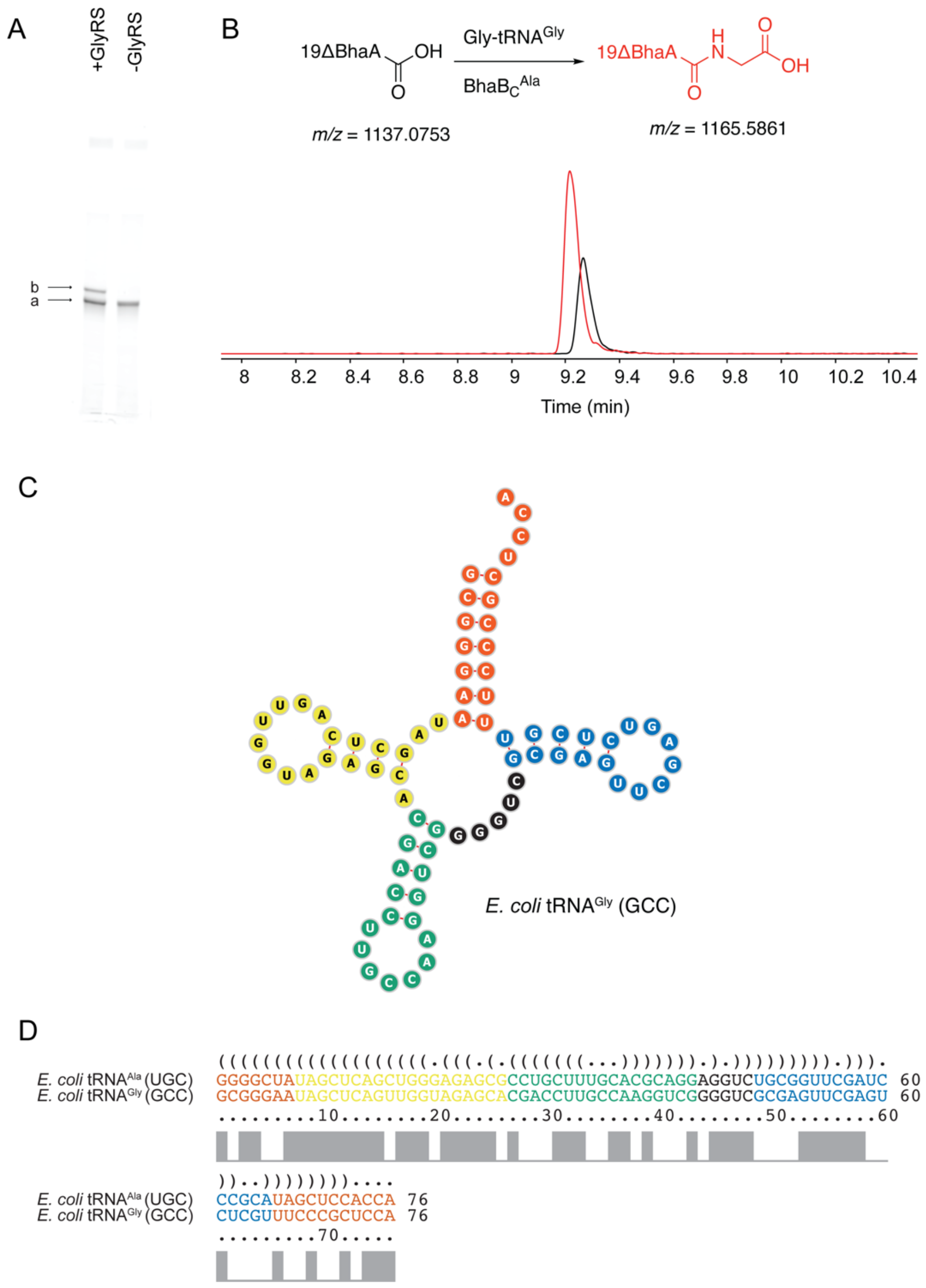

Figure S19. Predicted structures of examined tRNAs charged to Ala. Their sequences are provided in Table S4. The clover leaf structures are shown for *E. coli* $tRNA^{Ala}$(UGC), *E. coli* $tRNA^{Trp}$, *T. bispora* $tRNA^{Glu}$ (CUC) and *P. syringae* $tRNA^{Cys}$ (GCA). The tRNA is colored by structural region: orange (acceptor stem), yellow (D-arm), green (anticodon arm), black (variable arm), and blue (T-stem). Sequence alignments comparing these tRNAs can be found in Figure S20. The structures were generated using tRNAScanSE[21] and Forna.[22]

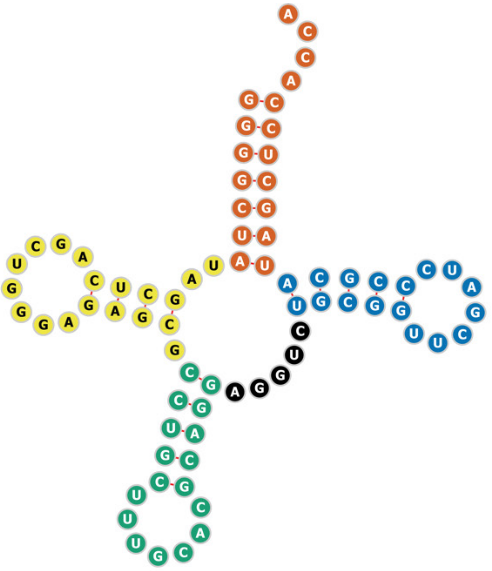

E. coli $tRNA^{Ala}$ (UGC)

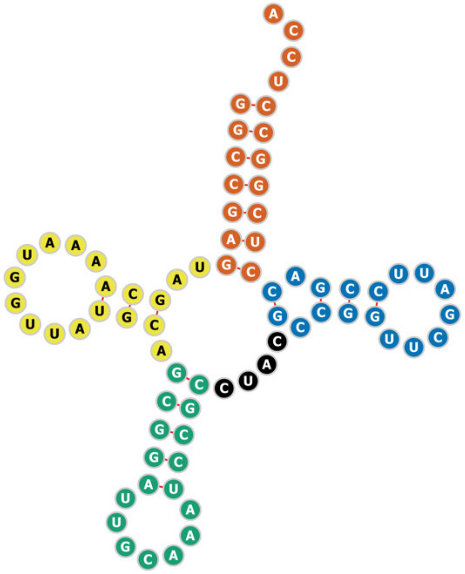

P. syringae $tRNA^{Cys}$ (GCA)

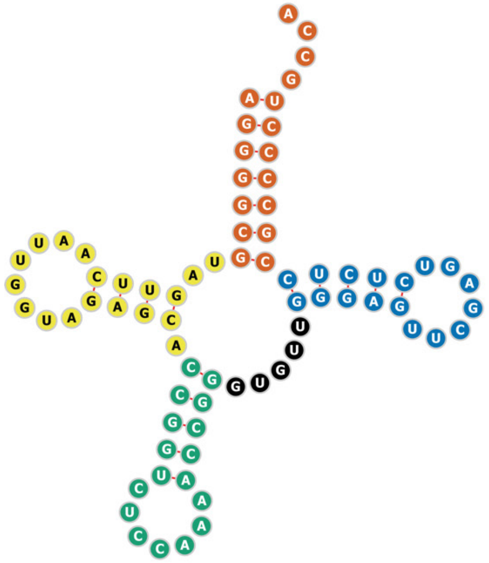

E. coli $tRNA^{Trp}$

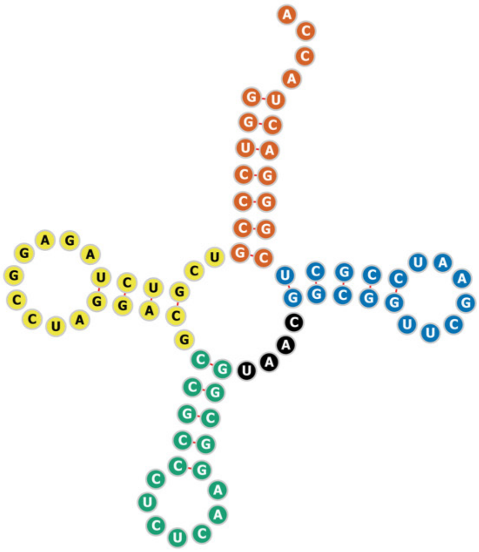

T. bispora $tRNA^{Glu}$ (CUC)

Figure S20. Sequence alignments of the tRNAs used to examine the Ala-tRNA specificity of $BhaB_C^{Ala}$. The boxed residues are the bases of the anticodon (green) and the discriminator bases (orange). Ec = *E. coli*, Ps = *P. syringae*, Tbi = *T. bispora*. The isoforms shown are $tRNA^{Cys}$(GCA), $tRNA^{Glu}$(CUC), and $tRNA^{Ala}$(UGC). The tRNA is colored by structural region: orange (acceptor stem), yellow (D-arm), green (anticodon arm), black (variable arm), and blue (T-stem) (see Figure S19). The alignments were performed using LocARNA.[23-25]

```
          (((((((..((((........)))).(((((.......))))).....(((((.......
EcTrp     AGGGGCGUAGUUCAAUUGGUAGAGCACCGGUCUCCAAAACCGGGUGUUGGGAGUUCGAGU 60
EcAla     GGGGCUAUAGCUCAGCUGGGAGAGCGCCUGCUUUGCACGCAGGAGGUCUGCGGUUCGAUC 60
          ........10........20........30........40........50........60

          ))))))))))))....
EcTrp     CUCUCCGCCCCUGCCA 76
EcAla     CCGCAUAGCUCCACCA 76
          ........70......
```

```
          (((((((..(((((......))))).(((((((...))))))).....(((((.......
PsCys     GGCCGAGUAGCA-AAAUGGUUAUGCAGCGGAUUGCAAAUCCGCCUA-CGCCGGUUCGAUU 58
EcAla     GGGGCUAUAGCUCAGCUGGGAGAGCGCCUGCUUUGCACGCAGGAGGUCUGCGGUUCGAUC 60
          ........10........20........30........40........50........60

          ))))))))))))....
PsCys     CCGACCUCGGCCUCCA 74
EcAla     CCGCAUAGCUCCACCA 76
          ........70......
```

```
          (((((((.(((((.........)))))(((((.......))))).....(((((......
TbiGlu    GGUCCCGUCGUCUAGAGGCCUAGGACGCCGCCCUCUCAAGGCGG-UAACGGCGGUUCGAA 59
EcAla     GGGGCUAUAGCUCAGCUG-GGAGAGCGCCUGCUUUGCACGCAGGAGGUCUGCGGUUCGAU 59
          ........10........20........30........40........50..........

          .))))))))))))....
TbiGlu    UCCGCUCGGGACUACCA 76
EcAla     CCCGCAUAGCUCCACCA 76
         60........70......
```

Figure S21. (A) PAGE analysis of the $NaIO_4$-RNA extension assay (Figure S2) with different tRNAs charged with Ala by flexizyme. The utilized primer sequence is provided in Table S5. R1, R2, and R3 represent the reactions depicted in Figure S2B. The upper bands represent species b (Figure S2) corresponding to aminoacylated tRNAs after being processed by the $NaIO_4$-RNA extension assay, and the lower bands represent species a (Figure S2) corresponding to free tRNAs after being processed by the $NaIO_4$-RNA extension assay. (B) The correspondence between gel lane numbers in panel A and the respective tRNA species, along with their estimated aminoacylation efficiencies. The sequences of each tRNA are provided in Table S4. Ec = *E. coli*, Ps = *P. syringae*, Tbi = *T. bispora*. The isoforms used were $tRNA^{Ala}$ (UGC), $tRNA^{Cys}$ (GCA) and $tRNA^{Glu}$ (CUC).

A

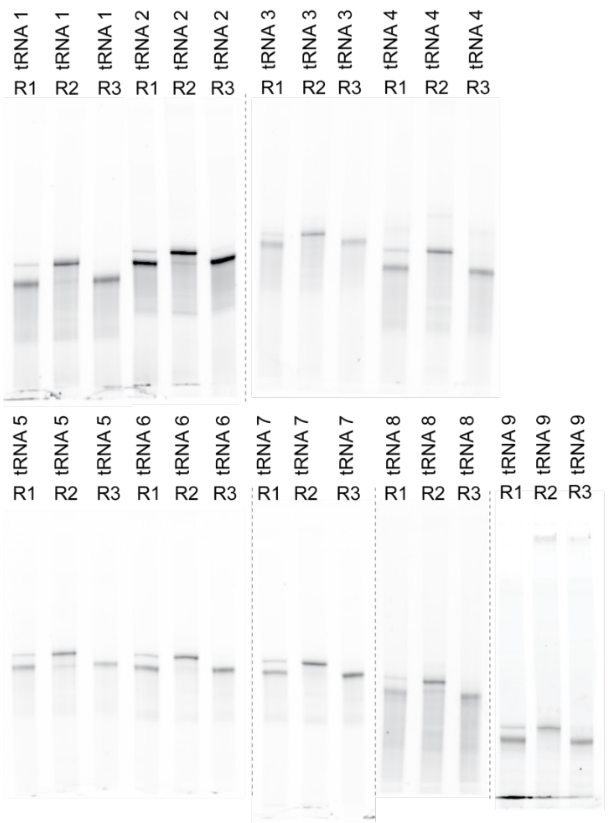

B

RNA	tRNA	Estimated amino-acylation efficiency
tRNA 1	Ec $tRNA^{Trp}$	23%
tRNA 2	Ps $tRNA^{Cys}$	16%
tRNA 3	Ec $tRNA^{Ala}$ grafted with Ps $tRNA^{Cys}$ anticodon arm	26%
tRNA 4	Ec $tRNA^{Ala}$ grafted with Ps $tRNA^{Cys}$ D-Arm	30%
tRNA 5	Tbi $tRNA^{Glu}$	29%
tRNA 6	Ps $tRNA^{Cys}$ (C34G/A35C/U71A)	31%
tRNA 7	Ps $tRNA^{Cys}$ (U71A)	30%
tRNA 8	Ec $tRNA^{Ala}$ grafted with Ec $tRNA^{Trp}$ anticodon arm	29%
tRNA 9	Ec $tRNA^{Ala}$ grafted with Ps tRNA Cys T-Stem	28%

Figure S22. EICs of Δ19BhaA modified by $BhaB_C^{Ala}$ in the presence of *P. syringae* $tRNA^{Cys}$ (GCA) charged with Ala. Each panel represents the $BhaB_C^{Ala}$ reaction when utilizing different amounts of Ala-$tRNA^{Cys}$. The concentration of the substrate peptide (Δ19BhaA) was 1.8 µM. The estimated concentration of Ala-$tRNA^{Cys}$ in each panel was as follows: A) 24 µM, B) 12 µM, C) 5.9 µM, D) 2.9 µM, and E) 1.5 µM. The estimated reaction efficiency is as follows: A) 2.6%, B) 1.8%, C) 1.2%, D) 0.87%, and E) 0.62%. The black trace is the EIC of the substrate Δ19BhaA. The red trace is the EIC of the product Δ19BhaA-Ala. The predicted structure of the examined tRNA is shown in Figure 3 or Figure S19.

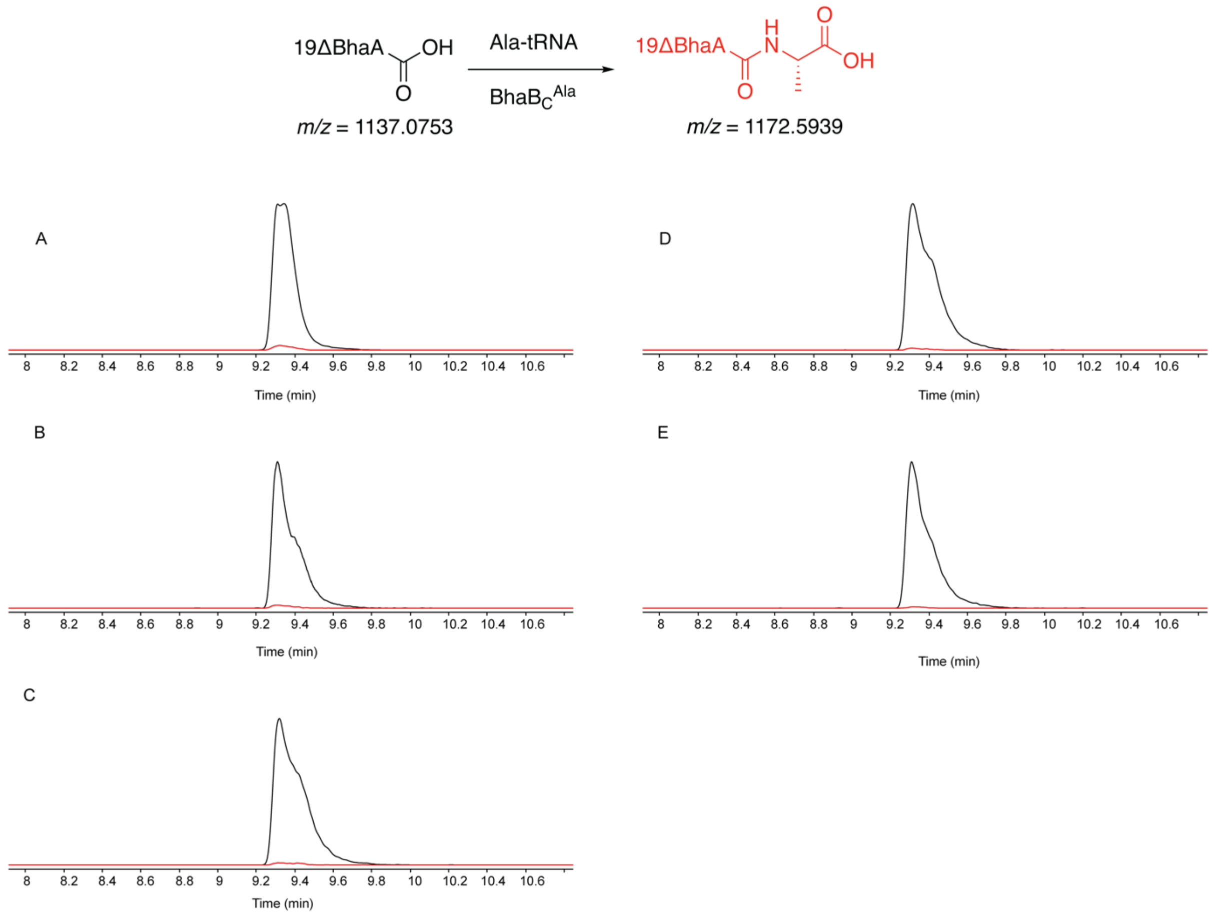

Figure S23. EICs of Δ19BhaA modified by $BhaB_C^{Ala}$ in the presence of *E. coli* $tRNA^{Trp}$ charged with Ala. Each panel represents a $BhaB_C^{Ala}$ reaction when utilizing different amounts of Ala-$tRNA^{Trp}$. The concentration of the substrate peptide (Δ19BhaA) was 1.8 µM. The estimated concentration of Ala-$tRNA^{Trp}$ in each panel was as follows: A) 33 µM, B) 16 µM, C) 8.2 µM, D) 4.1 µM, and E) 2.1 µM. The estimated reaction efficiency is as follows: A) 5.9%, B) 4.0%, C) 2.7%, D) 1.9%, and E) 1.4%. The black trace is the EIC of the substrate Δ19BhaA. The red trace is the EIC of the product Δ19BhaA-Ala. The predicted structure of the examined tRNA can be found in Figure 3 or Figure S19.

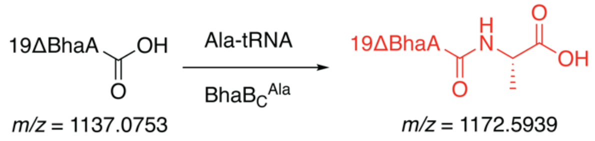

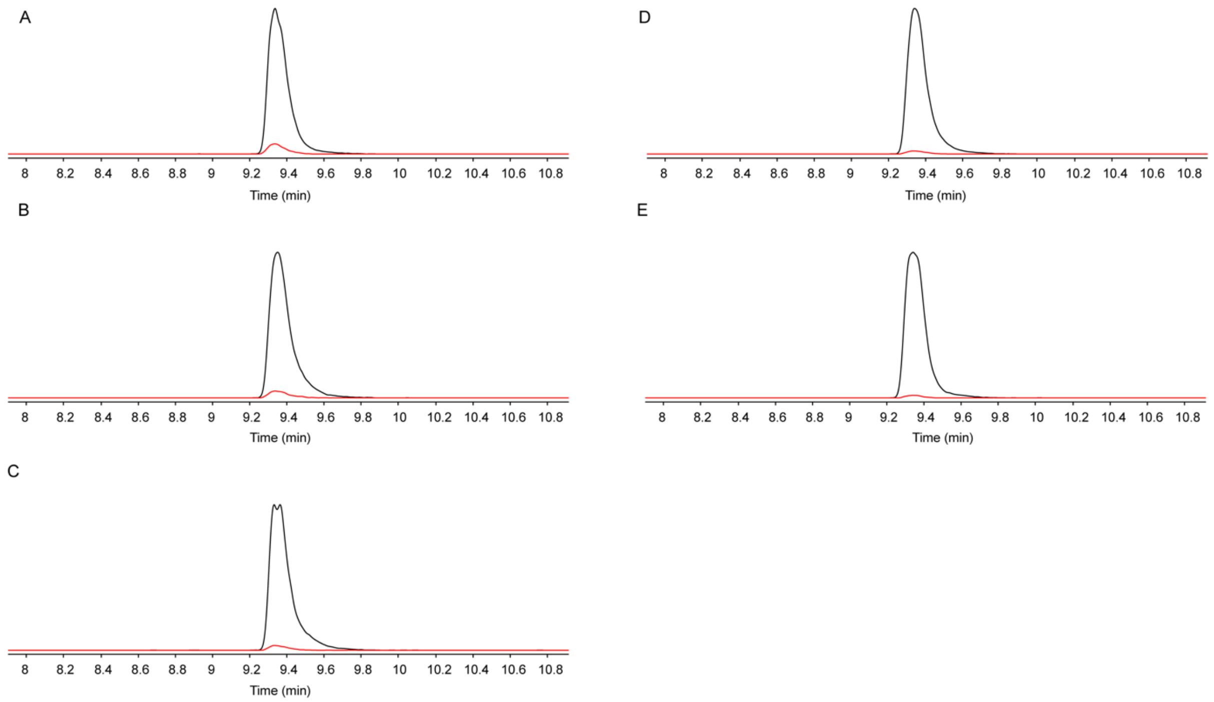

Figure S24. EICs of Δ19BhaA modified by $BhaB_C^{Ala}$ when reacting with Ala charged with *T. bispora* $tRNA^{Glu}$ (CUC). Each panel represents a $BhaB_C^{Ala}$ reaction when utilizing different amounts of Ala-$tRNA^{Glu}$. The concentration of the substrate peptide (Δ19BhaA) was 1.8 µM. The estimated concentration of Ala-$tRNA^{Glu}$ in each panel was as follows: A) 30 µM, B) 15 µM, C) 7.4 µM, D) 3.7 µM, and E) 1.9 µM. The estimated reaction efficiency is as follows: A) 93%, B) 72%, C) 46%, D) 29%, and E) 18%. The black trace is the EIC of the substrate Δ19BhaA. The red trace is the EIC of the product Δ19BhaA-Ala. The predicted structure of the examined tRNA can be found in Figure 3 or Figure S19.

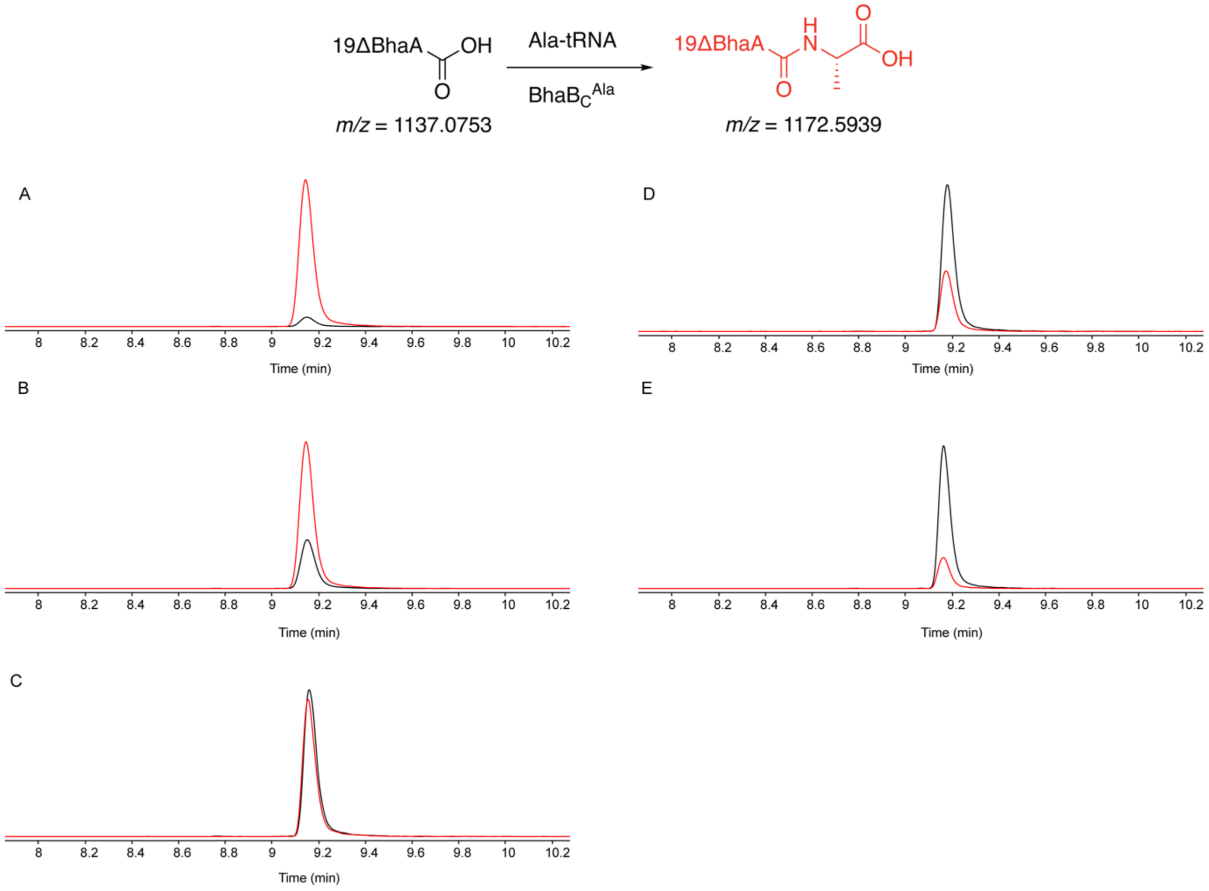

Figure S25. EICs of Δ19BhaA modified by $BhaB_C^{Ala}$ when reacting with Ala charged with *E. coli* $tRNA^{Ala}$ (UGC) grafted with the D-arm of *P. syringae* $tRNA^{Cys}$ ($tRNA^{Ala\text{-}Cys(D)}$). The sequence is provided in Table S4. Each panel represents a $BhaB_C^{Ala}$ reaction when utilizing different amounts of Ala-$tRNA^{Ala\text{-}Cys(D)}$ (**Chimera 1**). The concentration of the substrate peptide **(**Δ19BhaA) was 1.8 µM. The estimated concentration of Ala-$tRNA^{Ala\text{-}Cys(D)}$ in each panel was as follows: A) 30 µM, B) 15 µM, C) 7.6 µM, D) 3.8 µM, and E) 1.9 µM. The estimated reaction efficiency is as follows: A) 99%, B) 98%, C) 73%, D) 35%, and E) 17%. The black trace is the EIC of the substrate Δ19BhaA. The red trace is the EIC of the product Δ19BhaA-Ala. The predicted structure of the examined tRNA can be found in Figure 3.

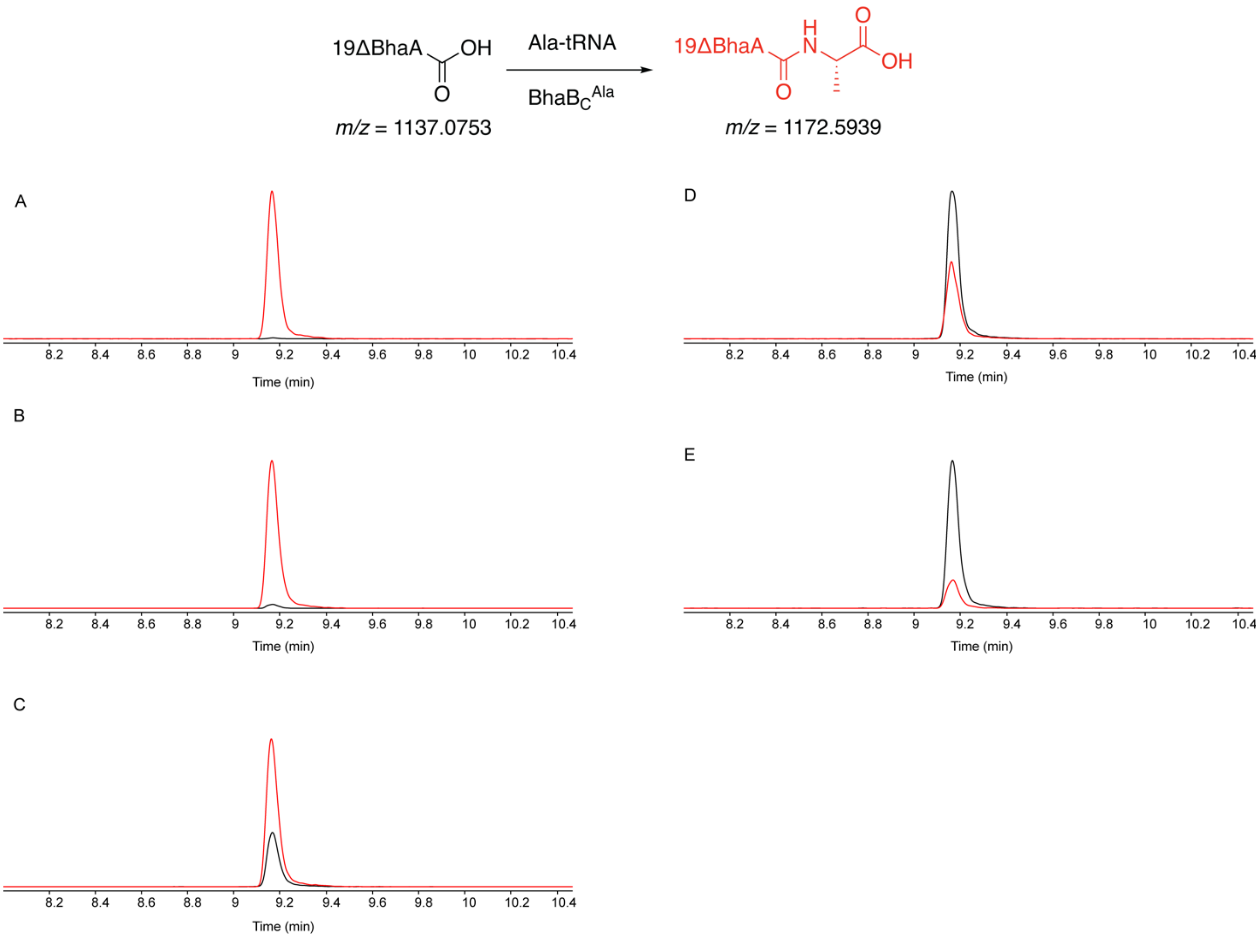

Figure S26. EICs of Δ19BhaA modified by $BhaB_C^{Ala}$ when reacting with Ala charged with *E. coli* $tRNA^{Ala}$ (UGC) grafted with the T-stem of *P. syringae* $tRNA^{Cys}$ ($tRNA^{Ala\text{-}Cys(T)}$). The sequence can is provided in Table S4. Each panel represents a $BhaB_C^{Ala}$ reaction when utilizing different amounts of Ala-$tRNA^{Ala\text{-}Cys(T)}$ (**Chimera 2**). The concentration of the substrate peptide (Δ19BhaA) was 1.8 µM. The estimated concentration of Ala-$tRNA^{Ala\text{-}Cys(T)}$ in each panel was as follows: A) 34 µM, B) 17 µM, C) 8.5 µM, D) 4.3 µM, and E) 2.1 µM. The estimated reaction efficiency is as follows: A) 98%, B) 99%, C) 99%, D) 99%, and E) 79%. The black trace is the EIC of the substrate Δ19BhaA. The red trace is the EIC of the product Δ19BhaA-Ala. The predicted structure of the examined tRNA can be found in Figure 3.

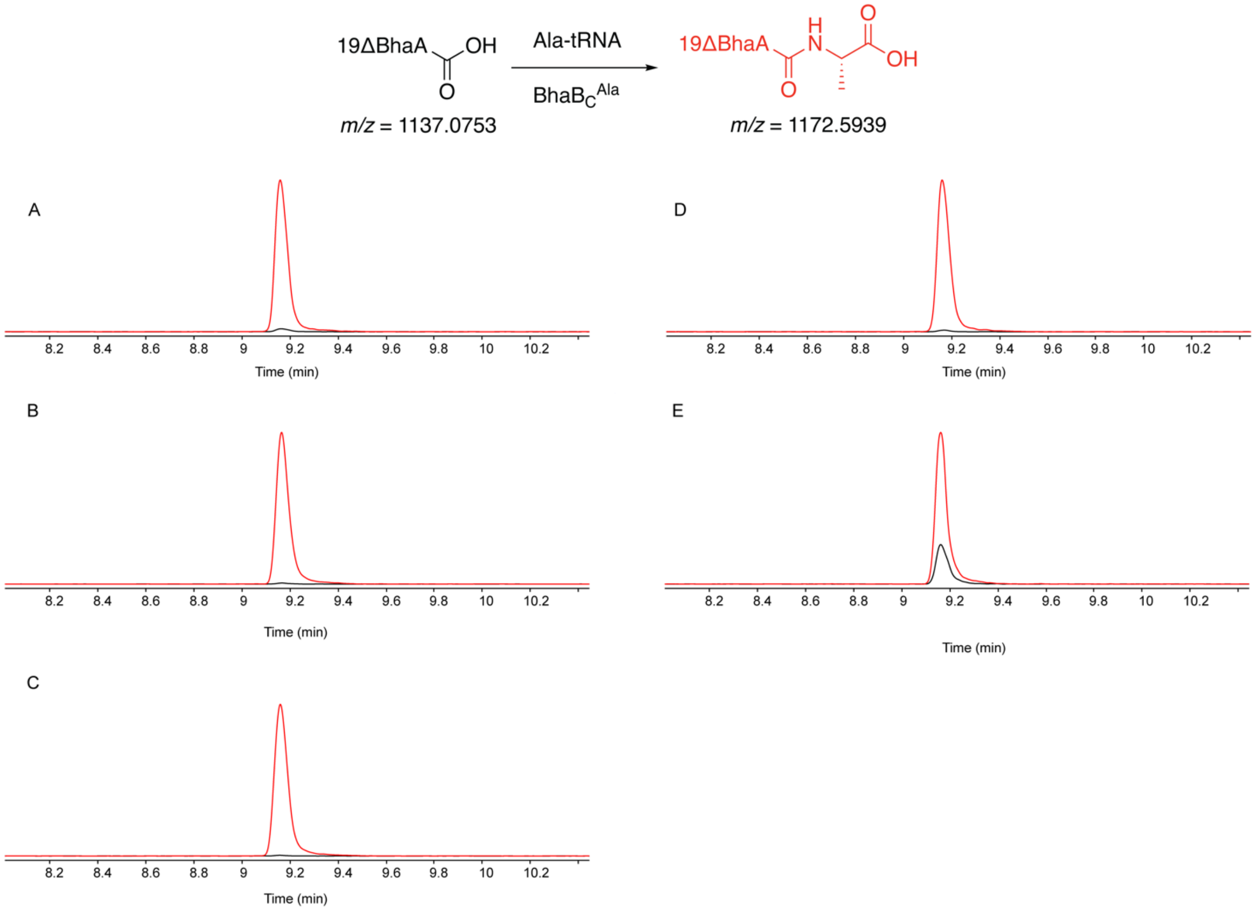

Figure S27. EICs of Δ19BhaA modified by $BhaB_C^{Ala}$ when reacting with Ala charged to *E. coli* $tRNA^{Ala}$ (UGC) grafted with the anticodon Arm of *P. syringae* $tRNA^{Cys}$ ($tRNA^{Ala\text{-}Cys(Anti)}$). The sequence is provided in Table S4. Each panel represents a $BhaB_C^{Ala}$ reaction when utilizing different amounts of Ala-$tRNA^{Ala\text{-}Cys(Anti)}$ (**Chimera 3**). The concentration of the substrate peptide (Δ19BhaA) was 1.8 μM. The estimated concentration of Ala-$tRNA^{Ala\text{-}Cys(Anti)}$ in each panel was as follows: A) 30 μM, B) 15 μM, C) 7.6 μM, D) 3.8 μM, and E) 1.9 μM. The estimated reaction efficiency is as follows: A)33%, B) 13%, C) 4.5%, D) 2.3%, and E) 1.8%. The black trace is the EIC of the substrate Δ19BhaA. The red trace is the EIC of the product Δ19BhaA-Ala. The predicted structure of the examined tRNA can be found in Figure 3.

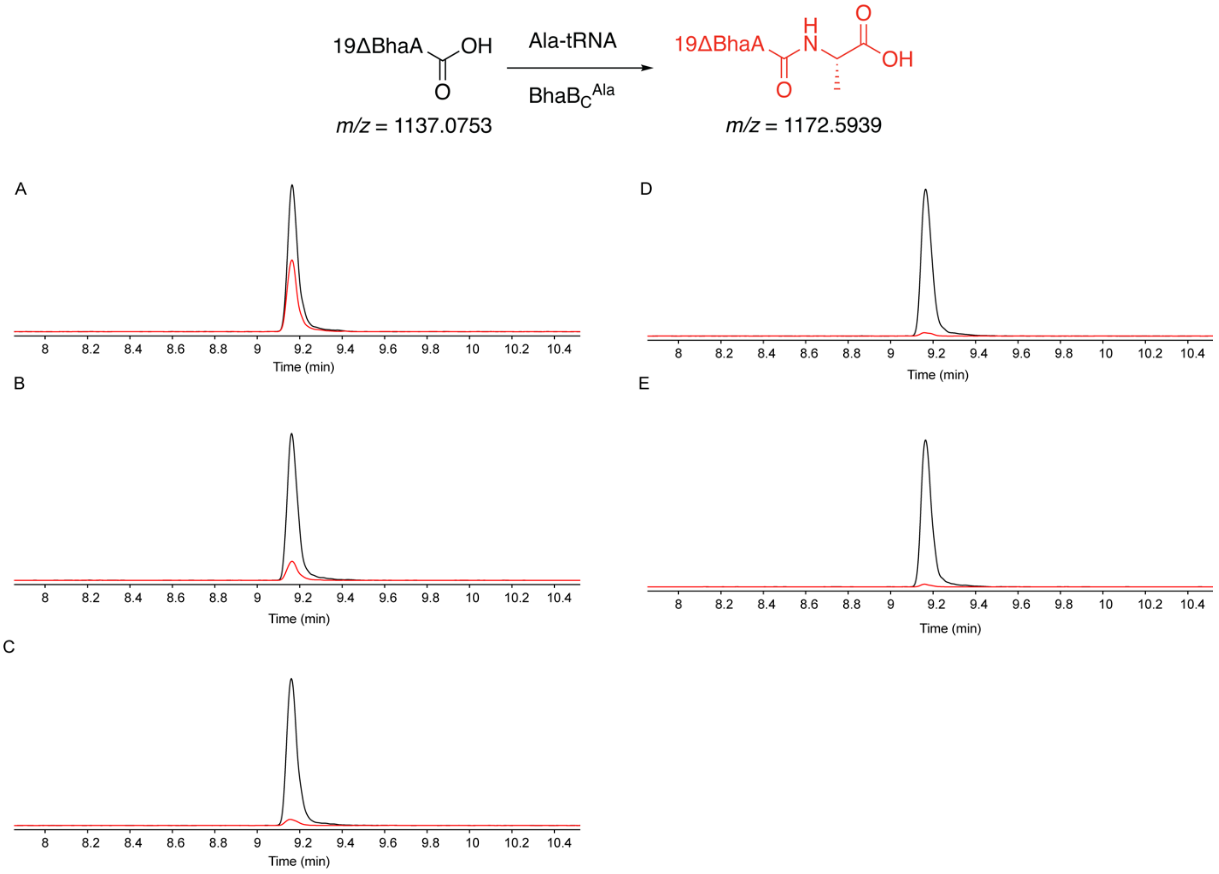

Figure S28. EICs of Δ19BhaA modified by $BhaB_C^{Ala}$ when reacting with Ala charged to *E. coli* $tRNA^{Ala}$ (UGC) grafted with the anticodon arm of *E. coli* $tRNA^{Trp}$ ($tRNA^{Ala\text{-}Trp(Anti)}$). The sequence is provided in Table S4. Each panel represents a $BhaB_C^{Ala}$ reaction when utilizing different amounts of Ala-$tRNA^{Ala\text{-}Trp(Anti)}$ (**Chimera 4**). The concentration of the substrate peptide (Δ19BhaA) was 1.8 µM. The estimated concentration of Ala-$tRNA^{Ala\text{-}Trp(Anti)}$ in each panel was as follows: A) 31 µM, B) 15 µM, C) 7.7 µM, D) 3.9 µM, and E) 1.9 µM. The estimated reaction efficiency is as follows: A) 21%, B) 6.2%, C) 2.1%, D) 1.2%, and E) 0.78%. The black trace is the EIC of the substrate Δ19BhaA. The red trace is the EIC of the product Δ19BhaA-Ala. F) The predicted structure of the examined tRNA.

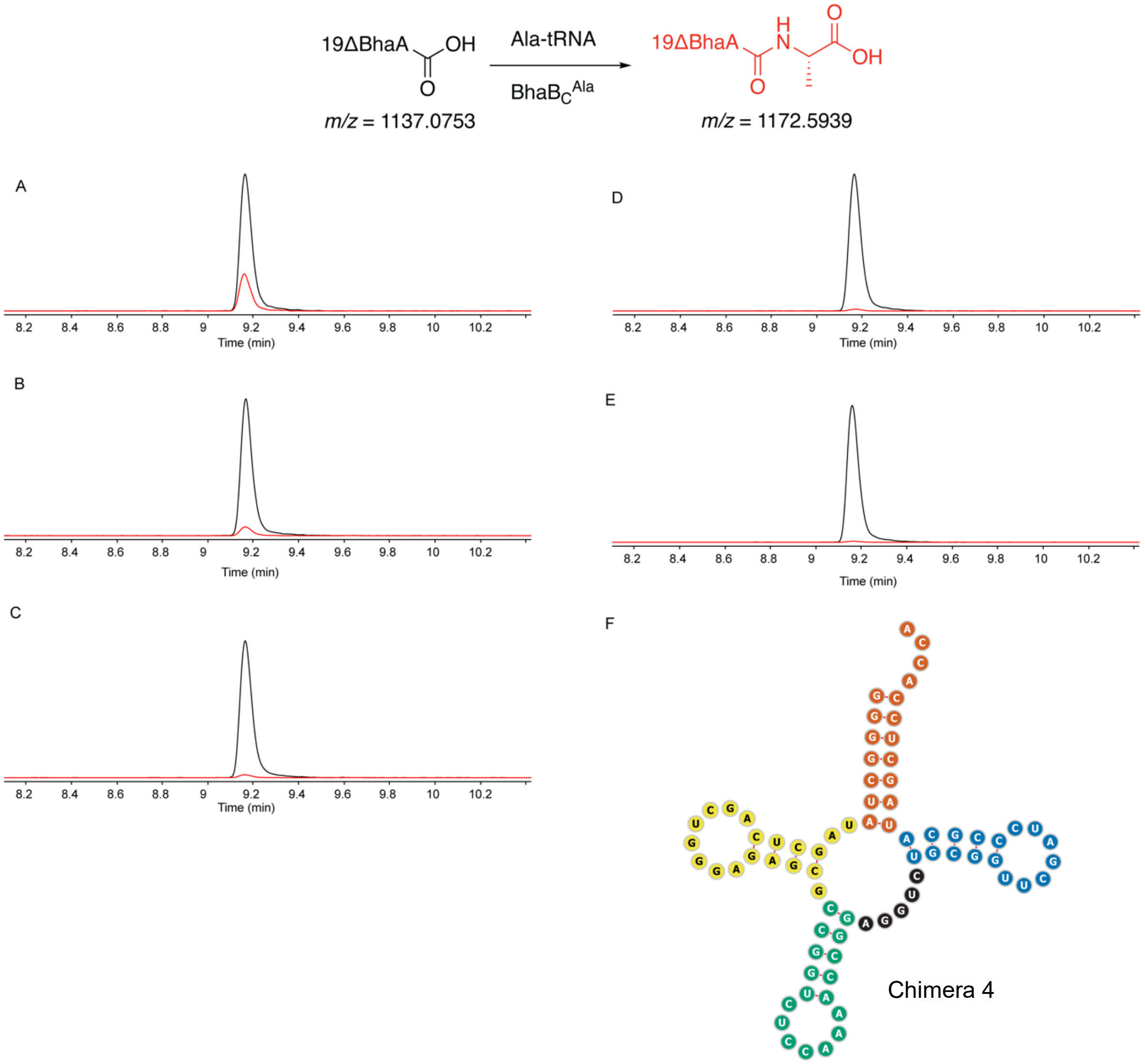

Figure S29. EICs of Δ19BhaA modified by $BhaB_C^{Ala}$ when reacting with Ala charged onto *P. syringae* $tRNA^{Ala}$ (GCA) (U71A). Each panel represents a $BhaB_C^{Ala}$ reaction when utilizing different amounts of Ala-tRNA. The concentration of the substrate peptide (Δ19BhaA) was 1.8 µM. The estimated concentration of Ala-tRNA in each panel was as follows: A) 30 µM, B) 15 µM, C) 7.6 µM, D) 3.8 µM, and E) 1.9 µM. The estimated reaction efficiency is as follows: A) 47%, B) 27%, C) 14%, D) 9.4%, and E) 6.4%. The black trace is the EIC of the substrate Δ19BhaA. The red trace is the EIC of the product Δ19BhaA-Ala. The predicted structure of the examined tRNA can be found in Figure 3.

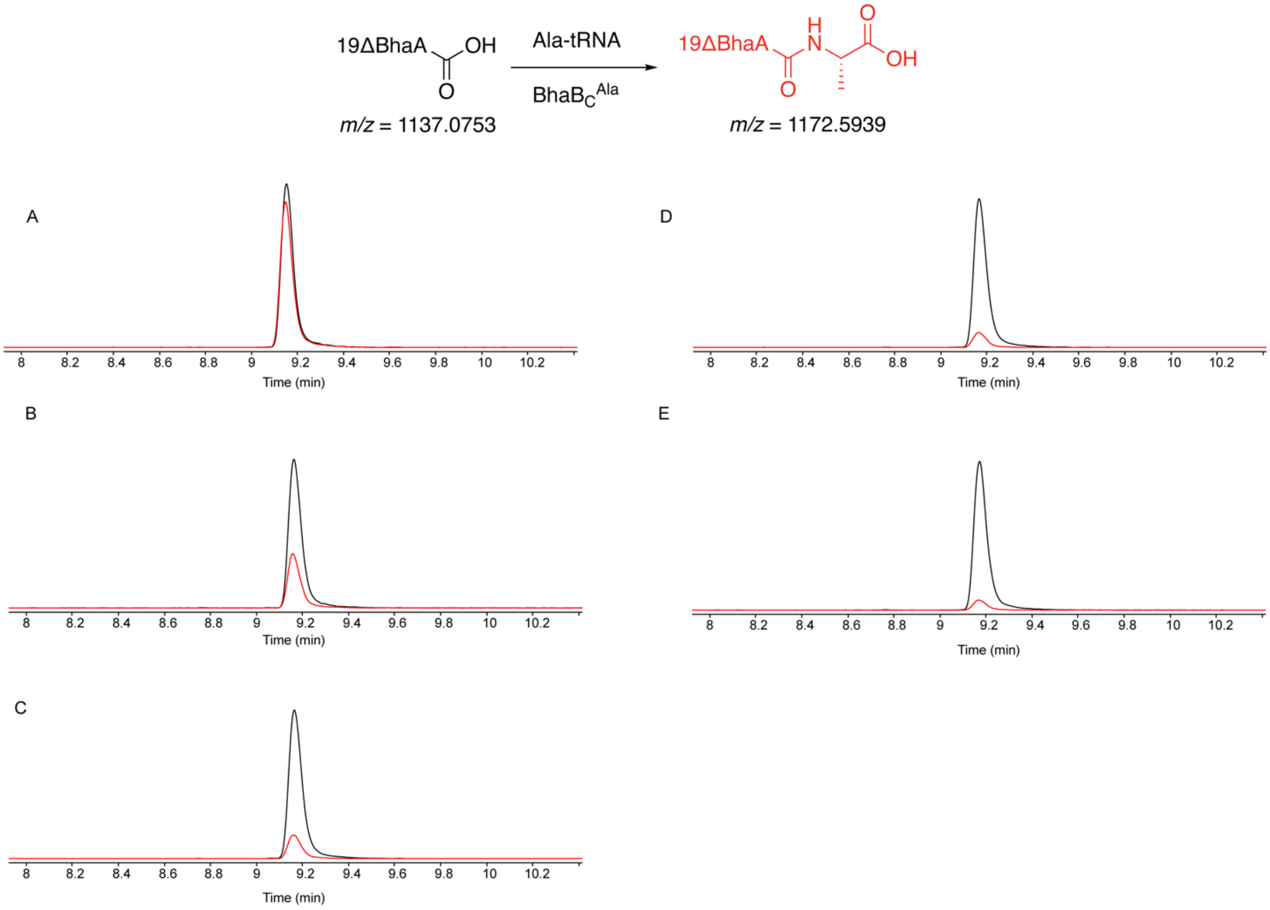

Figure S30. EICs of Δ19BhaA modified by $BhaB_C^{Ala}$ when reacting with Ala charged with *P. syringae* $tRNA^{Ala}$ (GCA) (C34G/A35C/U71A). The discriminator base and the anticodon of this non-cognate tRNA match those of *E. coli* $tRNA^{Ala}$ (GGC). Each panel represents a $BhaB_C^{Ala}$ reaction when utilizing different amounts of Ala-tRNA. The concentration of the substrate peptide (Δ19BhaA) was 1.8 μM. The estimated concentration of Ala-tRNA in each panel was as follows: A) 30 μM, B) 15 μM, C) 7.5 μM, D) 3.8 μM, and E) 1.9 μM. The estimated reaction efficiency is as follows: A) 85%, B) 57%, C) 34%, D) 20%, and E) 13%. The black trace is the EIC of the substrate Δ19BhaA. The red trace is the EIC of the product Δ19BhaA-Ala. The predicted structure of the examined tRNA can be found in Figure 3.

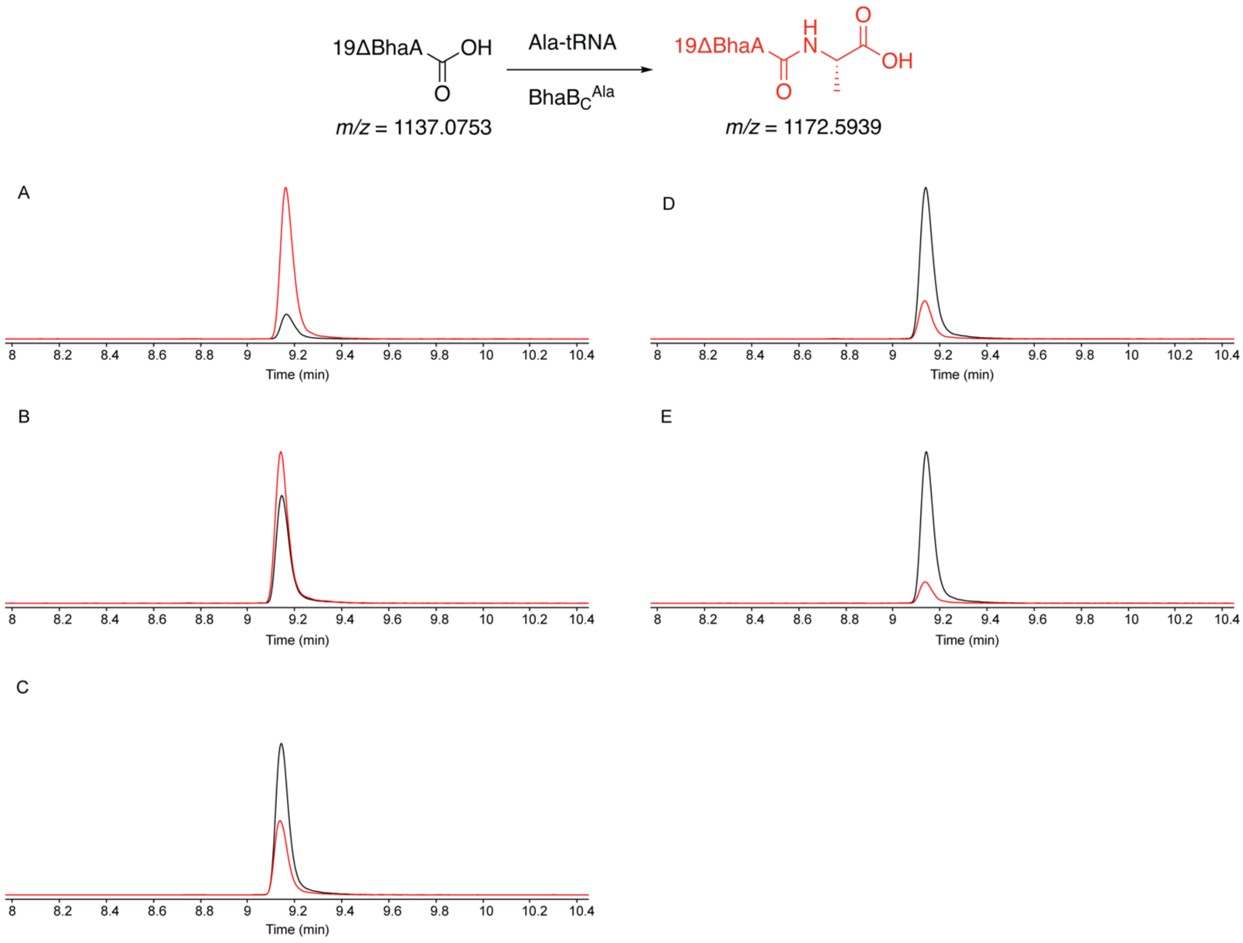

Figure S31. Alphafold3 model of the BhaB$_C^{Ala}$-BhaA-tRNA complex. A) The structure includes BhaB$_C^{Ala}$, BhaA, *E. coli* tRNA^{Ala} (UGC), Mg^{2+}, and ATP. The tRNA is colored by structural region: orange (acceptor stem), yellow (D-arm), green (anticodon arm), black (variable arm), and blue (T-arm). The figure was made using Chimera.[26] The tRNA cloverleaf structure was generated using tRNAScanSE[21] and Forna.[22] B) Alphafold3 model colored by predicted local distance difference test (pLDDT) scores (x); C) Predicted aligned error (pAE) plot for the model. The model file can be found in Supplementary Data 1.

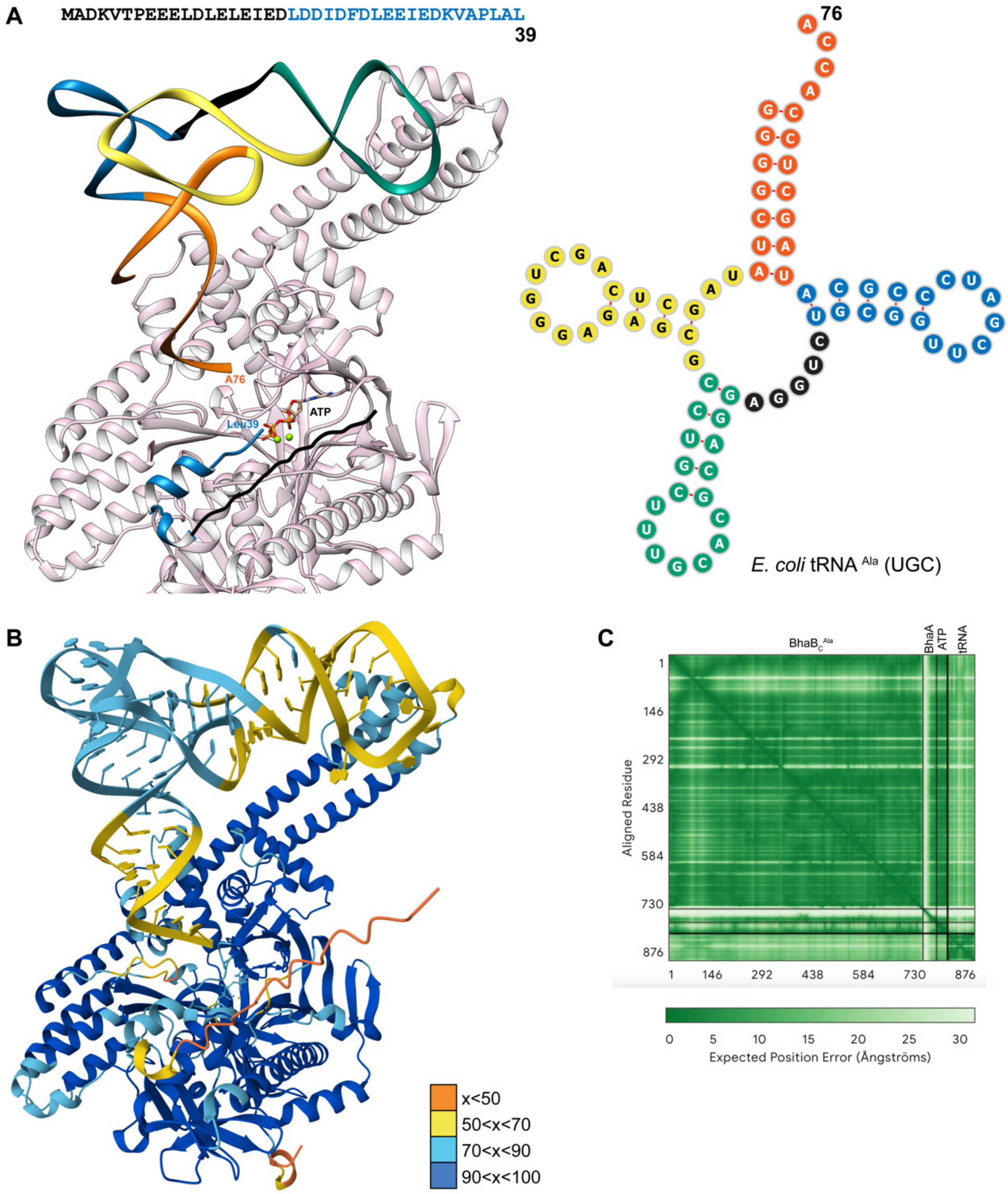

Figure S32. An active site view of the Alphafold3 model of BhaA, $BhaB_C^{Ala}$, ATP, Mg^{2+}, and *E. coli* $tRNA^{Ala}$(UGC) depicting that the first 19 residues of BhaA are not predicted to interact with $BhaB_C^{Ala}$. The peptide sequence is displayed above the model, with colors corresponding to those used in the AlphaFold3 model. The depicted Leu39 is the C-terminal residue of BhaA. The figure was made using Chimera.[26]

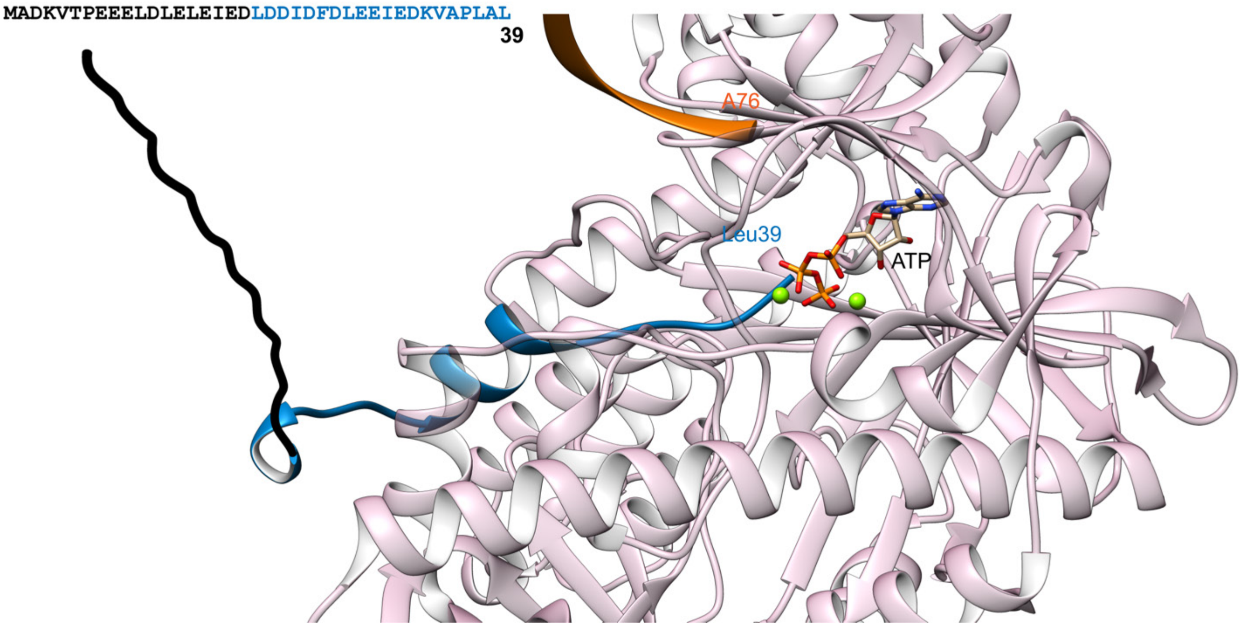

Figure S33. (A) PAGE analysis of RNAs used in this study. The ladder is low range ssRNA ladder (NEB). (B) The correspondence between indicated gel lane numbers and the respective tRNA species. The sequences of each tRNA are provided in Table S4. Ec = *E. coli*, Ps = *P. syringae*, Tbi = *T. bispora*. $tRNA^{Ala}$ here is $tRNA^{Ala}$ (UGC), tRNACys here is $tRNA^{Cys}$ (GCA), and $tRNA^{Glu}$ here is $tRNA^{Glu}$ (CUC).

A

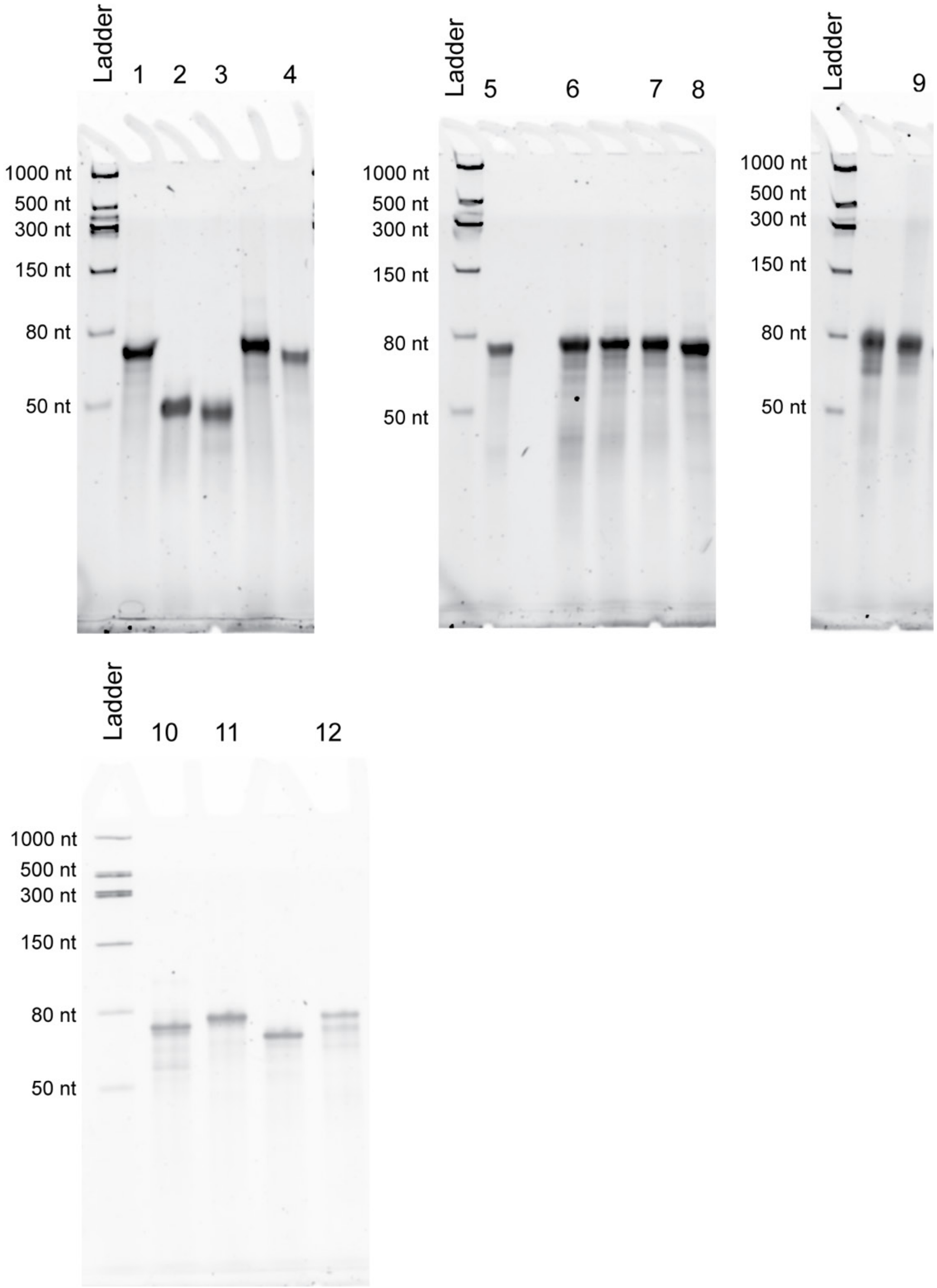

B

Number	RNA
1	Ec $tRNA^{Ala}$
2	dFx
3	eFx
4	Ec $tRNA^{Ala}$ grafted with Ec $tRNA^{Trp}$ Anticodon Arm
5	Ps $tRNA^{Cys}$
6	Ps $tRNA^{Cys}$ (U71A)
7	Ps $tRNA^{Cys}$ (C34G/A35C/U71A)
8	Tbi $tRNA^{Glu}$
9	Ec $tRNA^{Trp}$
10	Ec $tRNA^{Ala}$ grafted with Ps $tRNA^{Cys}$ D-Arm
11	Ec $tRNA^{Ala}$ grafted with Ps $tRNA^{Cys}$ T-Stem
12	Ec $tRNA^{Ala}$ grafted with Ps $tRNA^{Cys}$ Anticodon Arm

Table S1. Activated amino acids used in this study as substrates for flexizymes along with summary of tested flexizyme conditions. CBT = 4-chlorobenzyl thioester, DBE = 3,5-dinitrobenzyl ester, CME = cyanomethyl ester.

**Amino Acid, Concentration**	**Flexizyme**	**pH**	**[Mg^{2+}]**	**Reaction time**	**% DMSO (*v/v*)**
Ala-DBE, 5 mM	dFx	7.5	100 mM	6 h	20
Glu-DBE, 5 mM	dFx	7.5	100 mM	6 h	20
Gln-DBE, 5 mM	dFx	7.5	100 mM	6 h	20
(N-Me-Ala)-DBE, 5 mM	dFx	7.5	100 mM	6 h	20
(D-Ala)-DBE, 5 mM	dFx	7.5	100 mM	6 h	20
Aib-DBE, 5 mM	dFx	7.5	600 mM	2.5 h	20
ThioGly-DBE, 5 mM	dFx	9.0	600 mM	6 h	40
Phe-CME, 5 mM	eFx	7.5	600 mM	2.5 h	20
Trp-CME, 5 mM	eFx	7.5	600 mM	2.5 h	20
Ala-CBT, 5 mM	eFx	7.5	600 mM	2.5 h	20
Gly-CBT, 5 mM	eFx	7.5	600 mM	2.5 h	20
(β-Ala)-CBT, 5 mM	eFx	7.5	600 mM	2.5 h	20
Lac-CBT, 5 mM	eFx	7.5	482 mM	2.5 h	32

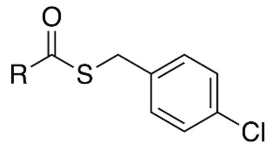

Acid charged with CBT

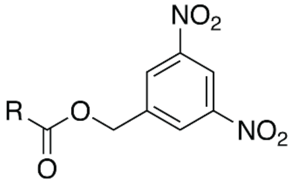

Acid charged with DBE

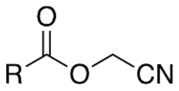

Acid charged with CME

Table S2. Summary of $BhaB_C^{Ala}$ activity using non-cognate tRNAs charged with L-Ala. The EICs are shown in Figures S22-S30. Ec = *E. coli*, Ps = *P. syringae*, Tbi = *T. bispora*. $tRNA^{Ala}$ here is $tRNA^{Ala}$ (UGC), $tRNA^{Cys}$ is $tRNA^{Cys}$ (GCA), and $tRNA^{Glu}$ is $tRNA^{Glu}$ (CUC).

**Ala-tRNA examined**	**Estimated [Ala-tRNA], reaction efficiency**				
Ala-Ps $tRNA^{Cys}$	24 µM, 2.6%	12 µM, 1.8%	5.9 µM, 1.2%	2.9 µM, 0.87%	1.5 µM, 0.62%
Ala-Ps $tRNA^{Cys}$ (U71A)	30 µM, 47%	15 µM, 27%	7.6 µM, 14%	3.8 µM, 9.4%	1.9 µM, 6.4%
Ala-Ps $tRNA^{Cys}$ (C34G/A35C/U71A)	30 µM, 85%	15 µM, 57%	7.5 µM, 34%	3.8 µM, 20%	1.9 µM, 13%
Ala-Ec $tRNA^{Ala}$ grafted with Ps $tRNA^{Cys}$ D-Arm	30 µM, 99%	15 µM, 98%	7.6 µM, 73%	3.8 µM, 35%	1.9 µM, 17%
Ala-Ec $tRNA^{Ala}$ grafted with Ps $tRNA^{Cys}$ T-Stem	34 µM, 98%	17 µM, 99%	8.5 µM, 99%	4.3 µM, 99%	2.1 µM, 79%
Ala-Ec $tRNA^{Ala}$ grafted with Ps $tRNA^{Cys}$ Anticodon Arm	30 µM, 33%	15 µM, 13%	7.6 µM, 4.5%	3.8 µM, 2.3%	1.9 µM, 1.8%
Ala-Ec $tRNA^{Trp}$	33 µM, 5.9%	16 µM, 4.0%	8.2 µM, 2.7%	4.1 µM, 1.9%	2.1 µM, 1.4%
Ala-Ec $tRNA^{Ala}$ grafted with Ec $tRNA^{Trp}$ Anticodon Arm	31 µM, 21%	15 µM, 6.2%	7.7 µM, 2.1%	3.9 µM, 1.2%	1.9 µM, 0.78%
Ala-Tbi $tRNA^{Glu}$	30 µM, 93%	15 µM, 72%	7.4 µM, 46%	3.7 µM, 29%	1.9 µM, 18%

Table S3. Sequence of the plasmid used to express $BhaB_C^{Ala}$ (NCBI accession: BAB05753.1). *E. coli* codon-optimized DNA sequence encoding $BhaB_C^{Ala}$ is highlighted in red.

taatacgactcactataggggaattgtgagcggataacaattcccctctagaaataattttgtttaactttaag
aaggagatataccatgggcagcagccatcatcatcatcatcacagcagcggcctggtgccgcgcggcagccata
tgtggttcaatacttccacatggcgttggttaaagccatttgtgctccgcagtacctctatccccatcgagatg
gttttgaacttaaggttaaaagaatcaataaagcaactaaatgaatggagcaaaagccaacaatcatttgatca
atggtgcaaccattggattaaagaaattaggtctgtaccagtagagcgaataagtcttccgcaccaacgaaaag
tcatacgaagaataaaaaaaagaaacccactaaccgaacaagataaaatcattgttaccaagtggggaatggaa
aagttgattgatgagtactgctcacagcagcaatcattaataagcttaaagaaaaaggcgcaacaatcttttca
tgaagagtttgaacgactacaacaacagttaattgagaggtttcaatctagccaatatgaattagctctgtata
cattgaaccacaagctttggcatttctggattaaggagaatcgtcttgaaaatctaactgatgcacaaaaaaaa
caaacatgtagaacccttttcgcctatcttcaacgggtctcaactaaaaatgacactatcggtgaatatggccc
tatcagttatggtgcattcaatcaaggcaagctgattcaaactaggcaaattacgaaaagaaaagcctatttttg
cctatcaagggctacaaaaattgctaagtgttataaaaaaggatttaatgccgcaggctcatgcctggaatctc
ccatcgtccacggtagatgggtggaagcttgttagagaacgggttgaagattgtaatgatcctcgttcagaaat
atggatacgaatcctagatcaacttgaggagactagacatttgtttgagacgtccactcaaccaaacgaaaaag
aacaagccttagctcaagctgagaaactatttacaaacataacaggggaggcttttcaaggagagaatcagcaa
tacttcgcagataaaacattgctgtatgaagaatgccatgatcagcaatatttacccgtcaccctatctgtcga
tttatggacagagttagatcatgctttgttgattcatgccaaactacgcctagacatttggagatcttggcaaa
atattgcggtccagaaattcgagcaattggcgaatggaaatgtttcgattccgttaatgaaatgggtttcctat
tggttgcgttatcctccaagccttgacaaccaatcagcaaccgatctgctttcaccttggctagttgatcgtaa
tggctacctttcaattaaactcccggaccgagttatccgaagctggcggaaggagattaactcattcagatggt
tactctcacctgatttaatggtaaaaggaaaatttgagactaatcgtttcaatgtagcatccatcgtgataggg
gaattacatcacggttttaccgccgatggttggatgtttgaatttcatcctgacaaaaaagaaatcaatcgaat
agtccggcaagctatgccagaaaagaatccaagctttacttgggctaattggatctttcaaaggaaaatgaaaa
gtactccgcaagagtacccaggtgtatcagtaaagcttacaggtcattctgaatacccagatgagaagtcattc
tctctatatgacttggaggttagaaggttaggcgataaagtggctgtttttttacaaggaactgataatgctct
aatgttttatcccccctgcctatggatttaacgaaacaagcttttttccccttttgcacttttctgttcacctatga
tggaaccttcatccaattactcaaaagggagacacaaagcaataaagttaggaaatgtcacattaattagggaa
cattgggtttcgagccgacagattggggagaagtccatgcatcacttgatggatttcaacaaatggcgtgttt
acaagagattaaggttaaatatcaattaccgcaagtaggatacttaagattttctcaagagctaaaacctgttt
ggtttgactttaataatccatttgtgtcgatttgtttttcaatatggctaaaaaagcagaatggtttacgttt
tccagaatggacccaggggtggaggatttatttctgaaggatgatcgaggccattattgttgtgaattgcgaac
ctttgcttatcggcaagagataacttccaaagatatgctcatgggttaactcgagcaccaccaccaccaccact
gagatccggctgctaacaaagcccgaaaggaagctgagttggctgctgccaccgctgagcaataactagcataa
ccccttggggcctctaaacgggtcttgaggggttttttgctgaaaggaggaactatatccggattggcgaatgg
gacgcgccctgtagcggcgcattaagcgcggcgggtgtggtggttacgcgcagcgtgaccgctacacttgccag
cgccctagcgcccgctcctttcgctttcttcccttcctttctcgccacgttcgccggctttccccgtcaagctc
taaatcgggggctccctttagggttccgatttagtgctttacggcacctcgaccccaaaaaacttgattagggt
gatggttcacgtagtgggccatcgccctgatagacggtttttcgccctttgacgttggagtccacgttctttaa
tagtggactcttgttccaaactggaacaacactcaaccctatctcggtctattcttttgatttataagggattt
tgccgatttcggcctattggttaaaaaatgagctgatttaacaaaaatttaacgcgaattttaacaaaatatta
acgcttacaatttaggtggcacttttcggggaaatgtgcgcggaacccctatttgtttatttttctaaatacat
tcaaatatgtatccgctcatgaattaattcttagaaaaactcatcgagcatcaaatgaaactgcaatttattca
tatcaggattatcaataccatatttttgaaaaagccgtttctgtaatgaaggagaaaactcaccgaggcagttc
cataggatggcaagatcctggtatcggtctgcgattccgactcgtccaacatcaatacaacctattaatttccc
ctcgtcaaaaataaggttatcaagtgagaaatcaccatgagtgacgactgaatccggtgagaatggcaaaagtt
tatgcatttctttccagacttgttcaacaggccagccattacgctcgtcatcaaaatcactcgcatcaaccaaa
ccgttattcattcgtgattgcgcctgagcgagacgaaatacgcgatcgctgttaaaaggacaattacaaacagg
aatcgaatgcaaccggcgcaggaacactgccagcgcatcaacaatattttcacctgaatcaggatattcttcta
atacctggaatgctgttttcccggggatcgcagtggtgagtaaccatgcatcatcaggagtacggataaaatgc
ttgatggtcggaagaggcataaattccgtcagccagtttagtctgaccatctcatctgtaacatcattggcaac
gctacctttgccatgtttcagaaacaactctggcgcatcgggcttcccatacaatcgatagattgtcgcacctg

attgcccgacattatcgcgagcccatttatacccatataaatcagcatccatgttggaatttaatcgcggccta
gagcaagacgtttcccgttgaatatggctcataacaccccttgtattactgtttatgtaagcagacagttttat
tgttcatgaccaaaatcccttaacgtgagttttcgttccactgagcgtcagaccccgtagaaaagatcaaagga
tcttcttgagatcctttttttctgcgcgtaatctgctgcttgcaaacaaaaaaaccaccgctaccagcggtggt
ttgtttgccggatcaagagctaccaactctttttccgaaggtaactggcttcagcagagcgcagataccaaata
ctgtccttctagtgtagccgtagttaggccaccacttcaagaactctgtagcaccgcctacatacctcgctctg
ctaatcctgttaccagtggctgctgccagtggcgataagtcgtgtcttaccgggttggactcaagacgatagtt
accggataaggcgcagcggtcgggctgaacggggggttcgtgcacacagcccagcttggagcgaacgacctaca
ccgaactgagatacctacagcgtgagctatgagaaagcgccacgcttcccgaagggagaaaggcggacaggtat
ccggtaagcggcagggtcggaacaggagagcgcacgagggagcttccagggggaaacgcctggtatctttatag
tcctgtcgggtttcgccacctctgacttgagcgtcgatttttgtgatgctcgtcaggggggcggagcctatgga
aaaacgccagcaacgcggcctttttacggttcctggccttttgctggccttttgctcacatgttctttcctgcg
ttatcccctgattctgtggataaccgtattaccgcctttgagtgagctgataccgctcgccgcagccgaacgac
cgagcgcagcgagtcagtgagcgaggaagcggaagagcgcctgatgcggtattttctccttacgcatctgtgcg
gtatttcacaccgcaatggtgcactctcagtacaatctgctctgatgccgcatagttaagccagtatacactcc
gctatcgctacgtgactgggtcatggctgcgccccgacacccgccaacacccgctgacgcgccctgacgggctt
gtctgctcccggcatccgcttacagacaagctgtgaccgtctccgggagctgcatgtgtcagaggttttcaccg
tcatcaccgaaacgcgcgaggcagctgcggtaaagctcatcagcgtggtcgtgaagcgattcacagatgtctgc
ctgttcatccgcgtccagctcgttgagtttctccagaagcgttaatgtctggcttctgataaagcgggccatgt
taagggcggtttttttcctgtttggtcactgatgcctccgtgtaagggggatttctgttcatgggggtaatgata
ccgatgaaacgagagaggatgctcacgatacgggttactgatgatgaacatgcccggttactggaacgttgtga
gggtaaacaactggcggtatggatgcggcgggaccagagaaaaatcactcagggtcaatgccagcgcttcgtta
atacagatgtaggtgttccacagggtagccagcagcatcctgcgatgcagatccggaacataatggtgcagggc
gctgacttccgcgtttccagactttacgaaacacggaaaccgaagaccattcatgttgttgctcaggtcgcaga
cgttttgcagcagcagtcgcttcacgttcgctcgcgtatcggtgattcattctgctaaccagtaaggcaacccc
gccagcctagccgggtcctcaacgacaggagcacgatcatgcgcacccgtggggccgccatgccggcgataatg
gcctgcttctcgccgaaacgtttggtggcgggaccagtgacgaaggcttgagcgagggcgtgcaagattccgaa
taccgcaagcgacaggccgatcatcgtcgcgctccagcgaaagcggtcctcgccgaaaatgacccagagcgctg
ccggcacctgtcctacgagttgcatgataaagaagacagtcataagtgcggcgacgatagtcatgccccgcgcc
caccggaaggagctgactgggttgaaggctctcaagggcatcggtcgagatcccggtgcctaatgagtgagcta
acttacattaattgcgttgcgctcactgcccgctttccagtcgggaaacctgtcgtgccagctgcattaatgaa
tcggccaacgcgcggggagaggcggtttgcgtattgggcgccagggtggtttttcttttcaccagtgagacggg
caacagctgattgcccttcaccgcctggccctgagagagttgcagcaagcggtccacgctggtttgccccagca
ggcgaaaatcctgtttgatggtggttaacggcgggatataacatgagctgtcttcggtatcgtcgtatcccact
accgagatatccgcaccaacgcgcagcccggactcggtaatggcgcgcattgcgcccagcgccatctgatcgtt
ggcaaccagcatcgcagtgggaacgatgccctcattcagcatttgcatggtttgttgaaaaccggacatggcac
tccagtcgccttcccgttccgctatcggctgaatttgattgcgagtgagatatttatgccagccagccagacgc
agacgcgccgagacagaacttaatgggcccgctaacagcgcgatttgctggtgacccaatgcgaccagatgctc
cacgcccagtcgcgtaccgtcttcatgggagaaaataatactgttgatgggtgtctggtcagagacatcaagaa
ataacgccggaacattagtgcaggcagcttccacagcaatggcatcctggtcatccagcggatagttaatgatc
agcccactgacgcgttgcgcgagaagattgtgcaccgccgctttacaggcttcgacgccgcttcgttctaccat
cgacaccaccacgctggcacccagttgatcggcgcgagatttaatcgccgcgacaatttgcgacggcgcgtgca
gggccagactggaggtggcaacgccaatcagcaacgactgtttgcccgccagttgttgtgccacgcggttggga
atgtaattcagctccgccatcgccgcttccactttttcccgcgttttcgcagaaacgtggctggcctggttcac
cacgcgggaaacggtctgataagagacaccggcatactctgcgacatcgtataacgttactggtttcacattca
ccaccctgaattgactctcttccgggcgctatcatgccataccgcgaaaggttttgcgccattcgatggtgtcc
gggatctcgacgctctcccttatgcgactcctgcattaggaagcagcccagtagtaggttgaggccgttgagca
ccgccgccgcaaggaatggtgcatgcaaggagatggcgcccaacagtcccccggccacggggcctgccaccata
cccacgccgaaacaagcgctcatgagcccgaagtggcgagcccgatcttccccatcggtgatgtcggcgatata
ggcgccagcaaccgcacctgtggcgccggtgatgccggccacgatgcgtccggcgtagaggatcgagatctcga
tcccgcgaaat

Table S4. Sequences of tRNA molecules and the corresponding primers used for assembling templates for *in vitro* transcription. All sequences are provided 5' to 3' (left to right). Lowercase m indicates 2' *O*-methylation of the following residue.

RNAs	Sequence
Dinitro flexizyme (dFx)	GGAUCGAAAGAUUUCCGCAUCCCCGAAAGGGUACAUGGCGUUAGGU
Enhanced flexizyme (eFx)	GGAUCGAAAGAUUUCCGCGGCCCCGAAAGGGGAUUAGCGUUAGGU
E. coli tRNA^{Ala} (UGC)	GGGGCUAUAGCUCAGCUGGGAGAGCGCCUGCUUUGCACGCAGGAGGUCUGCGGUUCGAUCCCGCAUAGCUCCACCA
P. syringae tRNA^{Cys} (GCA)	GGCCGAGUAGCAAAAUGGUUAUGCAGCGGAUUGCAAAUCCGCCUACGCCGGUUCGAUUCCGACCUCGGCCUCCA
E. coli tRNA^{Trp}	AGGGGCGUAGUUCAAUUGGUAGAGCACCGGUCUCCAAAACCGGGUGUUGGGAGUUCGAGUCUCUCCGCCCCUGCCA
T. bispora tRNA^{Glu} (CUC)	GGUCCCGUCGUCUAGAGGCCUAGGACGCCGCCCUCUCAAGGCGGUAACGGCGGUUCGAAUCCGCUCGGGACUACCA
P. syringae tRNA^{Cys} (GCA) (U71A)	GGCCGAGUAGCAAAAUGGUUAUGCAGCGGAUUGCAAAUCCGCCUACGCCGGUUCGAUUCCGACCUCGGCCACCA
P. syringae tRNA^{Cys} (GCA) (C34G/A35C/U71A)	GGCCGAGUAGCAAAAUGGUUAUGCAGCGGAUUGGCAAUCCGCCUACGCCGGUUCGAUUCCGACCUCGGCCACCA
E. coli tRNA^{Ala} (UGC) grafted with *E. coli* tRNA^{Trp} Anticodon Arm	GGGGCUAUAGCUCAGCUGGGAGAGCGCCGGUCUCCAAAACCGGAGGUCUGCGGUUCGAUCCCGCAUAGCUCCACCA
E. coli tRNA^{Ala} (UGC) grafted with *P. syringae* tRNA^{Cys} Anticodon Arm	GGGGCUAUAGCUCAGCUGGGAGAGCGGCGGAUUGCAAAUCCGCAGGUCUGCGGUUCGAUCCCGCAUAGCUCCACCA
E. coli tRNA^{Ala} (UGC) grafted with *P. syringae* tRNA^{Cys} D-Arm	GGGGCUAUAGCAAAAUGGUUAUGCACCUGCUUUGCACGCAGGAGGUCUGCGGUUCGAUCCCGCAUAGCUCCACCA
E. coli tRNA^{Ala} (UGC) grafted with *P. syringae* tRNA^{Cys} T-Stem	GGGGCUAUAGCUCAGCUGGGAGAGCGCCUGCUUUGCACGCAGGAGGUCGCCGGUUCGAUUCCGACUAGCUCCACCA
E. coli tRNA^{Gly} (GCC)	GCGGGAAUAGCUCAGUUGGUAGAGCACGACCUUGCCAAGGUCGGGGUCGCGAGUUCGAGUCUCGUUUCCCGCUCCA

RNAs	Forward Primer Sequence	Reverse Primer Sequence
Dinitro flexizyme (dFx)	GGCGTAATACGACTCACTATAGGATCGAAAGATTTCCG	mAmCCTAACGCCATGTACCCTTTCGGGGATGCGGAAATCTTTCGATC
Enhanced flexizyme (eFx)	GGCGTAATACGACTCACTATAGGATCGAAAGATTTCCGCG	mAmCCTAACGCTAATCCCCTTTCGGGGCCGCGGAAATCTTTCG

E. coli tRNA^{Ala} (UGC)	AATTCCTGCAGTAATACGACTCACTATAGGGGCTATAGCTCAGCTGGGAGAGCGCCTGC	mUmGGTGGAGCTATGCGGGATCGAACCGCAGACCTCCTGCGTGCAAAGCAGGCGCTCTCCCAG
P. syringae tRNA^{Cys} (GCA)	AATTCCTGCAGTAATACGACTCACTATAGGCCGAGTAGCAAAATGGTTATGCAGC	mUmGGAGGCCGAGGTCGGAATCGAACCGGCGTAGGCGGATTTGCAATCCGCTGCATAACC
E. coli tRNA^{Trp}	AATTCCTGCAGTAATACGACTCACTATAAGGGGCGTAGTTCAATTGGTAGAGCACCGGTC	mUmGGCAGGGGCGGAGAGACTCGAACTCCCAACACCCGGTTTTGGAGACCGGTGCTCTACC
T. bispora tRNA^{Glu} (CUC)	AATTCCTGCAGTAATACGACTCACTATAGGTCCCGTCGTCTAGAGGCCTAGGACGC	mUmGGTAGTCCCGAGCGGATTCGAACCGCCGTTACCGCCTTGAGAGGGCGGCGTCCTAGG
P. syringae tRNA^{Cys} (GCA) (U71A)	AATTCCTGCAGTAATACGACTCACTATAGGCCGAGTAGCAAAATGGTTATGCAGC	mUmGGTGGCCGAGGTCGGAATCGAACCGGCGTAGGCGGATTTGCAATCCGCTGCATAACC
P. syringae tRNA^{Cys} (GCA) (C34G/A35C/U71A)	AATTCCTGCAGTAATACGACTCACTATAGGCCGAGTAGCAAAATGGTTATGCAGC	mUmGGTGGCCGAGGTCGGAATCGAACCGGCGTAGGCGGATTGCCAATCCGCTGCATAACC
E. coli tRNA^{Ala} (UGC) grafted with *E. coli* tRNA^{Trp} Anticodon Arm	AATTCCTGCAGTAATACGACTCACTATAGGGGCTATAGCTCAGCTGGGAGAGCGCCGGT	mUmGGTGGAGCTATGCGGGATCGAACCGCAGACCTCCGGTTTTGGAGACCGGCGCTCTCCCAG
E. coli tRNA^{Ala} (UGC) grafted with *P. syringae* tRNA^{Cys} Anticodon Arm	AATTCCTGCAGTAATACGACTCACTATAGGGGCTATAGCTCAGCTGGGAGAGCGGCGGA	mUmGGTGGAGCTATGCGGGATCGAACCGCAGACCTGCGGATTTGCAATCCGCCGCTCTCCCAG
E. coli tRNA^{Ala} (UGC) grafted with *P. syringae* tRNA^{Cys} D-Arm	AATTCCTGCAGTAATACGACTCACTATAGGGGCTATAGCAAAATGGTTATGCACCTGCT	mUmGGTGGAGCTATGCGGGATCGAACCGCAGACCTCCTGCGTGCAAAGCAGGTGCATAACCAT
E. coli tRNA^{Ala} (UGC) grafted with *P. syringae* tRNA^{Cys} T-Stem	AATTCCTGCAGTAATACGACTCACTATAGGGGCTATAGCTCAGCTGGGAGAGCGCCTGC	mUmGGTGGAGCTAGTCGGAATCGAACCGGCGACCTCCTGCGTGCAAAGCAGGCGCTCTCCCAG
E. coli tRNA^{Gly} (GCC)	AATTCCTGCAGTAATACGACTCACTATAGCGGGAATAGCTCAGTTGGTAGAGCACGACC	mUmGGAGCGGGAAACGAGACTCGAACTCGCGACCCCGACCTTGGCAAGGTCGTGCTCTACCAA

Table S5. Sequences of the Cy3-labeled primers used for the $NaIO_4$-RNA extension assays. All sequences are provided 5' to 3' (left to right). Cy3 was incorporated at the 3'-OH of these primers.

RNAs	Cy3 Primer Sequence
E. coli $tRNA^{Ala}$ (UGC)	TTAAAAAAAAAAAAAAAAAAAAAAAAAAAATGGTGGAGCTATGC GGGATCGAACCGCAGACC
P. syringae $tRNA^{Cys}$ (GCA)	TTAAAAAAAAAAAAAAAAAAAAAAAAAAAATGGAGGCCGAGGTC GGAATCGAACCGGCGTAG
E. coli $tRNA^{Trp}$	TTAAAAAAAAAAAAAAAAAAAAAAAAAAAATGGCAGGGGCGGAG AGACTCGAACTCCCAACA
T. bispora $tRNA^{Glu}$ (CUC)	TTAAAAAAAAAAAAAAAAAAAAAAAAAAAATGGTAGTCCCGAGC GGATTCGAACCGCCGTTA
P. syringae $tRNA^{Cys}$ (GCA) (U71A)	TTAAAAAAAAAAAAAAAAAAAAAAAAAAAATGGAGGCCGAGGTC GGAATCGAACCGGCGTAG
P. syringae $tRNA^{Cys}$ (GCA) (C34G/A35C/U71A)	TTAAAAAAAAAAAAAAAAAAAAAAAAAAAATGGTGGCCGAGGTC GGAATCGAACCGGCGTAG
E. coli $tRNA^{Ala}$ (UGC) grafted with *E. coli* $tRNA^{Trp}$ Anticodon Arm	TTAAAAAAAAAAAAAAAAAAAAAAAAAAAATGGTGGAGCTATGC GGGATCGAACCGCAGACC
E. coli $tRNA^{Ala}$ (UGC) grafted with *P. syringae* $tRNA^{Cys}$ Anticodon Arm	TTAAAAAAAAAAAAAAAAAAAAAAAAAAAATGGTGGAGCTATGC GGGATCGAACCGCAGACC
E. coli $tRNA^{Ala}$ (UGC) grafted with *P. syringae* $tRNA^{Cys}$ D-Arm	TTAAAAAAAAAAAAAAAAAAAAAAAAAAAATGGTGGAGCTATGC GGGATCGAACCGCAGACC
E. coli $tRNA^{Ala}$ (UGC) grafted with *P. syringae* $tRNA^{Cys}$ T-Stem	TTAAAAAAAAAAAAAAAATGGTGGAGCTAGTCGGAATCGAACCG GCGACCTCCTGCGTGC
E. coli $tRNA^{Gly}$ (GCC)	TTAAAAAAAAAAAAAAAAAAAAAAAAAAAATGGAGCGGGAAACG AGACTCGAACTCGCGACC

Table S6. Calculated and observed *m/z* values for products and starting materials in this study.

**Peptide**	**Calculated *m/z* (z =2)**	**Observed *m/z***	**Error (ppm)**
Δ19BhaA	1137.0753	1137.0764	0.97
Δ19BhaA-Ala	1172.5939	1172.5972	2.81
Δ19BhaA-Lac	1173.0859	1173.0862	0.28
Δ19BhaA-Gln	1201.1046	1201.1088	3.49
Δ19BhaA-Glu	1201.5966	1201.5970	0.32
Δ19BhaA-(N-Me-Ala)	1179.6017	No product observed	
Δ19BhaA-Trp	1230.1150	1230.1165	1.24
Δ19BhaA-ThioGly	1174.0667	1174.0672	0.43
Δ19BhaA-Cysteamine	1166.5851	1166.5861	0.88
Δ19BhaA-Gly	1165.5861	1165.5866	0.47
Δ19BhaA-Phe	1210.6095	1210.6115	1.63
Δ19BhaA-(β-Ala)	1172.5939	1172.5970	2.64
Δ19BhaA-(D-Ala)	1172.5939	1172.5963	2.05
Δ19BhaA-Aib	1179.6017	No product observed	

REFERENCES

(1) Sherlin, L. D.; Bullock, T. L.; Nissan, T. A.; Perona, J. J.; Lariviere, F. J.; Uhlenbeck, O. C.; Scaringe, S. A. Chemical and enzymatic synthesis of tRNAs for high-throughput crystallization. *RNA* **2001**, *7*, 1671-8.

(2) Murakami, H.; Ohta, A.; Ashigai, H.; Suga, H. A highly flexible tRNA acylation method for non-natural polypeptide synthesis. *Nature Methods* **2006**, *3*, 357-9.

(3) Goto, Y.; Katoh, T.; Suga, H. Flexizymes for genetic code reprogramming. *Nat. Protoc.* **2011**, *6*, 779-90.

(4) Fujino, T.; Goto, Y.; Suga, H.; Murakami, H. Reevaluation of the D-amino acid compatibility with the elongation event in translation. *J. Am. Chem. Soc.* **2013**, *135*, 1830-7.

(5) Katoh, T.; Iwane, Y.; Suga, H. Logical engineering of D-arm and T-stem of tRNA that enhances d-amino acid incorporation. *Nucleic Acids Res.* **2017**, *45*, 12601-10.

(6) Kawakami, T.; Murakami, H.; Suga, H. Messenger RNA-programmed incorporation of multiple N-methyl-amino acids into linear and cyclic peptides. *Chem. Biol.* **2008**, *15*, 32-42.

(7) Ohta, A.; Murakami, H.; Higashimura, E.; Suga, H. Synthesis of polyester by means of genetic code reprogramming. *Chem. Biol.* **2007**, *14*, 1315-22.

(8) Takatsuji, R.; Shinbara, K.; Katoh, T.; Goto, Y.; Passioura, T.; Yajima, R.; Komatsu, Y.; Suga, H. Ribosomal synthesis of backbone-cyclic peptides compatible with in vitro display. *J. Am. Chem. Soc.* **2019**, *141*, 2279-87.

(9) Cervettini, D.; Tang, S.; Fried, S. D.; Willis, J. C. W.; Funke, L. F. H.; Colwell, L. J.; Chin, J. W. Rapid discovery and evolution of orthogonal aminoacyl-tRNA synthetase-tRNA pairs. *Nat. Biotechnol.* **2020**, *38*, 989-99.

(10) Dunkelmann, D. L.; Piedrafita, C.; Dickson, A.; Liu, K. C.; Elliott, T. S.; Fiedler, M.; Bellini, D.; Zhou, A.; Cervettini, D.; Chin, J. W. Adding α,α-disubstituted and β-linked monomers to the genetic code of an organism. *Nature* **2024**, *625*, 603-10.

(11) Soni, C.; Prywes, N.; Hall, M.; Nair, M. A.; Savage, D. F.; Schepartz, A.; Chatterjee, A. A translation-independent directed evolution strategy to engineer aminoacyl-tRNA synthetases. *ACS Cent. Sci.* **2024**, *10*, 1211-20.

(12) Malz, F.; Jancke, H. Validation of quantitative NMR. *J. Pharm. Biomed. Anal.* **2005**, *38*, 813-23.

(13) Daniels, P. N.; Lee, H.; Splain, R. A.; Ting, C. P.; Zhu, L.; Zhao, X.; Moore, B. S.; van der Donk, W. A. A biosynthetic pathway to aromatic amines that uses glycyl-tRNA as nitrogen donor. *Nat. Chem.* **2022**, *14*, 71-7.

(14) Vinogradov, A. A.; Shimomura, M.; Kano, N.; Goto, Y.; Onaka, H.; Suga, H. Promiscuous enzymes cooperate at the substrate level en route to lactazole A. *J. Am. Chem. Soc.* **2020**, *142*, 13886-97.

(15) Vinogradov, A. A.; Shimomura, M.; Goto, Y.; Ozaki, T.; Asamizu, S.; Sugai, Y.; Suga, H.; Onaka, H. Minimal lactazole scaffold for in vitro thiopeptide bioengineering. *Nat. Commun.* **2020**, *11*, 2272.

(16) Ho, C. S.; Lam, C. W.; Chan, M. H.; Cheung, R. C.; Law, L. K.; Lit, L. C.; Ng, K. F.; Suen, M. W.; Tai, H. L. Electrospray ionisation mass spectrometry: principles and clinical applications. *Clin. Biochem. Rev.* **2003**, *24*, 3-12.

(17) Pitt, J. J. Principles and applications of liquid chromatography-mass spectrometry in clinical biochemistry. *Clin. Biochem. Rev.* **2009**, *30*, 19-34.

(18) Paizs, B.; Suhai, S. Fragmentation pathways of protonated peptides. *Mass Spectrom. Rev* **2005**, *24*, 508-48.

(19) Kaufmann, R.; Kirsch, D.; Spengler, B. Sequenching of peptides in a time-of-flight mass spectrometer: evaluation of postsource decay following matrix-assisted laser desorption ionisation (MALDI). *Int. J. Mass Spectrom. Ion Processes* **1994**, *131*, 355-85.
(20) Dawson, P. E.; Muir, T. W.; Clark-Lewis, I.; Kent, S. B. Synthesis of proteins by native chemical ligation. *Science* **1994**, *266*, 776-9.
(21) Chan, P. P.; Lin, B. Y.; Mak, A. J.; Lowe, T. M. tRNAscan-SE 2.0: improved detection and functional classification of transfer RNA genes. *Nucleic Acids Res.* **2021**, *49*, 9077-96.
(22) Kerpedjiev, P.; Hammer, S.; Hofacker, I. L. Forna (force-directed RNA): Simple and effective online RNA secondary structure diagrams. *Bioinformatics* **2015**, *31*, 3377-9.
(23) Raden, M.; Ali, S. M.; Alkhnbashi, O. S.; Busch, A.; Costa, F.; Davis, J. A.; Eggenhofer, F.; Gelhausen, R.; Georg, J.; Heyne, S.; Hiller, M.; Kundu, K.; Kleinkauf, R.; Lott, S. C.; Mohamed, M. M.; Mattheis, A.; Miladi, M.; Richter, A. S.; Will, S.; Wolff, J.; Wright, P. R.; Backofen, R. Freiburg RNA tools: a central online resource for RNA-focused research and teaching. *Nucleic Acids Res.* **2018**, *46*, W25-W9.
(24) Will, S.; Joshi, T.; Hofacker, I. L.; Stadler, P. F.; Backofen, R. LocARNA-P: accurate boundary prediction and improved detection of structural RNAs. *RNA* **2012**, *18*, 900-14.
(25) Will, S.; Reiche, K.; Hofacker, I. L.; Stadler, P. F.; Backofen, R. Inferring Noncoding RNA Families and Classes by Means of Genome-Scale Structure-Based Clustering. *PLoS Comput. Biol.* **2007**, *3*, e65.
(26) Pettersen, E. F.; Goddard, T. D.; Huang, C. C.; Meng, E. C.; Couch, G. S.; Croll, T. I.; Morris, J. H.; Ferrin, T. E. UCSF ChimeraX: Structure visualization for researchers, educators, and developers. *Protein Sci.* **2021**, *30*, 70-82.
